# Supplementary material for: Sex affects transcriptional associations with schizophrenia across the dorsolateral prefrontal cortex, hippocampus, and caudate nucleus
Source: Nat Commun. 2024 May 10;15:3980. doi: 10.1038/s41467-024-48048-z (PMC11087501; doi:10.1038/s41467-024-48048-z)
Supplement: Supplementary file 1 — Supplementary Information [file 41467_2024_48048_MOESM1_ESM.pdf]

Supplementary Information

Figures

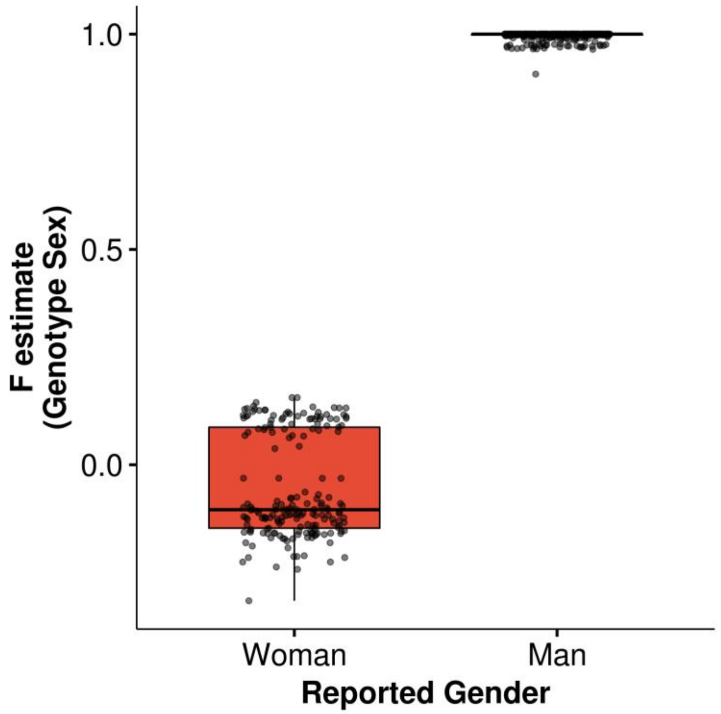

Fig. S1. Completed overlap of reported gender with sex genotype. Box plot showing F estimates of genotype sex (n=504) for female and male individuals correlate with reported gender (i.e., woman or man). Box plots show the median and first and third quartiles, and whiskers extend to 1.5× the interquartile range.

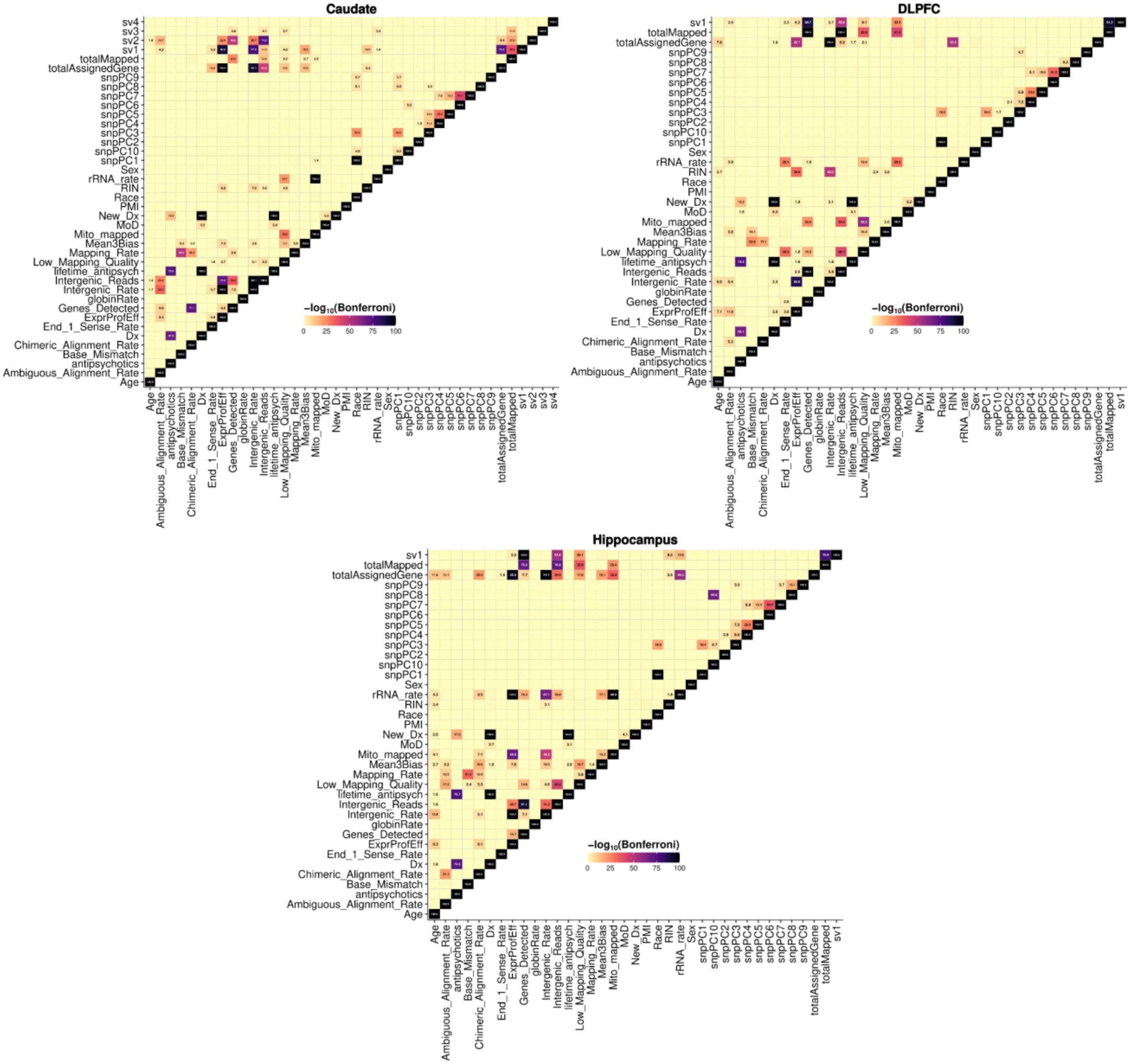

Fig. S2. High correlation between covariates across brain regions. Heatmap showing correlation between covariates across brain regions (linear regression, Bonferroni corrected p-values). Significant correlations (-$\log_{10}$ transformed, Bonferroni corrected p-values) are denoted in each tile. Empty tiles do not show a significant correlation.

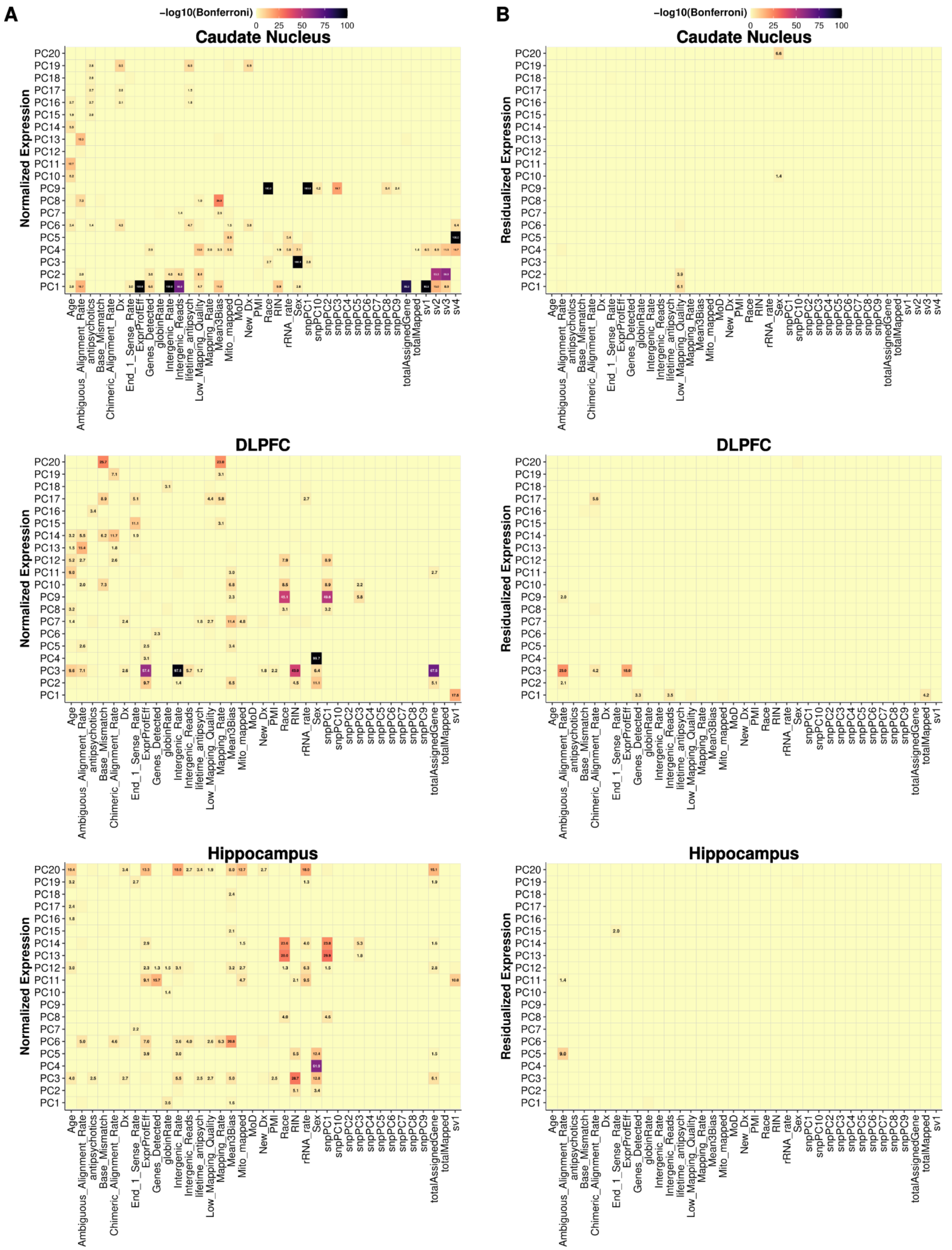

Fig. S3. Spurious correlations reduced after regressing out general model covariates. Heatmaps showing correlation between principal component analysis of **A.** normalized gene expression or **B.** residualized gene expression. A value of 1.3 or greater is significant and equivalent to Bonferroni corrected p-value < 0.05. Significant correlations are denoted within each tile.

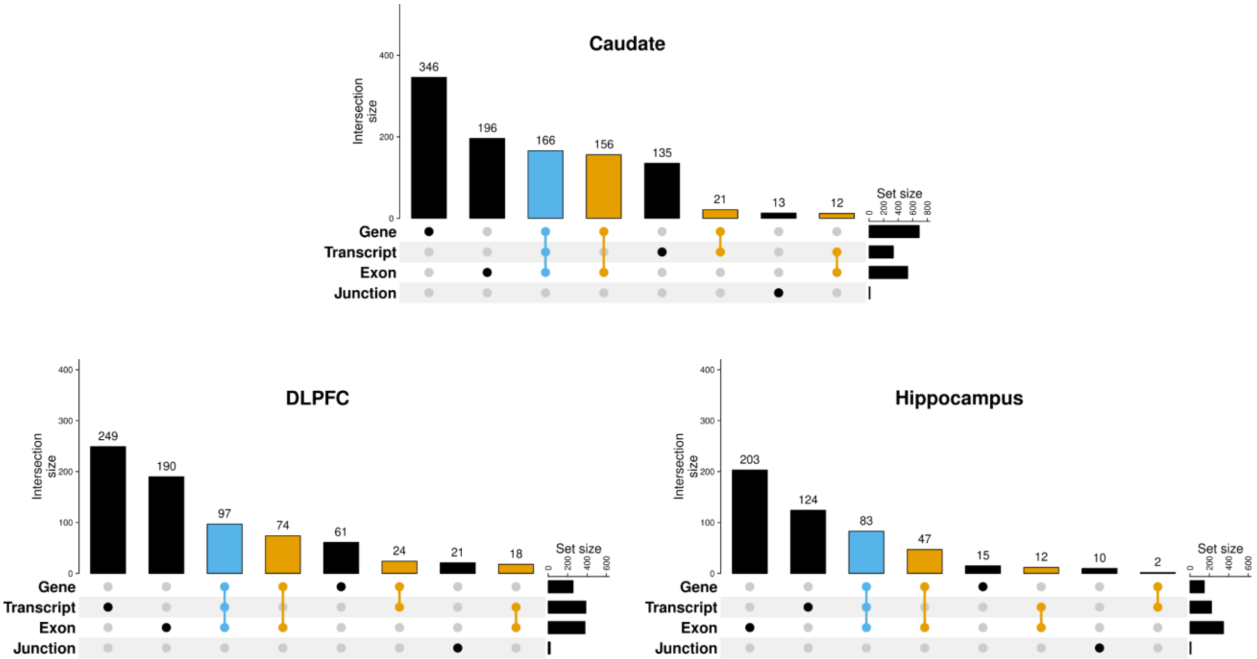

Fig. S4. Isoform level analysis increases the detection of sex-biased genes. Blue, shared across three brain regions; orange, shared between two brain regions; and black, unique to a specific brain region. Novel junctions not annotated to unique gene ID.

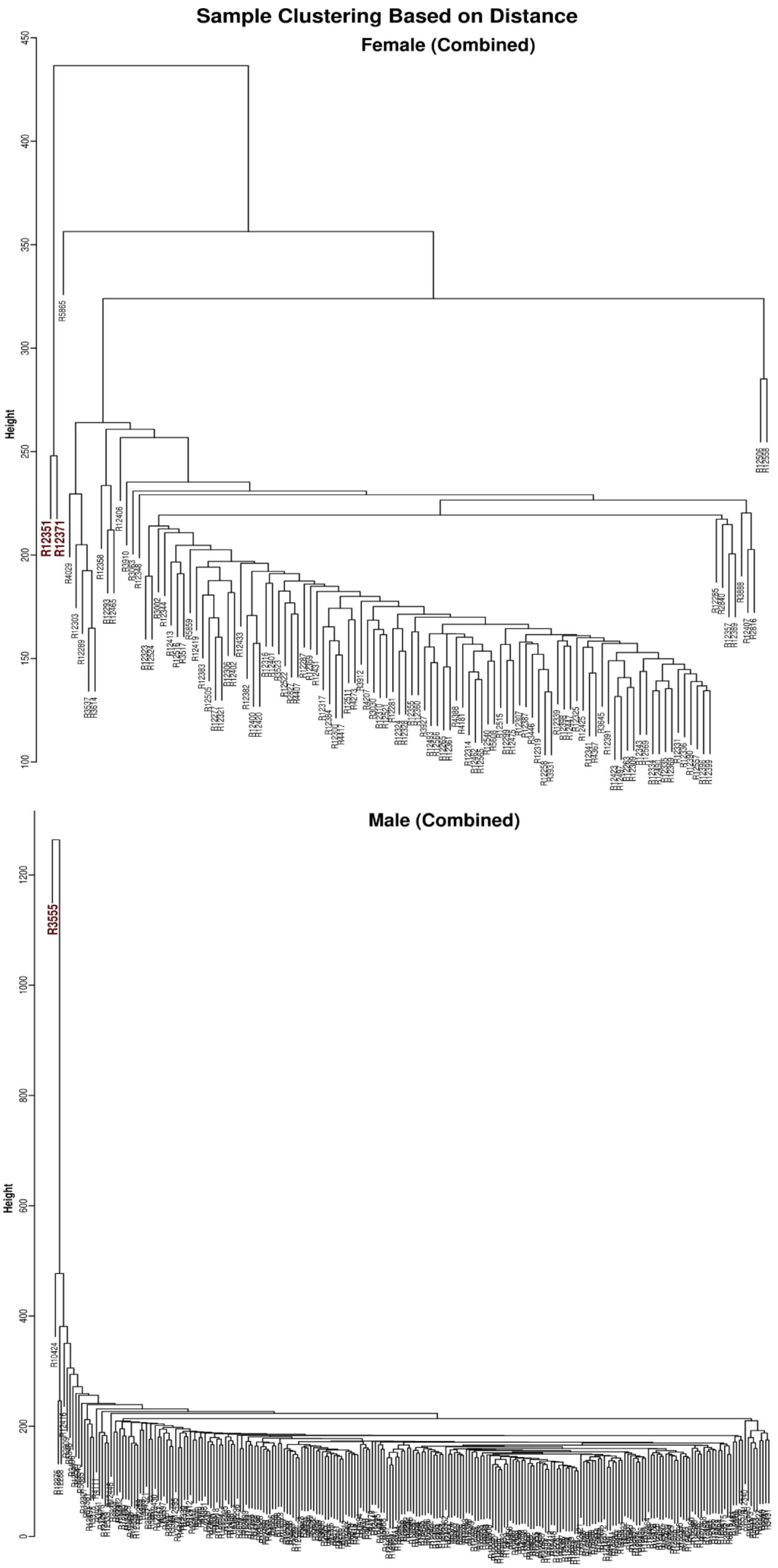

Fig. S5. Sample dendrogram showing outlier individuals for the DLPFC. Removed samples highlighted in red.

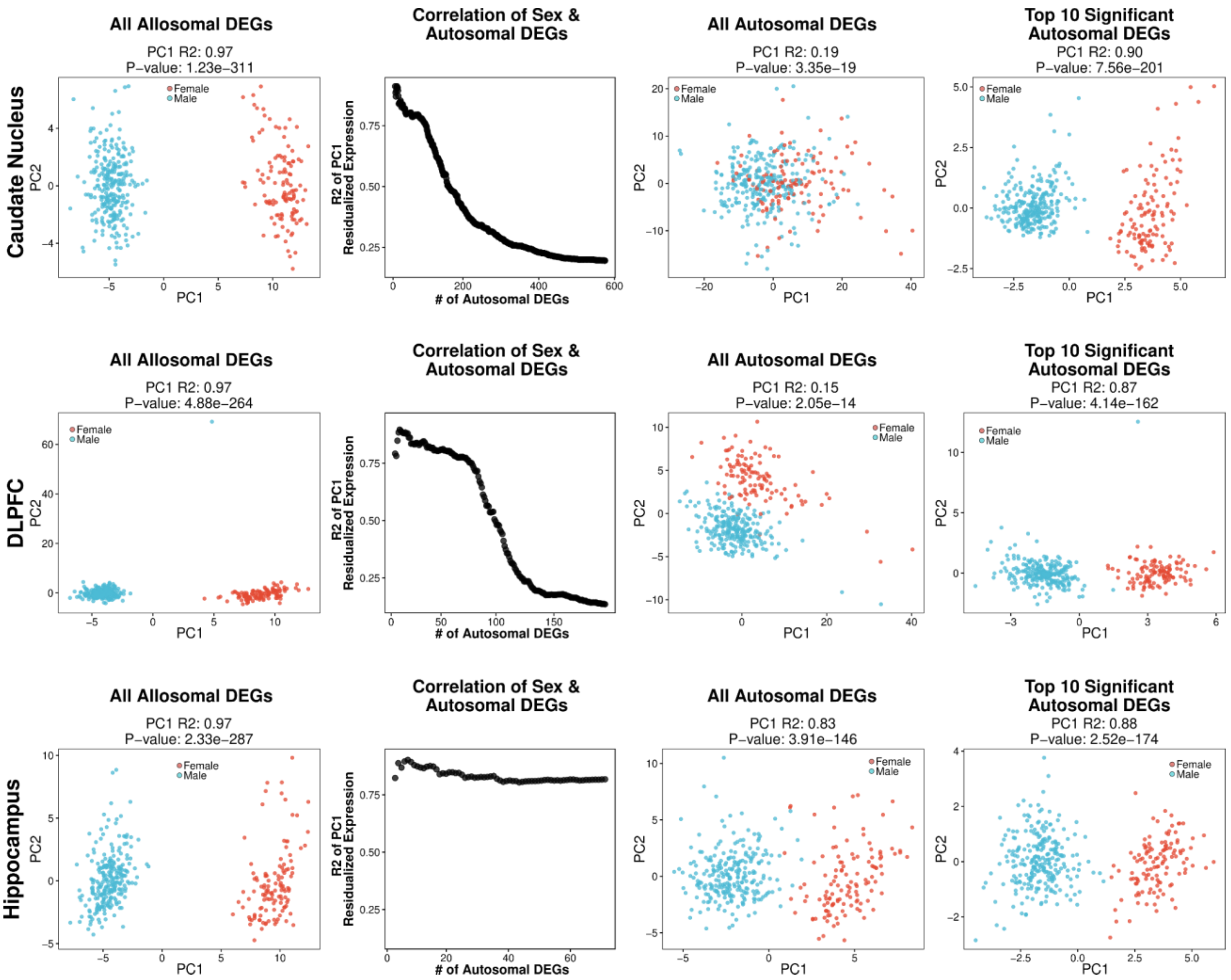

Fig. S6. Autosomal sex-specific DEGs show significant correlation with sex and strong predictive power for sex in the brain. Scatterplots of principal components (PC) 1 and 2 from dimensionally reduced expression of all allosomal and autosomal DEGs for the caudate nucleus, DLPFC, and hippocampus. Plots are annotated with parametric correlation (R^2) from linear regression for PC1.

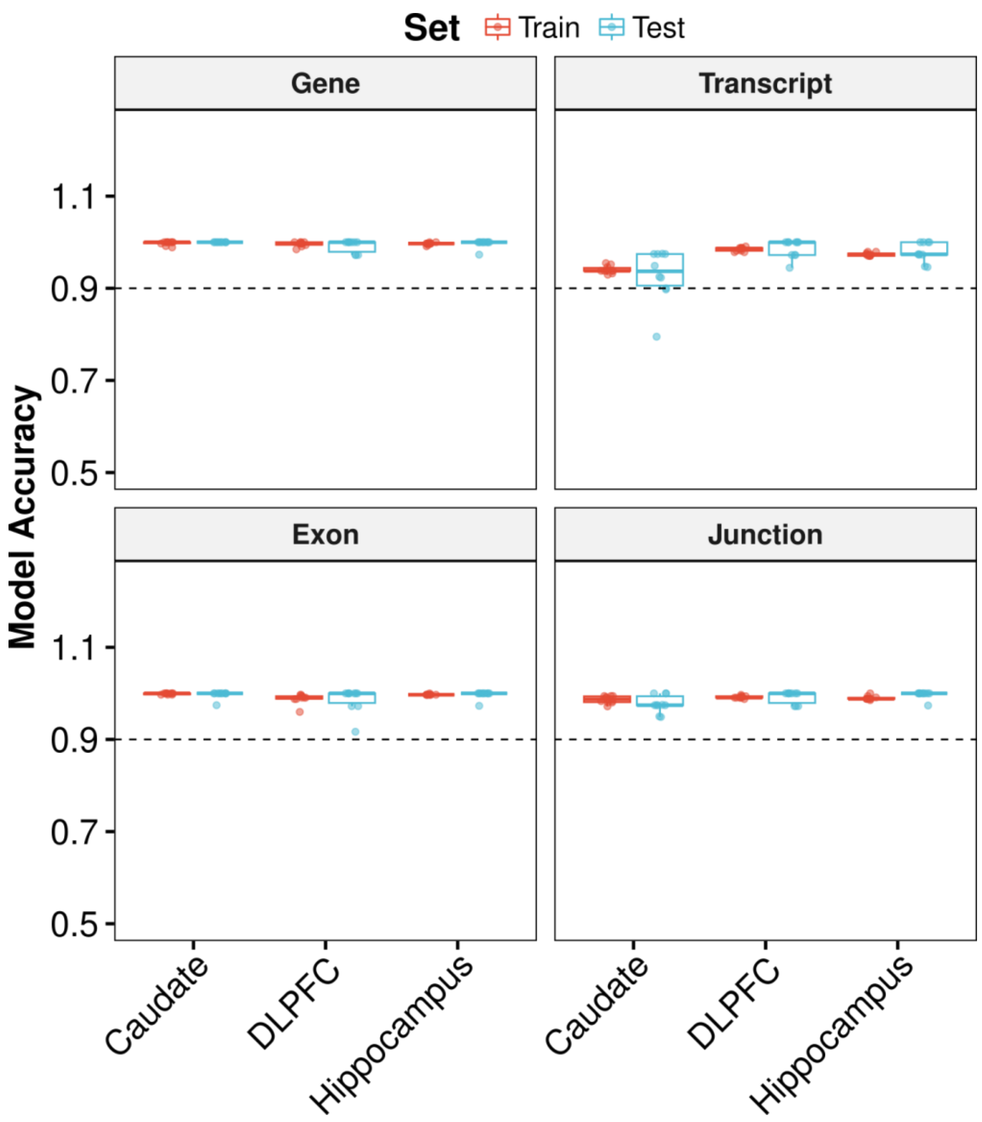

Fig. S7. Metric summary across brain regions and features show high classification accuracy for sex using autosomes. Box plot of train (red) and test (blue) accuracy for sex classification in the caudate nucleus (n=393; 121 female and 272 male), DLPFC (dorsolateral prefrontal cortex; n=359; 114 female and 245 male), and hippocampus (n=375; 121 female and 254 male) for genes, transcripts, exons, and junctions. Each point represents results from one fold within the 10-fold cross-validation. Dashed line denotes 90% accuracy. Box plots show the median and first and third quartiles, and whiskers extend to 1.5× the interquartile range.

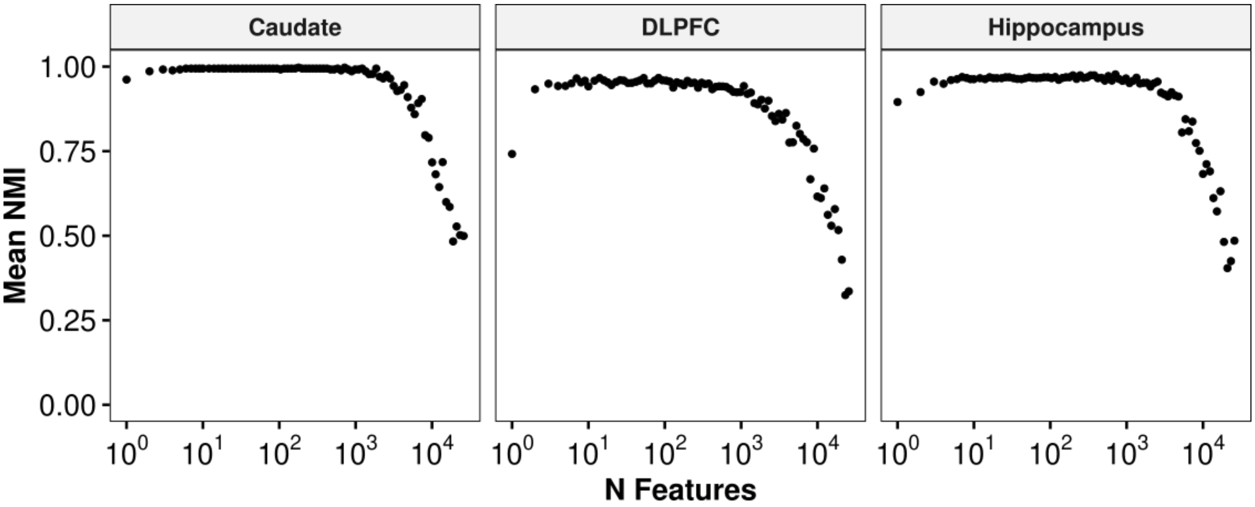

Fig. S8. Dynamic recursive feature elimination curve shows prediction is driven primarily by one gene. Scatterplot showing mean normalized mutual information (NMI) training score across ten folds as a function of *N* features (x-axis, log_{10} scale).

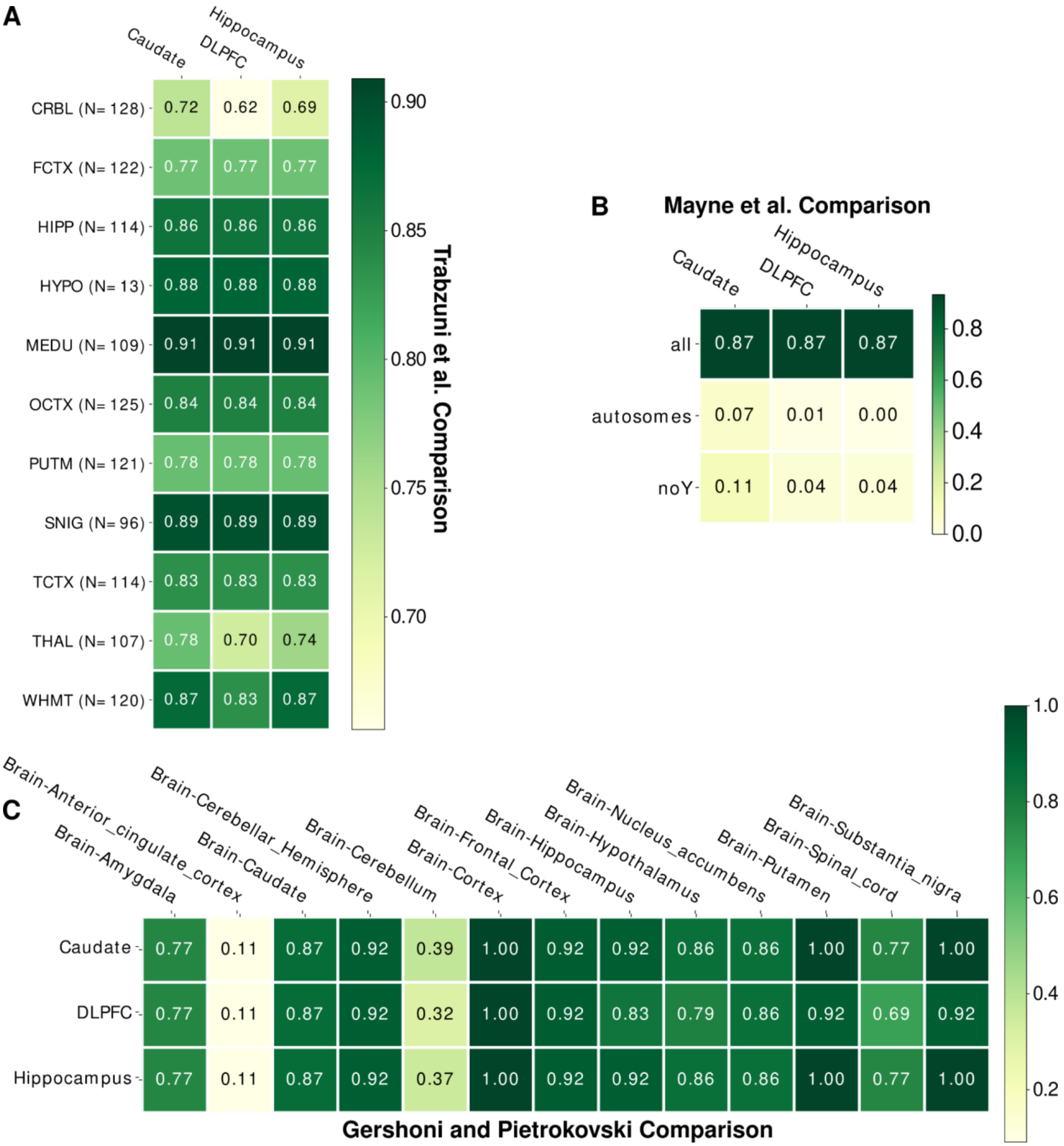

Fig. S9. High overlap of sex-specific differentially expressed genes (DEGs) across multiple datasets and brain regions. Heatmaps comparing BrainSeq Consortium brain regions that show the ratio of DEGs overlap with **A.** Trabzuni et al. brain regions, **B.** Mayne et al. meta analysis with significant expression in at least one brain region using all chromosomes, only autosomes, or no Y chromosomes, and **C.** Gershoni and Pietrokovski brain regions. Abbreviations: CRBL: cerebellum, FCTX: frontal cortex, HIPP: hippocampus, HYPO: hypothalamus, MEDU: medulla, OCTX: occipital cortex, PUTM: putamen, SNIG: substantia nigra, TCTX: temporal cortex, THAL: thalamus, and WHMT: white matter.

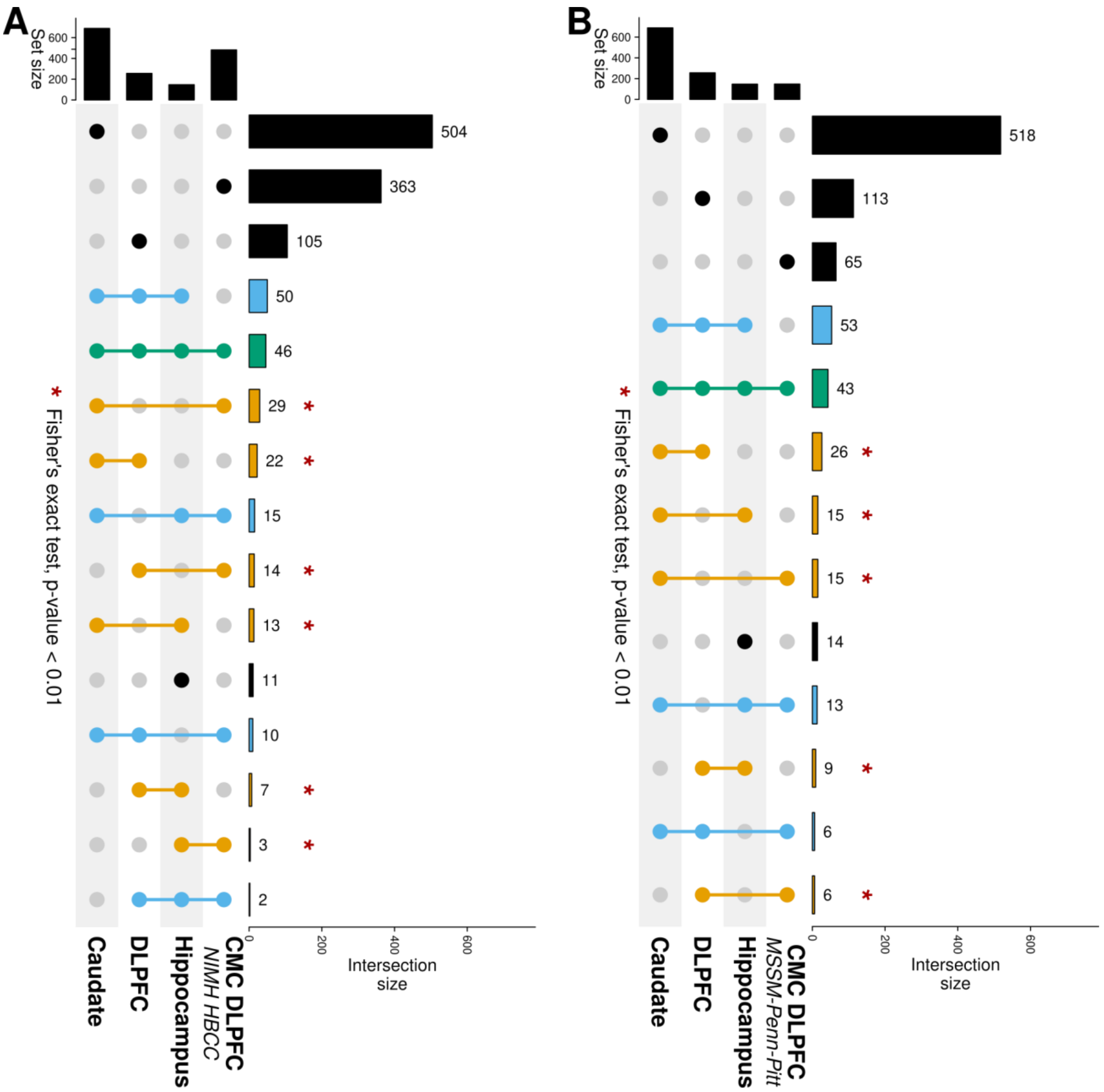

Fig. S10. Significant sharing of sex-specific differentially expressed genes (DEGs) across brain regions replicate in the CommonMind Consortium (CMC) DLPFC. UpSet plot showing number of DEGs shared across brain regions with the CMC DLPFC cohort **A.** NIMH HBCC and **B.** MSSM-Penn-Pitt. ***** Indicating p-value < 0.01 for two-tailed, Fisher's exact test. Green is shared across the four brain regions; blue, shared across three brain regions; orange, shared between two brain regions; and black, unique to a specific brain region. Exact p-values are available in the Source Data.

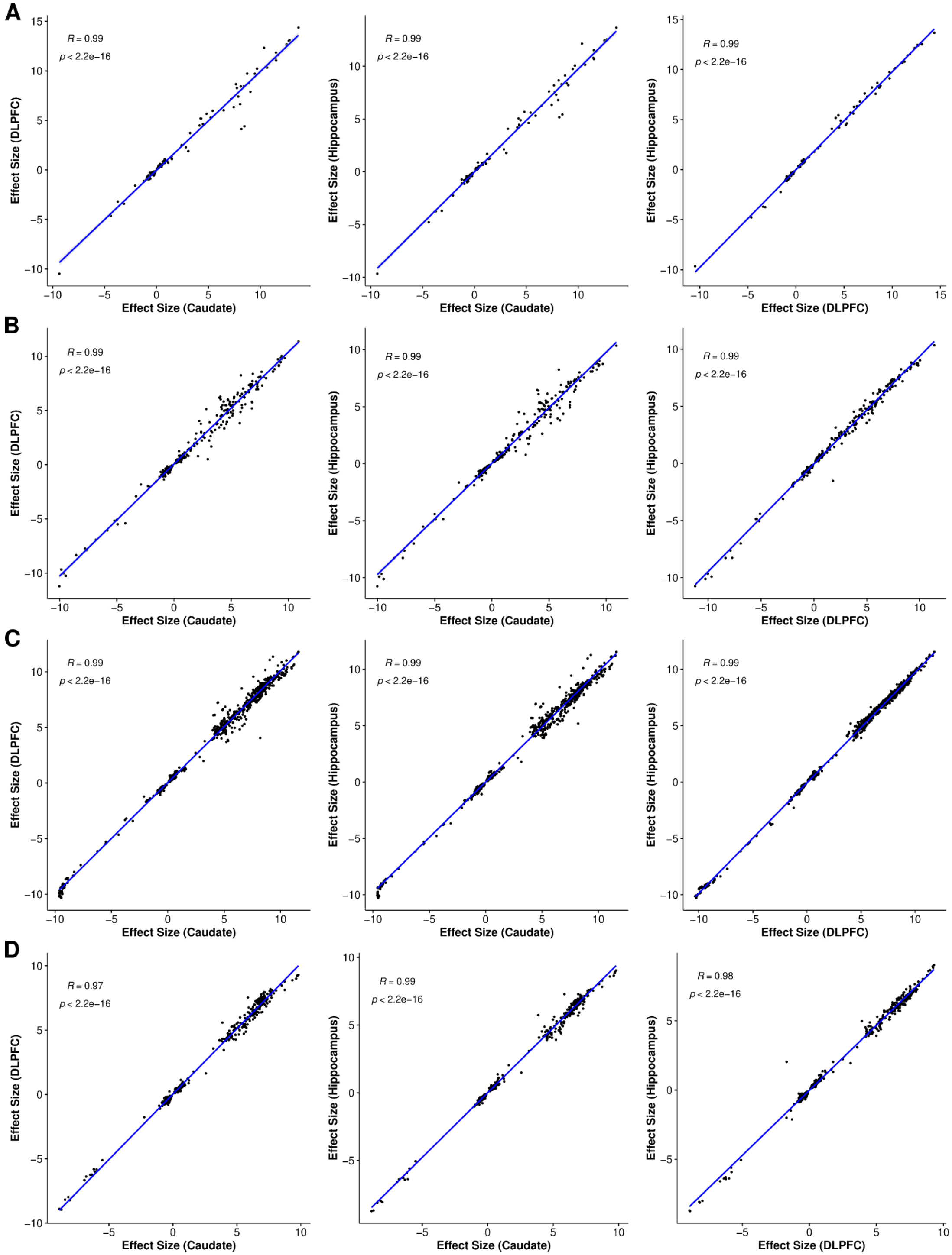

Fig. S11. All significantly differentially expressed features (FDR < 0.05) have concordant directionality for sex differences across the three brain regions. Scatterplot of Spearman correlation comparing effect size (logFC) from sex differentially expressed features (adjusted p-value < 0.05) between brain region pairs for **A**. genes, **B**. transcripts, **C.** exons, and **D**. exon-exon junctions. A fitted trend line is presented in blue as the mean values +/- standard deviation. Exact p-values are available in the Source Data.

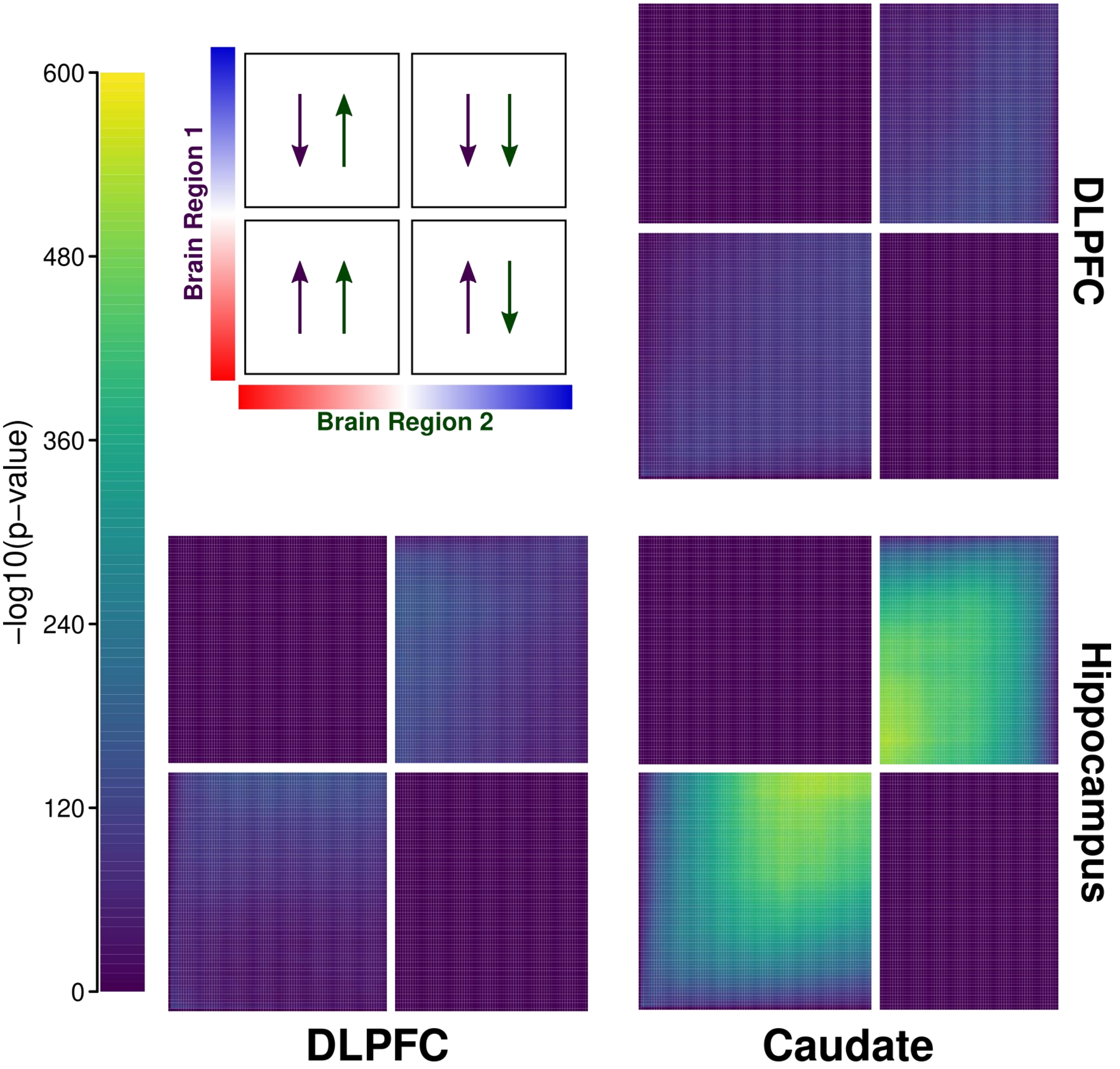

Fig. S12. Concordant transcriptional changes for sex-specific expression across the brain. RRHO (rank-rank hypergeometric overlap) maps comparing sex-specific transcriptional changes for all genes between brain region pairs stratified by direction of effect. There are no genes with discordant direction of effect. The panel presents the overlapping relationship between two brain regions. The color bar represents the degree of significance [-log10(p-value)] of overlap between two brain regions. Arrows show the direction of effect for sex (female biased [downregulated] or male biased [upregulated]) by brain region.

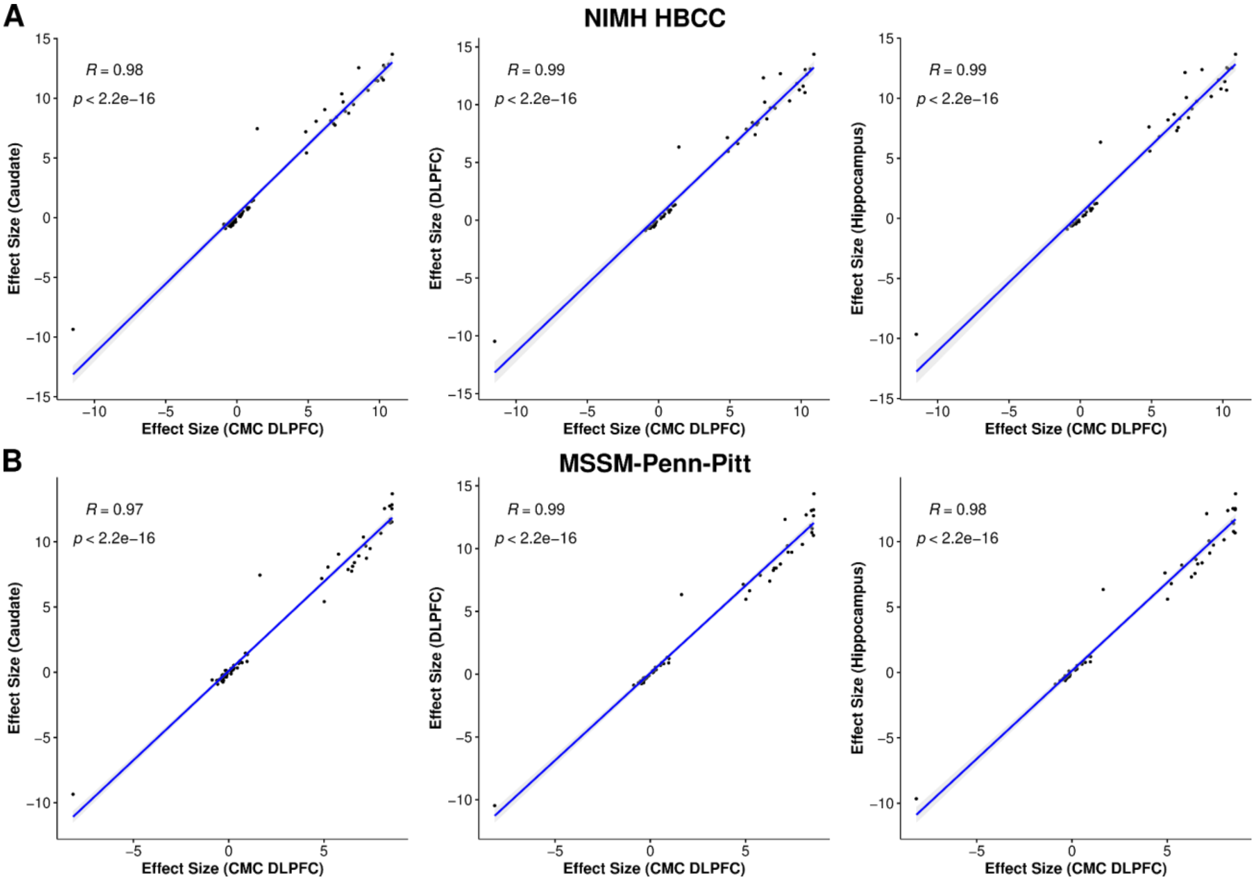

Fig. S13. Replication of concordant directionality of sex DEGs with CommonMind (CMC) DLPFC. Scatterplot comparing effect size (logFC) between BrainSeq Consortium brain regions (caudate nucleus, DLPFC, and hippocampus) and CMC DLPFC cohort **A.** NIMH HBCC and **B.** MSSM-Penn-Pitt. A fitted trend line is presented in blue as the mean values +/- standard deviation. The standard deviation is shaded in light gray. Exact p-values are available in the Source Data.

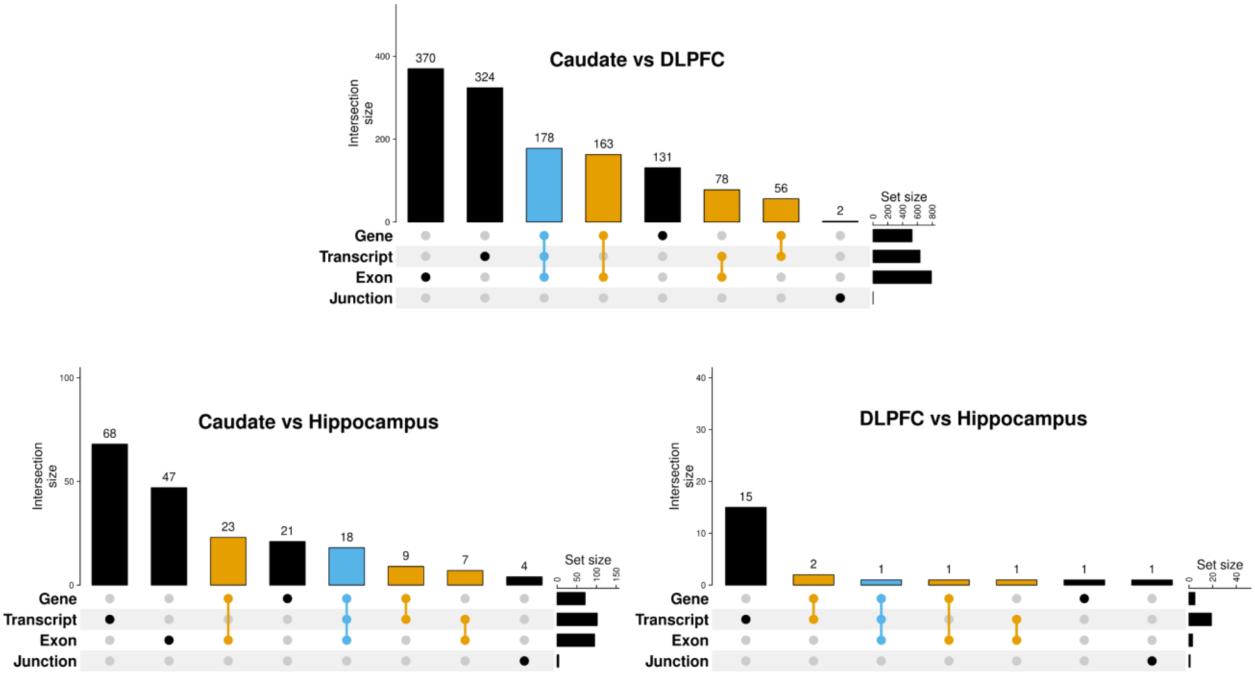

Fig. S14. Unique differentially expressed genes (DEGs) double with the inclusion of isoform-level analysis. Blue, shared across three brain regions; orange, shared between two brain regions; and black, unique to a specific brain region. Novel junctions not annotated to unique gene ID.

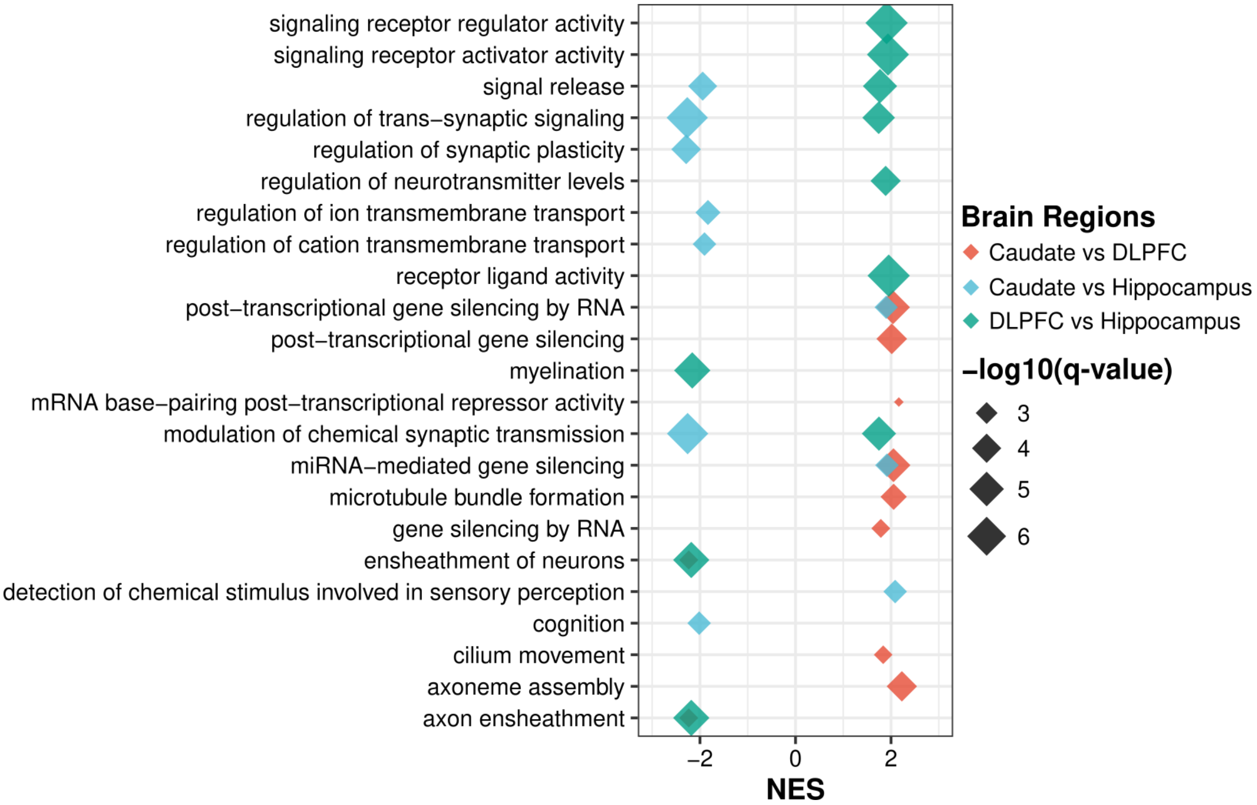

Fig. S15. Brain region-specific transcriptional sex differences show significant enrichment for myelination, cognition, and gene silencing. Gene set enrichment analysis (GSEA) of brain region-specific sex differential expression analysis between brain regions. NES: normalized enrichment score.

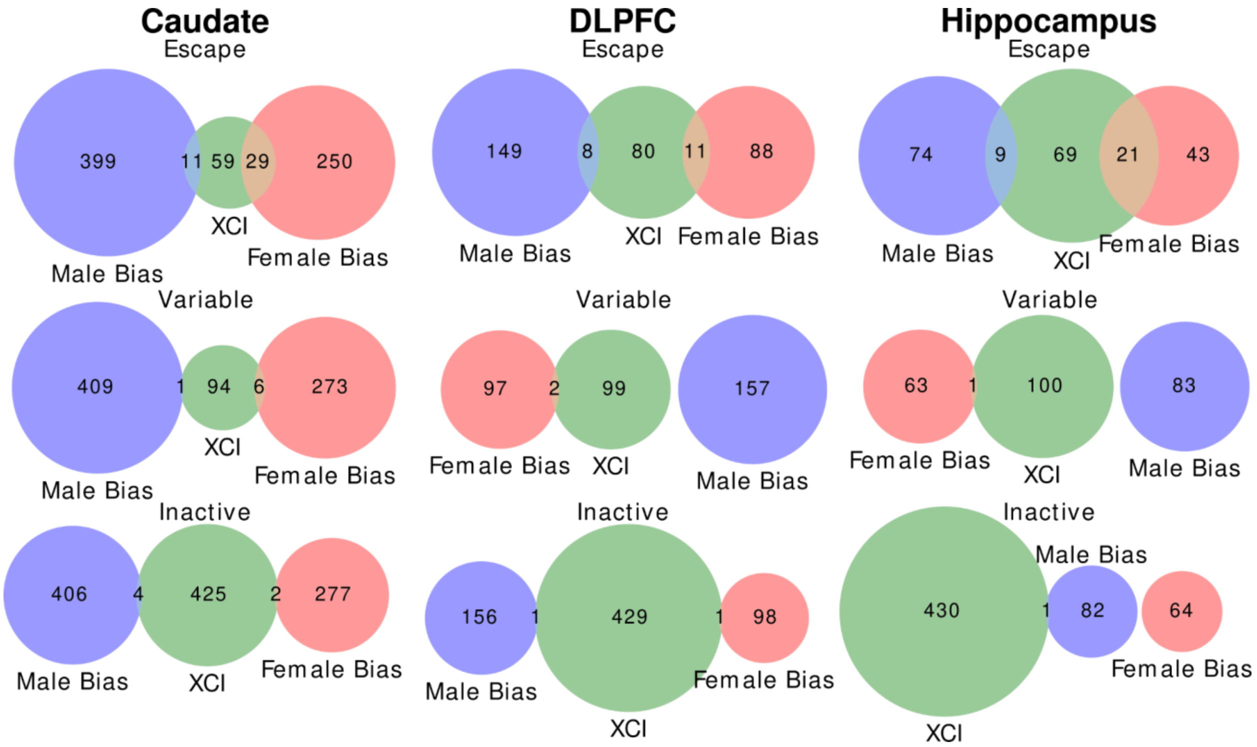

Fig. S16. Large overlap of escaping X-chromosome inactivation (XCI) with sex-specific differentially expressed genes (DEGs). Venn diagram demonstrating large overlap of escaping X-chromosome inactivation (XCI) genes with sex-specific differentially expressed genes (DEGs) upregulated in female individuals (female bias; red) compared with variable and inactive XCI genes for the caudate nucleus, DLPFC, and hippocampus. DEGs upregulated in male individuals (male bias; blue). XCI genes in green.

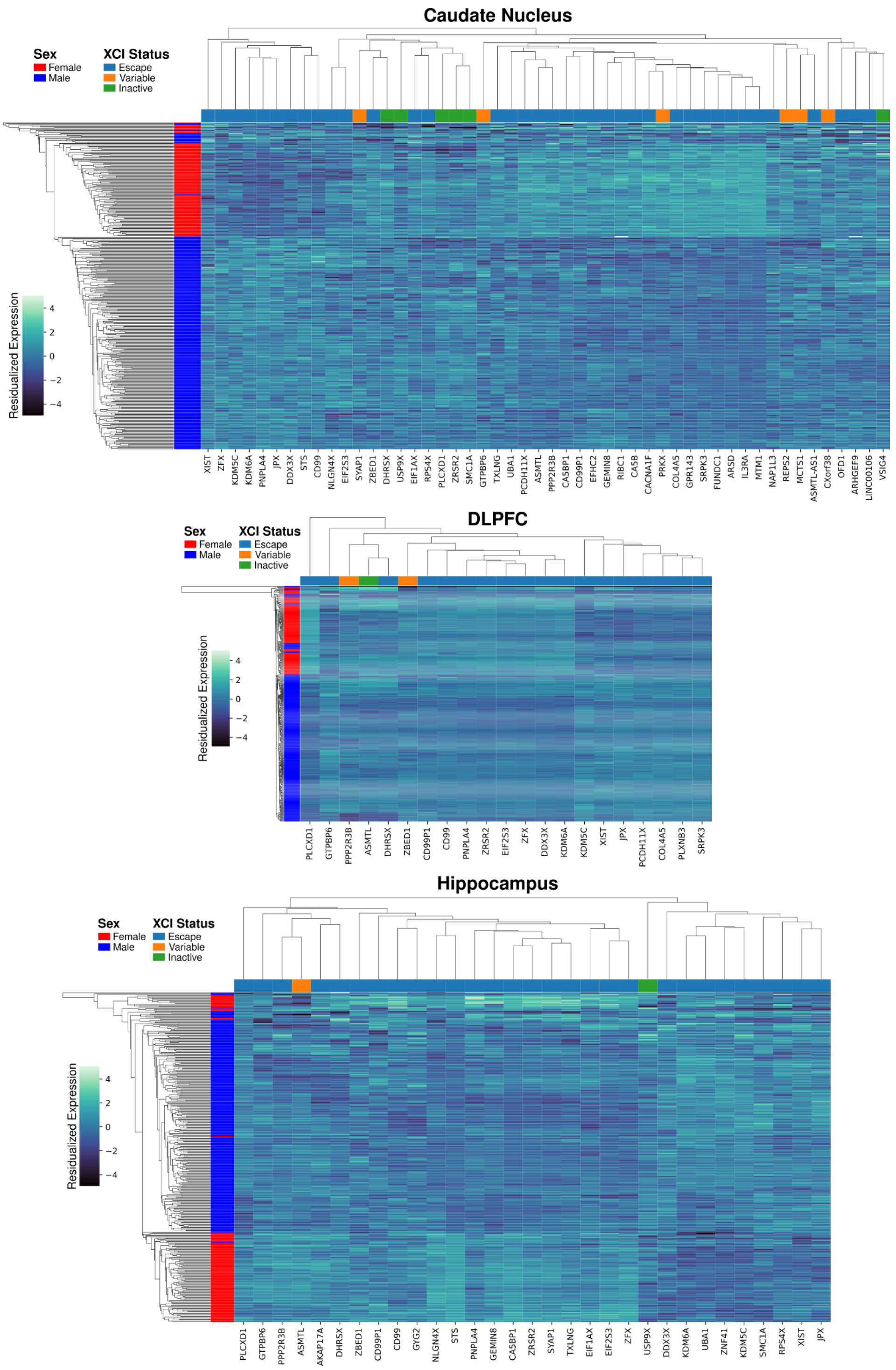

Fig. S17. Majority of XCI (X-chromosome inactivation) escaping genes are upregulated in female individuals across the brain. Heatmap plots of residualized expression for XCI differentially expressed genes for the caudate nucleus (n=393; 121 female and 272 male), dorsolateral prefrontal cortex (DLPFC; n=359; 114 female and 245 male), and hippocampus (n=375; 121 female and 254 male). Heatmap rows are annotated for sex (female: red, male: blue) and columns are annotated for XCI status (escape: blue, variable: orange, inactive: green).

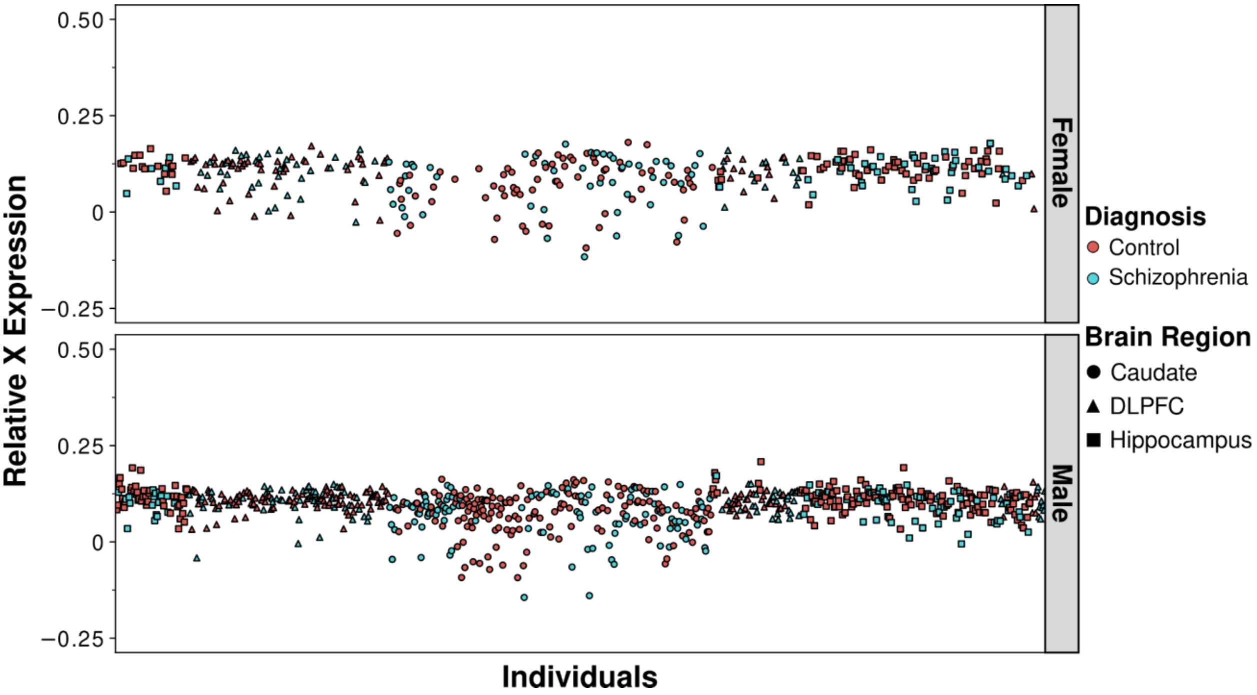

Fig. S18. Dosage is properly compensated in the caudate nucleus, DLPFC, and hippocampus. Scatterplot of relative X expression (RXE) across brain regions separated by sex. Control (red), Schizophrenia (blue), caudate nucleus (circle; n=393 [121 female and 272 male]), DLPFC (triangle; n=359 [114 female and 245 male]), and hippocampus (square; n=375 [121 female and 254 male]).

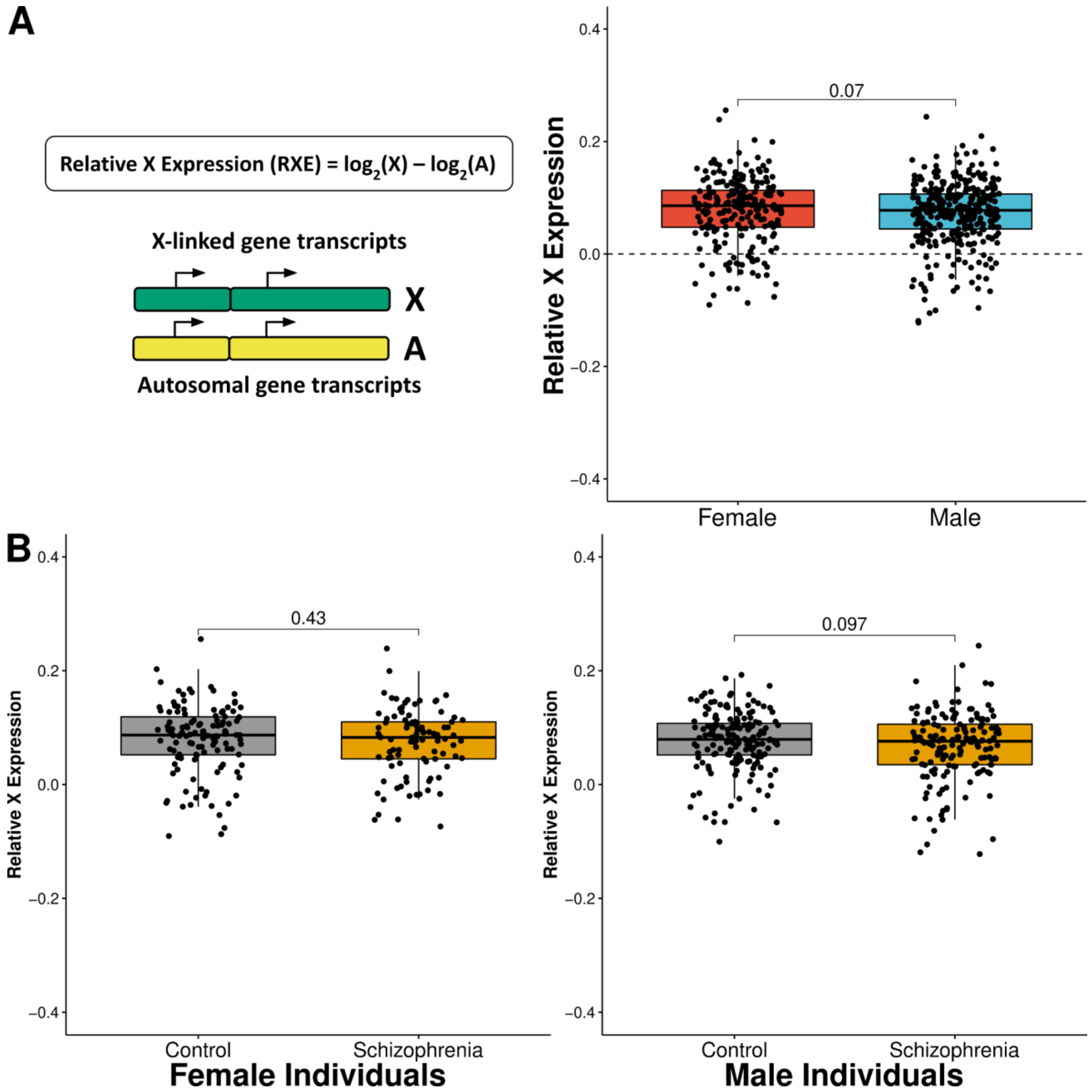

Fig. S19. Replication of relative X expression (RXE) sex differences within the DLPFC of CommonMind Consortium (CMC). A. Schematic of RXE (left) and box plots showing RXE comparison between female (red; n=315) and male (blue; n=543) individuals for the CMC DLPFC (right). **B.** Box plots showing RXE comparison between neurotypical controls (gray; n=510) and schizophrenia (gold; n=348) individuals for female (left [189 control and 126 schizophrenia]) and male (right [321 control and 222 schizophrenia]) individuals in the CMC DLPFC. Box plots show the median and first and third quartiles, and whiskers extend to 1.5× the interquartile range. Annotations are p-values from Mann-Whitney U two-tailed test.

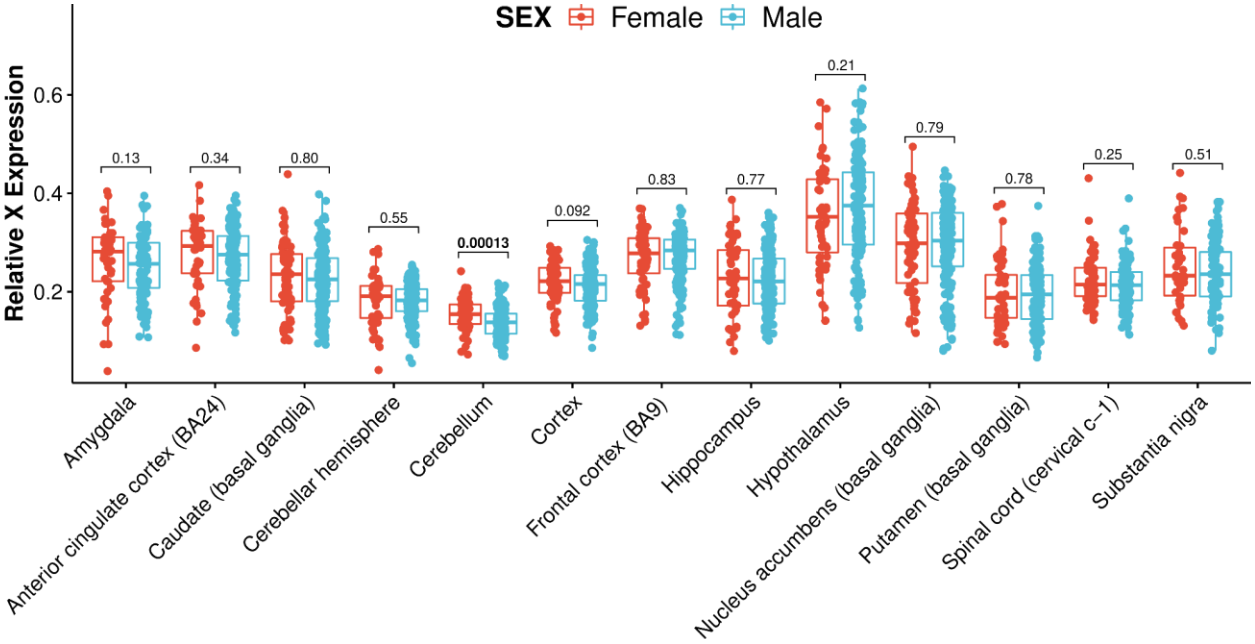

Fig. S20. Replication of relative X expression (RXE) sex differences within the 13 brain regions of GTEx shows RXE variation within GTEx brain regions. Box plots showing RXE comparison between female (red) and male (blue) individuals for the 13 GTEx brain regions. Box plots show the median and first and third quartiles, and whiskers extend to 1.5× the interquartile range. Annotations of p-values are derived from Mann-Whitney U two-tailed test. Significant differences are bolded.

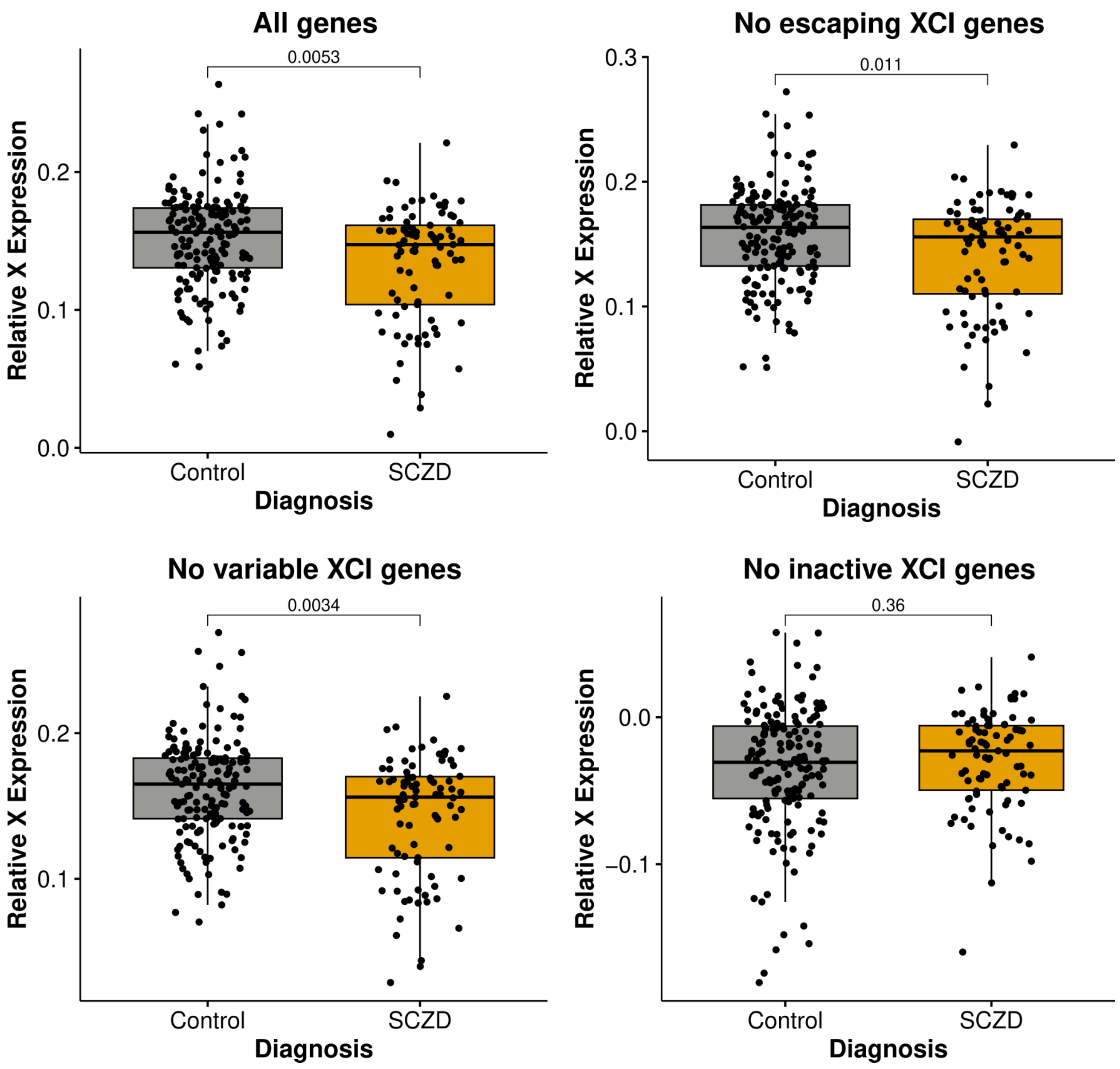

Fig. S21. Significant decrease in relative X expression (RXE) in the hippocampus of male patients with schizophrenia driven by a reduction of inactive X-chromosome inactivation (XCI) genes. Box plots of RXE expression between neurotypical controls (gray; n=188) and schizophrenia (gold; n=88) individuals in the hippocampus of male individuals (n=276). RXE expression calculated using all genes, no escaping XCI genes, no variable XCI genes, and no inactive XCI genes. Box plots show the median and first and third quartiles, and whiskers extend to 1.5× the interquartile range. Annotations are p-values from Mann-Whitney U two-tailed test.

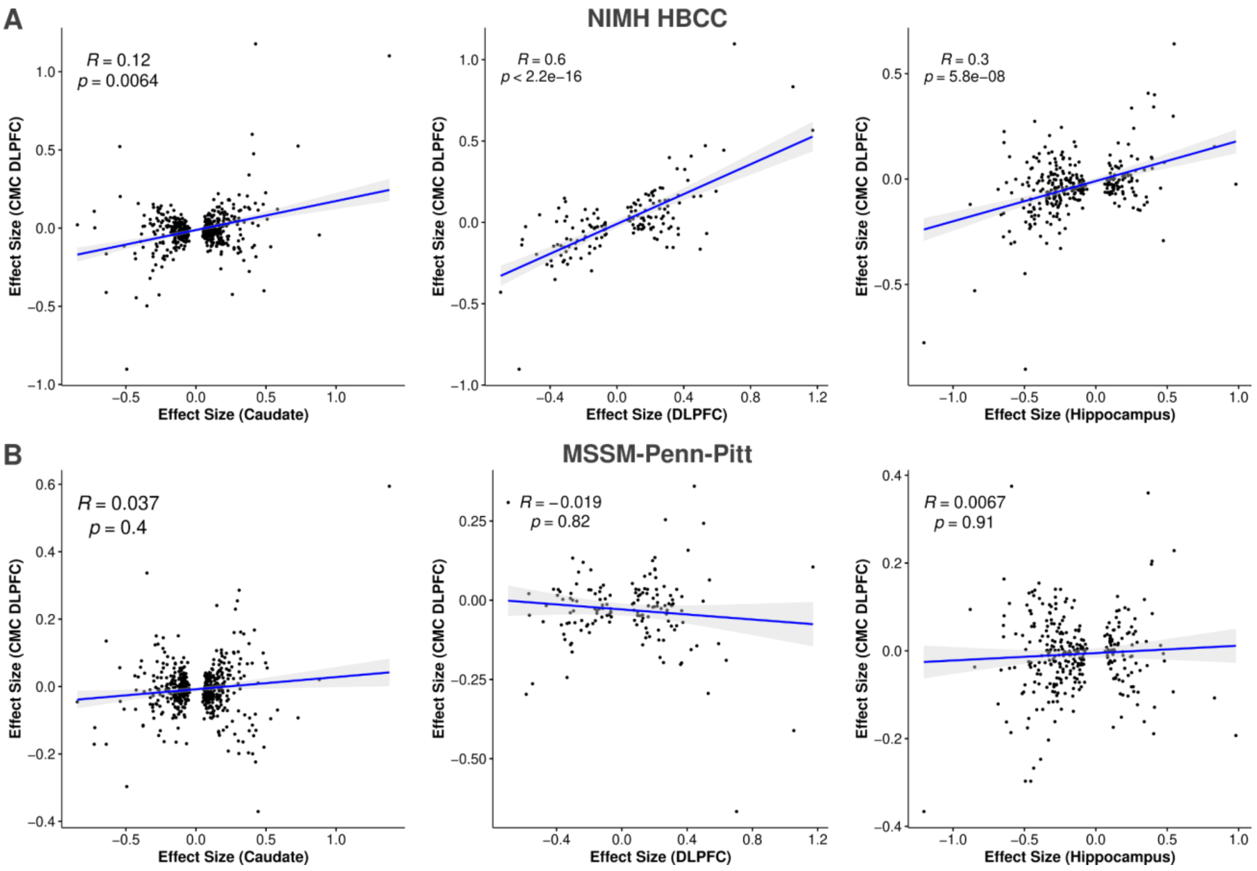

Fig. S22. Limited replication of nominally significant, sex-interacting, schizophrenia-associated DEGs (differentially expressed genes) shared with CommonMind Consortium (CMC) DLPFC (NIMH HBCC cohort). Scatterplot comparing effect size (logFC) between BrainSeq Consortium brain regions (caudate nucleus, DLPFC, and hippocampus) and CMC DLPFC cohort **A.** NIMH HBCC and **B.** MSSM-Penn-Pitt. A fitted trend line is presented in blue as the mean values +/- standard deviation. The standard deviation is shaded in light gray.

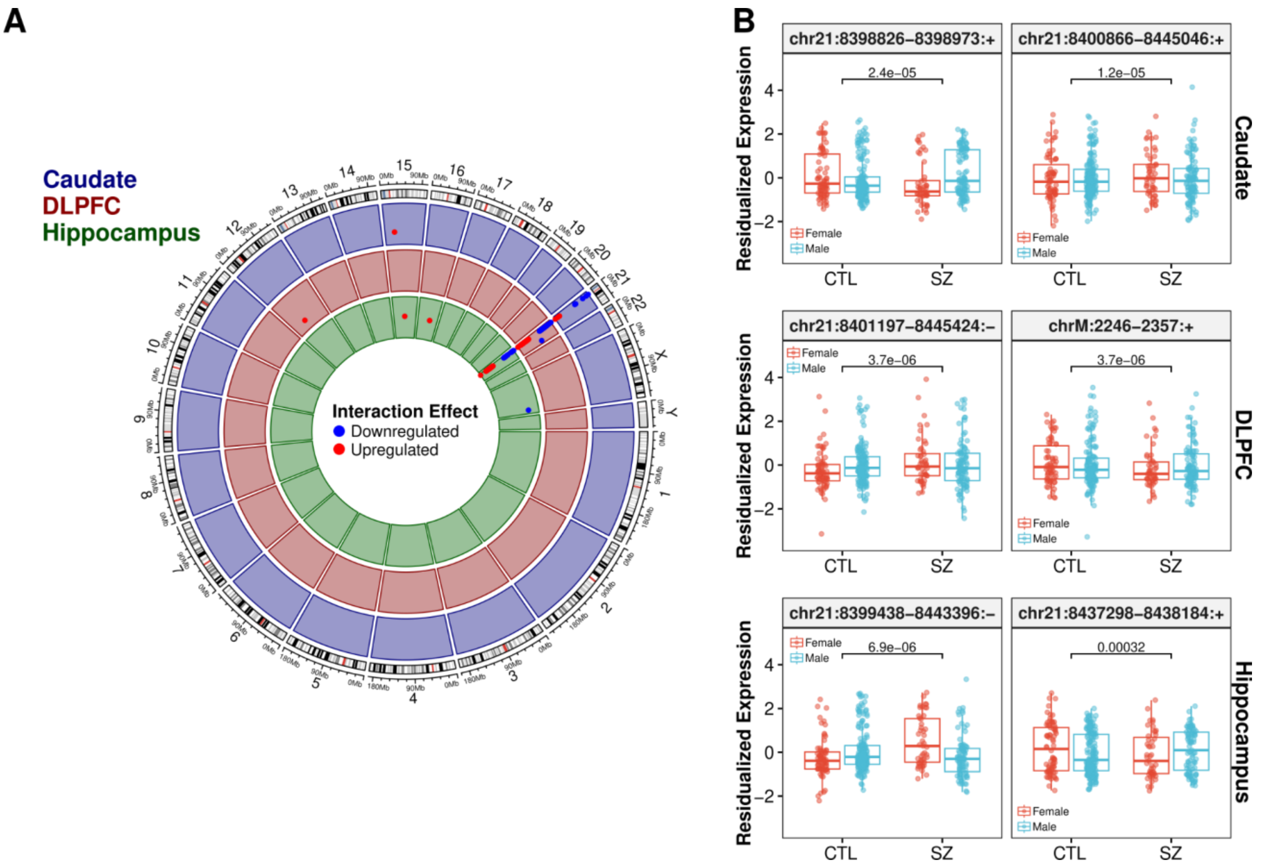

Fig. S23. Differentially expressed junctions demonstrating sex and diagnosis interaction in the caudate nucleus. A. Circos plot showing significant DE junctions for the caudate nucleus (blue; n=393), DLPFC (dorsolateral prefrontal cortex; red; n=359), and hippocampus (green; n=375) across all chromosomes. **B.** Box plots of the most significant upregulated and downregulated DE junction by brain region. Neurotypical controls (CTL), schizophrenia (SZ), female (red), and male (blue). Adjusted p-value from the DE-interacting model is annotated on the box plots. Box plots show the median and first and third quartiles, and whiskers extend to 1.5× the interquartile range.

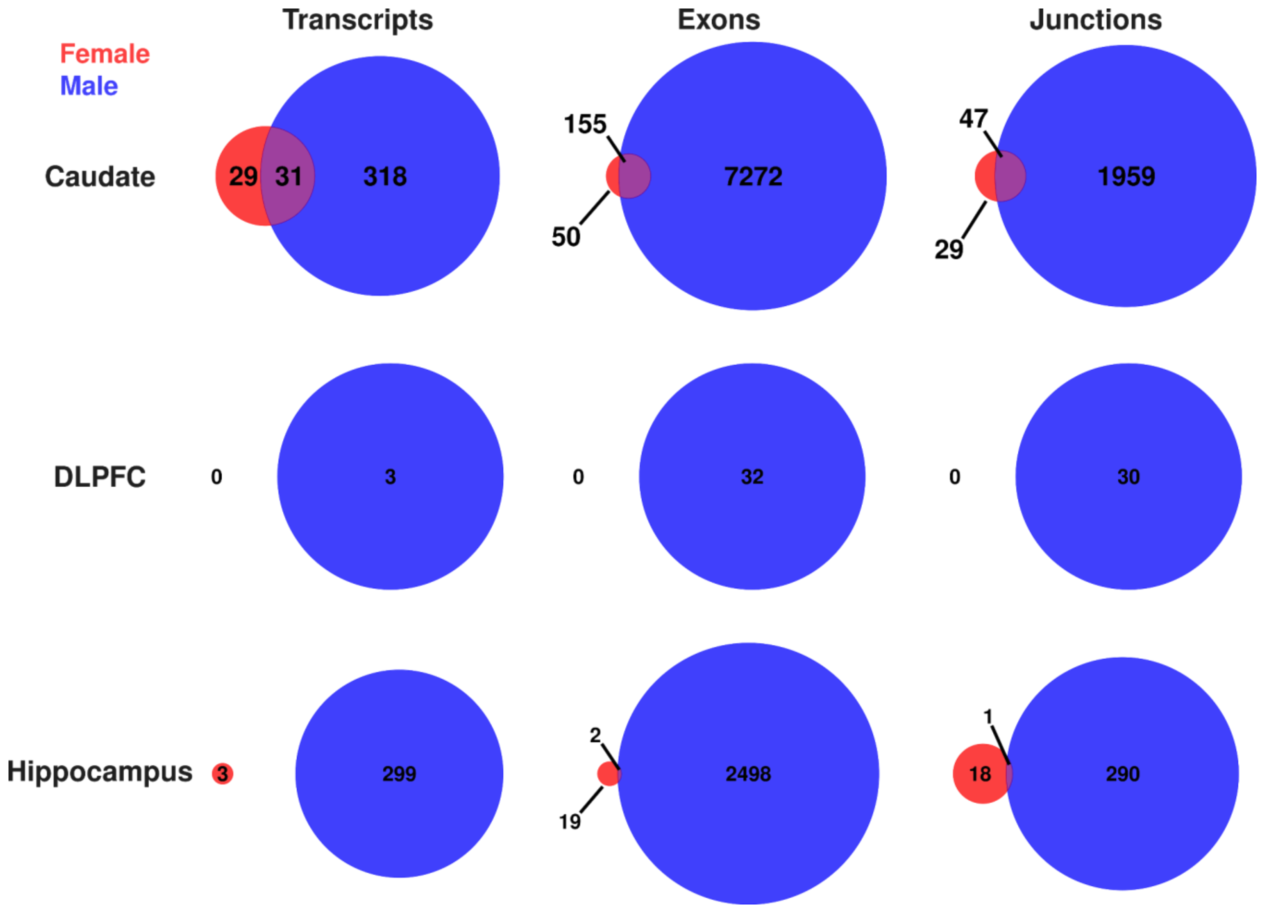

Fig. S24. Little identification of female-specific schizophrenia differential features (transcripts, exons, and junctions) across the caudate nucleus, DLPFC, and hippocampus. Venn diagram showing little to no overlap for DLPFC and hippocampus between female (red) and male (blue) schizophrenia differentially expressed transcripts, exons, and exon-exon junctions.

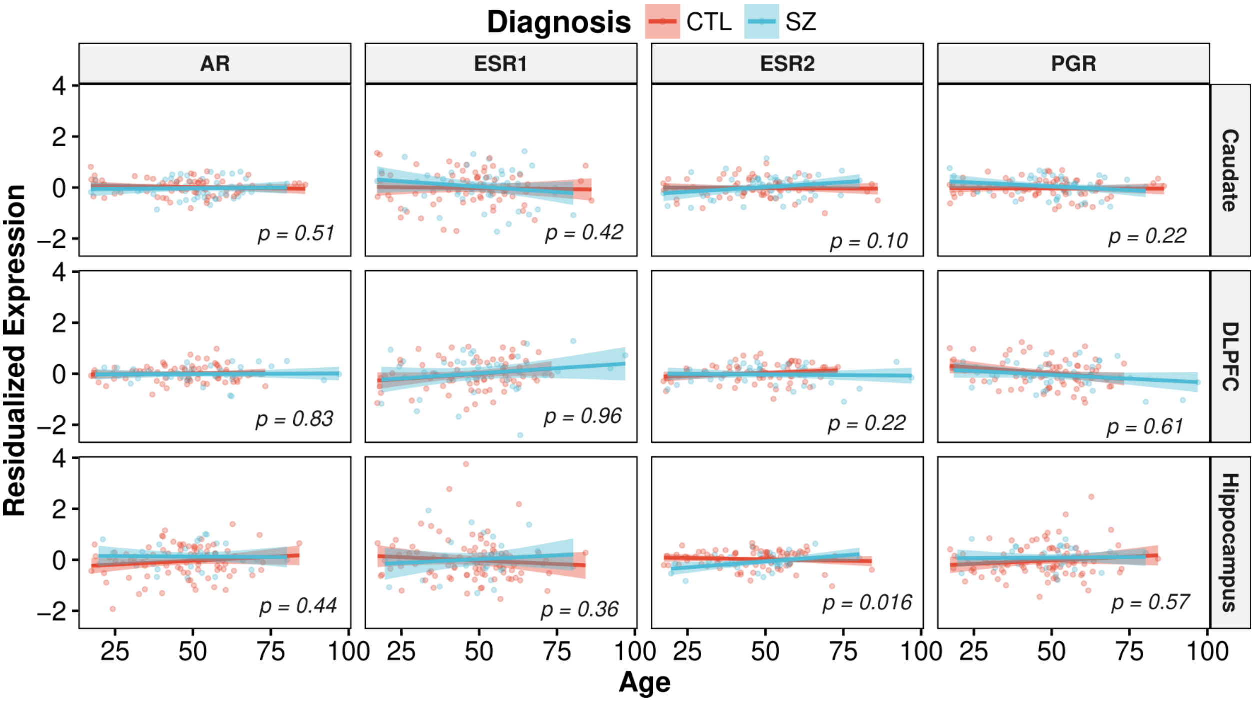

Fig. S25. Nominal significant interaction of age and diagnosis in the hippocampus for *ESR2* for female individuals. Scatterplot of residualized expression showing correlation with age as a function of diagnosis status (control [CTL] in red and schizophrenia [SZ] in blue). A fitted trend line is presented as the mean values +/- standard deviation separated by diagnosis status (control in red and schizophrenia in blue). The standard deviation is shaded in by diagnosis status (control in red and schizophrenia in blue).

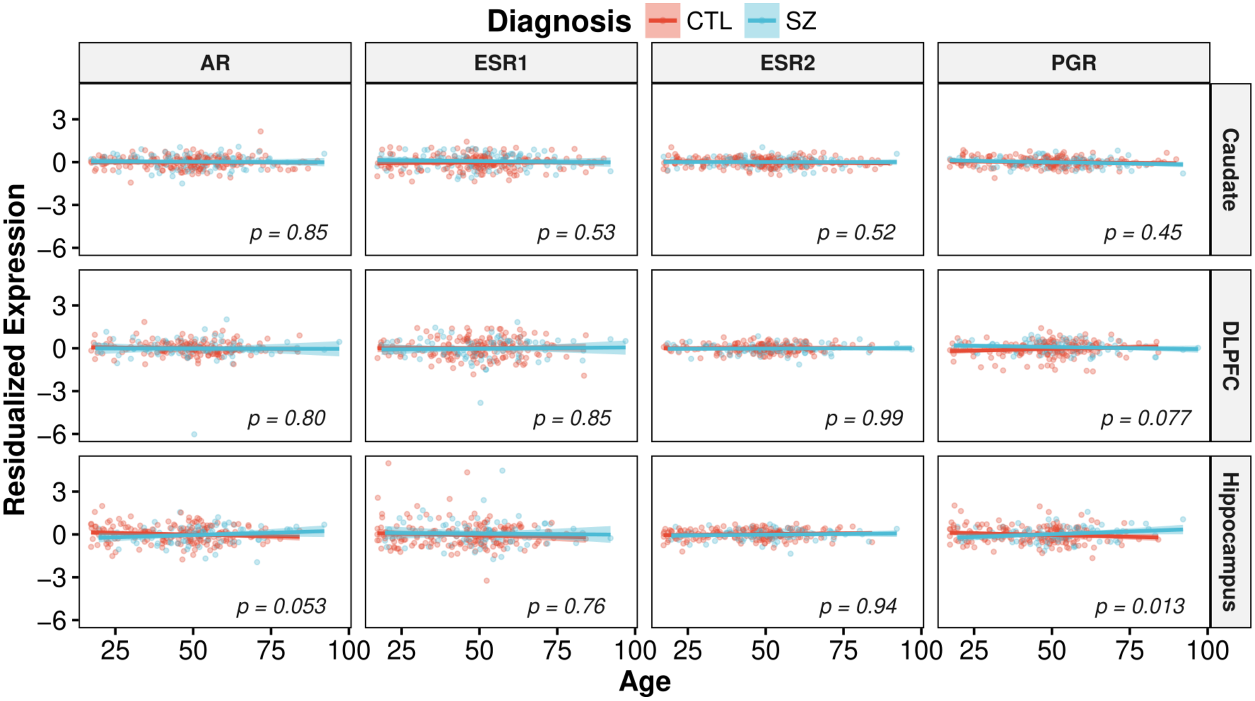

Fig. S26. No significant interaction of age and diagnosis in the hippocampus for *ESR2* for male individuals. Scatterplot of residualized expression showing no significant correlation with age as a function of diagnosis status (control [CTL] in red and schizophrenia [SZ] in blue). A fitted trend line is presented as the mean values +/- standard deviation separated by diagnosis status (control in red and schizophrenia in blue). The standard deviation is shaded in by diagnosis status (control in red and schizophrenia in blue).

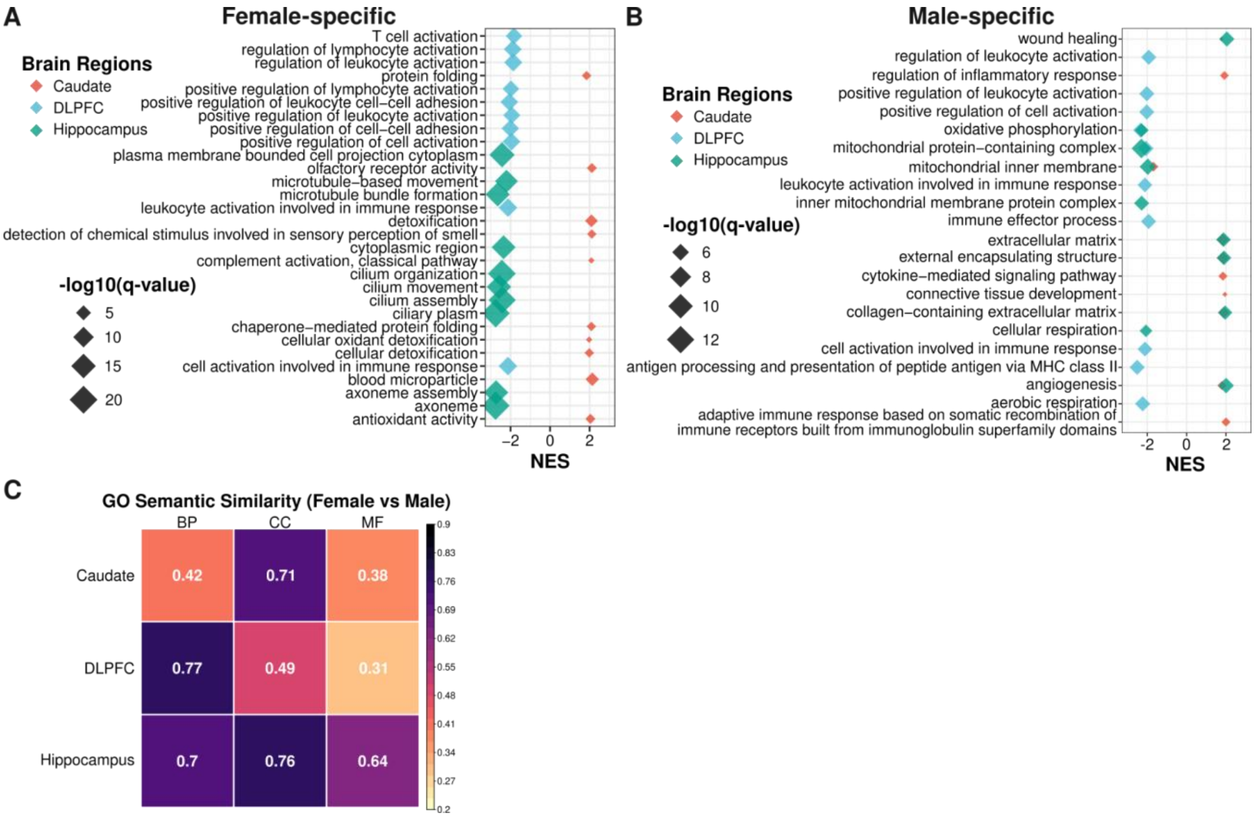

Fig. S27. Sex-specific schizophrenia DEGs show large overlap and enrichment for immune-related pathways. Plot of the most significant by p-value gene terms from the gene term enrichment analysis. NES > 0: upregulated in individuals with schizophrenia and NES < 0: downregulated in individuals with schizophrenia.

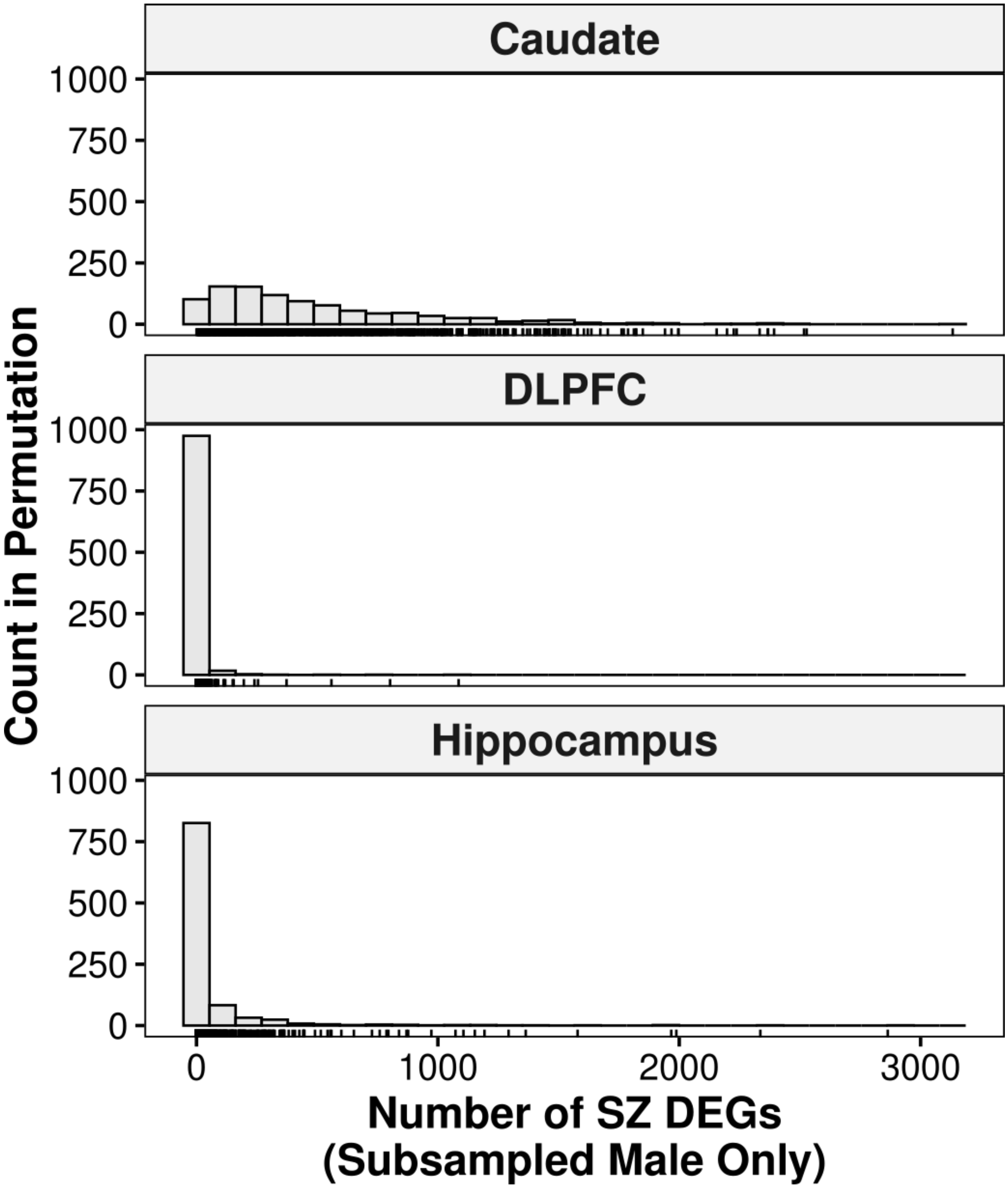

Fig. S28. Reduction of the detected schizophrenia differentially expressed genes (DEGs) in male-only analysis at smaller sample sizes. Histogram of permutation analysis of male-only differential expression analysis at female sample size levels (n=121, 114, and 121 for the caudate nucleus, DLPFC, and hippocampus, respectively) shows a reduction of detected schizophrenia DEGs similar to female-only schizophrenia analysis.

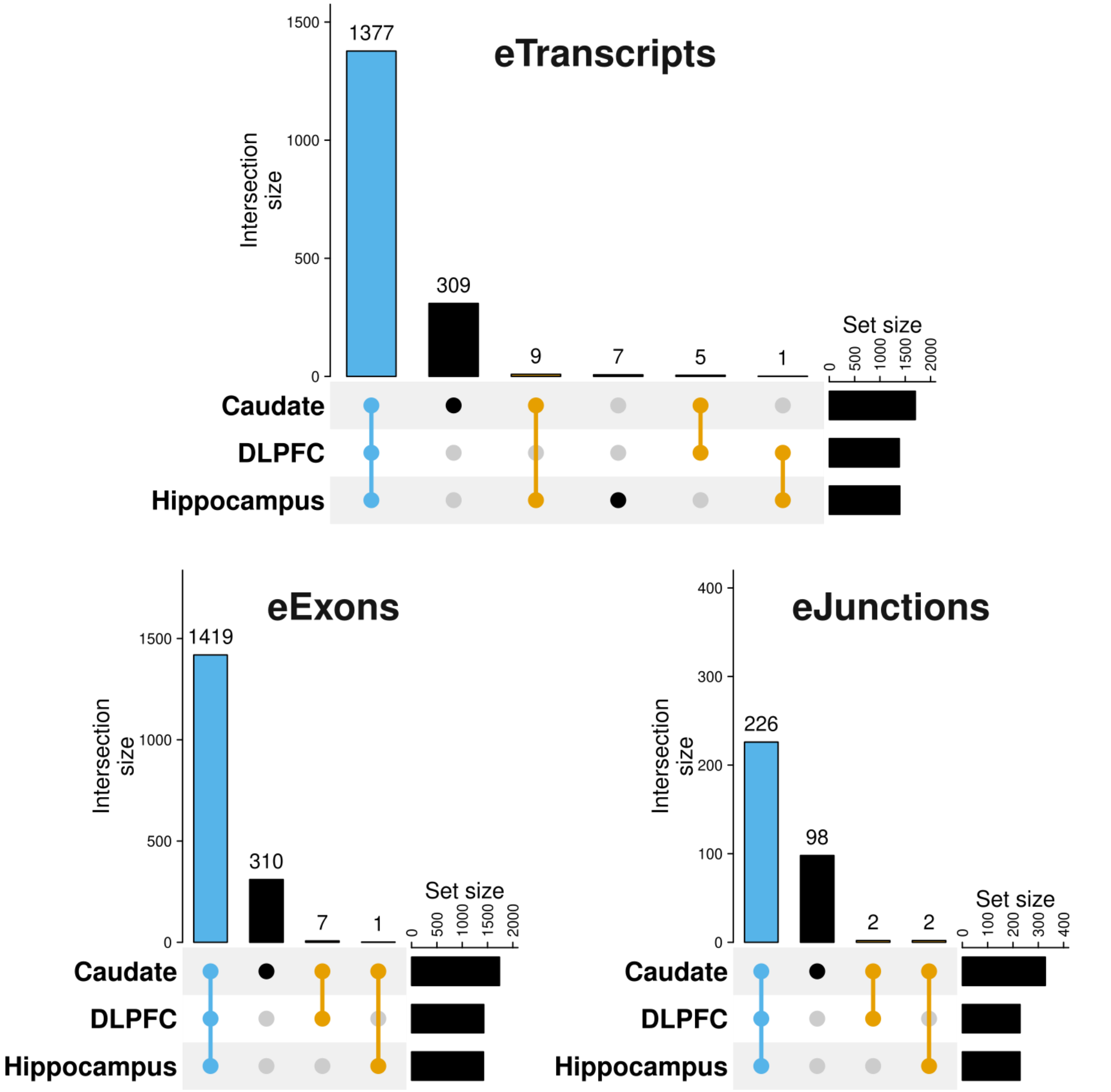

Fig. S29. si-eQTLs are shared across brain regions. UpSet plots showing the majority of si-eQTL are shared across features for eTranscripts (si-eQTL associated with unique transcripts), eExons (si-eQTL associated with unique exons), and eJunctions (si-eQTL associated with unique junctions). Blue is shared across three brain regions; orange, shared between two brain regions; and black, unique to a specific brain region.

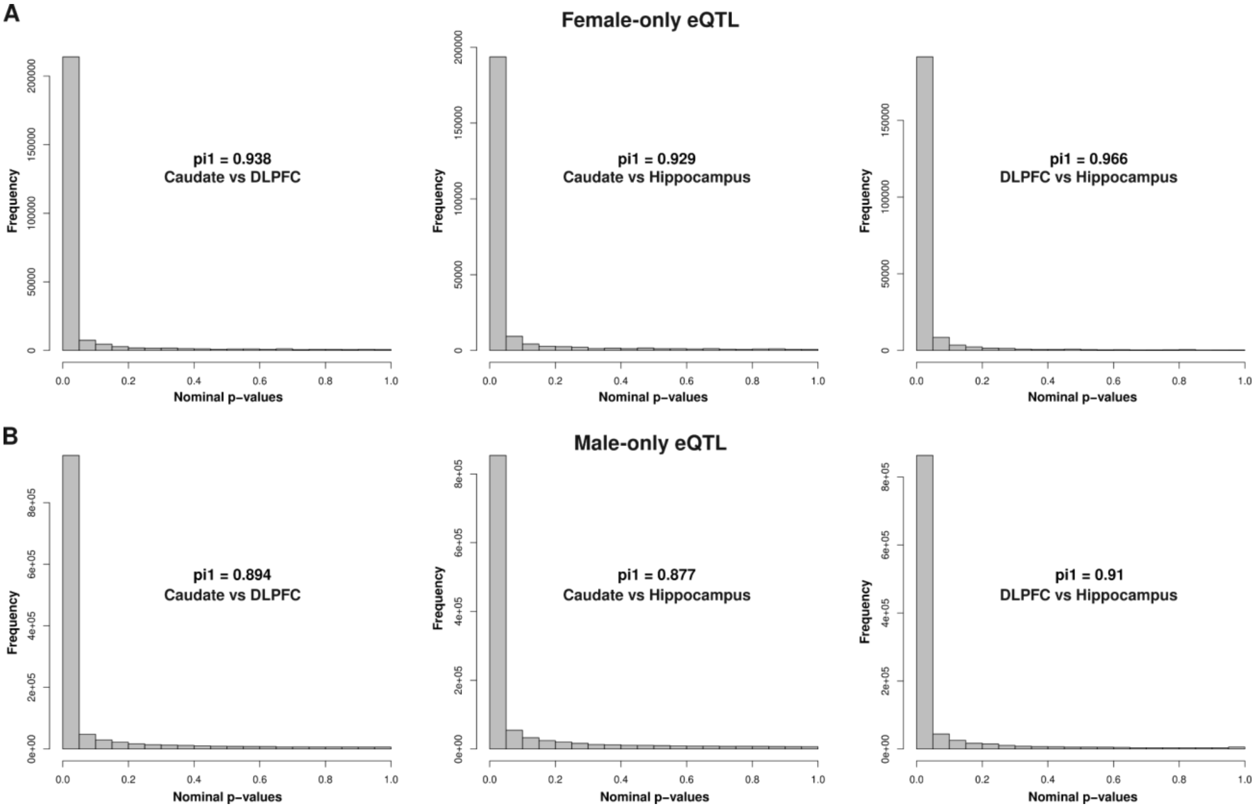

Fig. S30. High level of replication of si-eQTL across brain regions. Histogram of significant si-eQTL (lfsr < 0.05) from nominal p-values generated from **A.** female-only and **B.** male-only eQTL analyses. $\pi 1$ (pi1) statistic annotated on histograms.

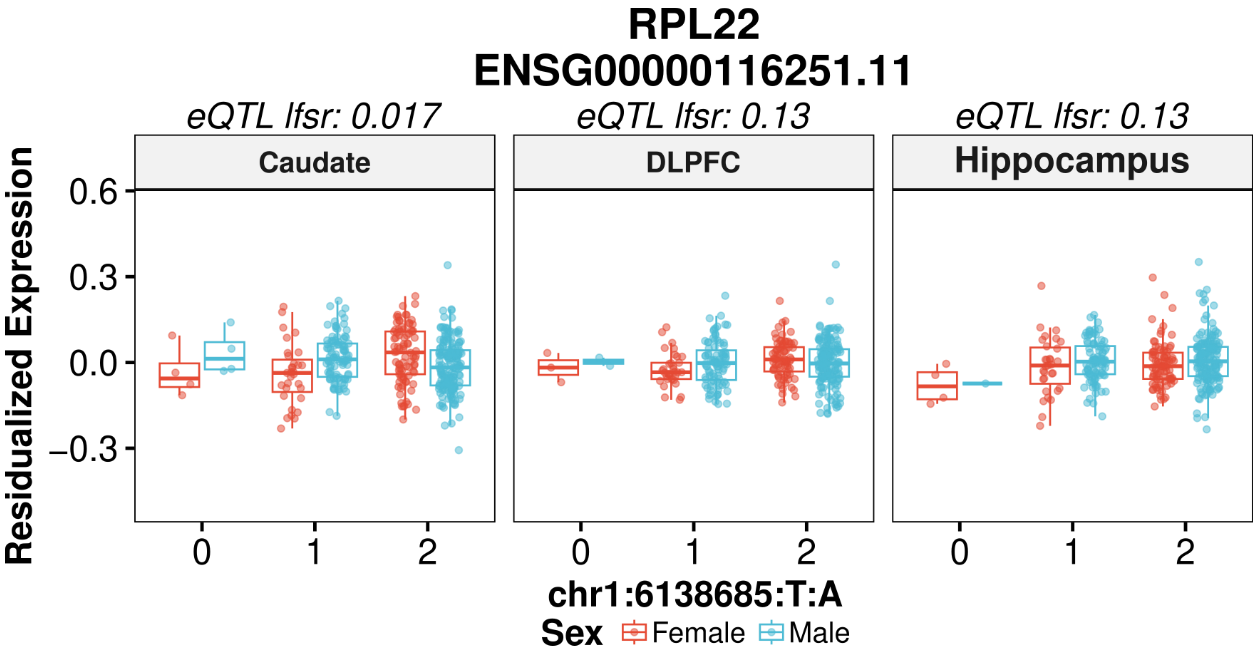

Fig. S31. Example box plot of caudate nucleus-specific si-eQTL. Example of caudate nucleus-specific si-eQTL (lfsr < 0.05). All other si-eQTL are shared with the caudate nucleus. Box plots show the median and first and third quartiles, and whiskers extend to 1.5× the interquartile range.

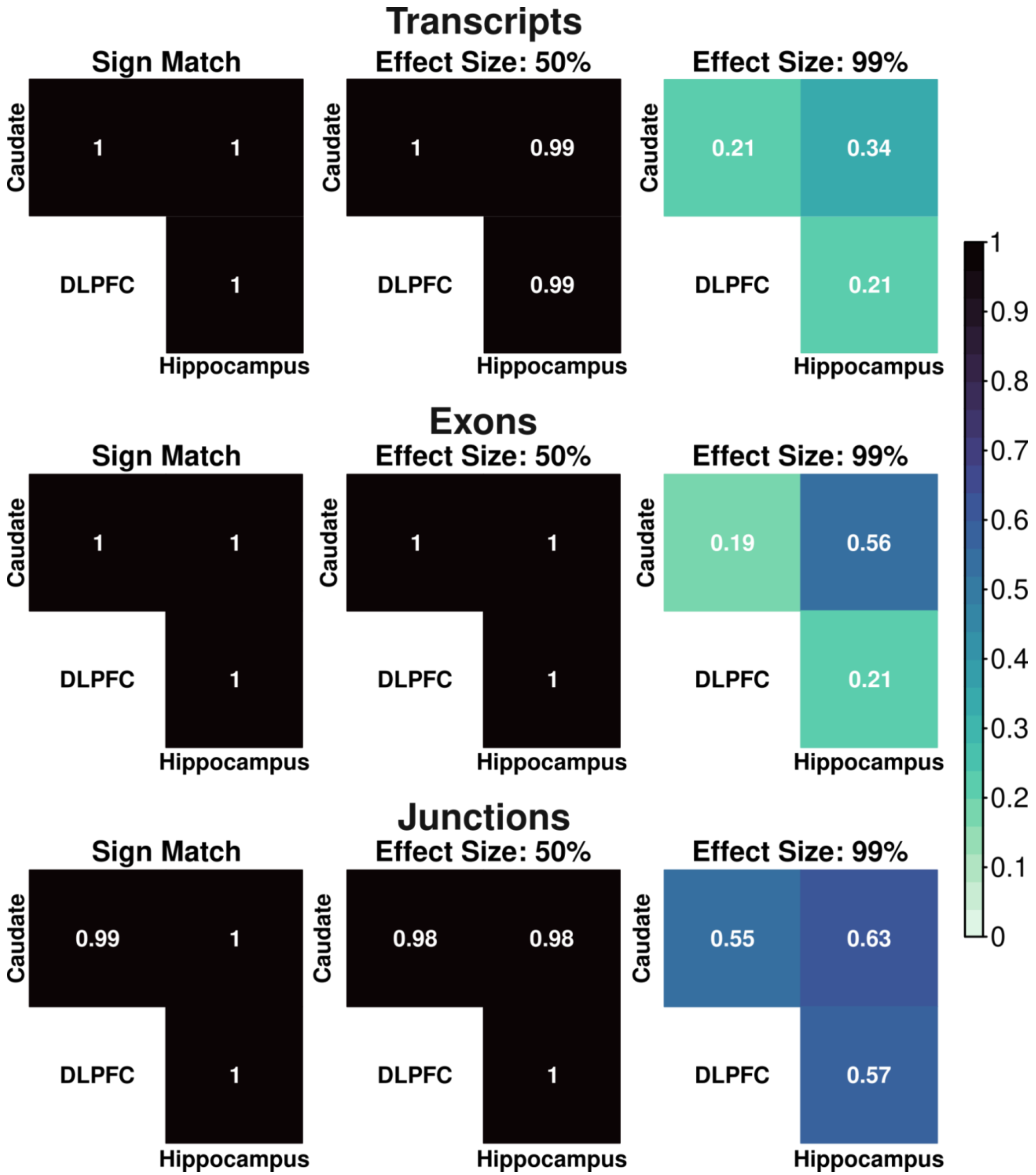

Fig. S32. si-eQTL SNP-gene pairs shared across brain regions demonstrate concordant directionality. Heatmap of proportion of feature level (transcripts, exons, and exon-exon junctions) si-eQTL sharing with the sign of si-eQTL matching (left), the same sign of si-eQTL and within a factor of 0.5 effect size (middle), and the same sign of si-eQTL and within a factor 0.99 effect size (right) using mashr[84]. A factor of one is a perfect effect size match.

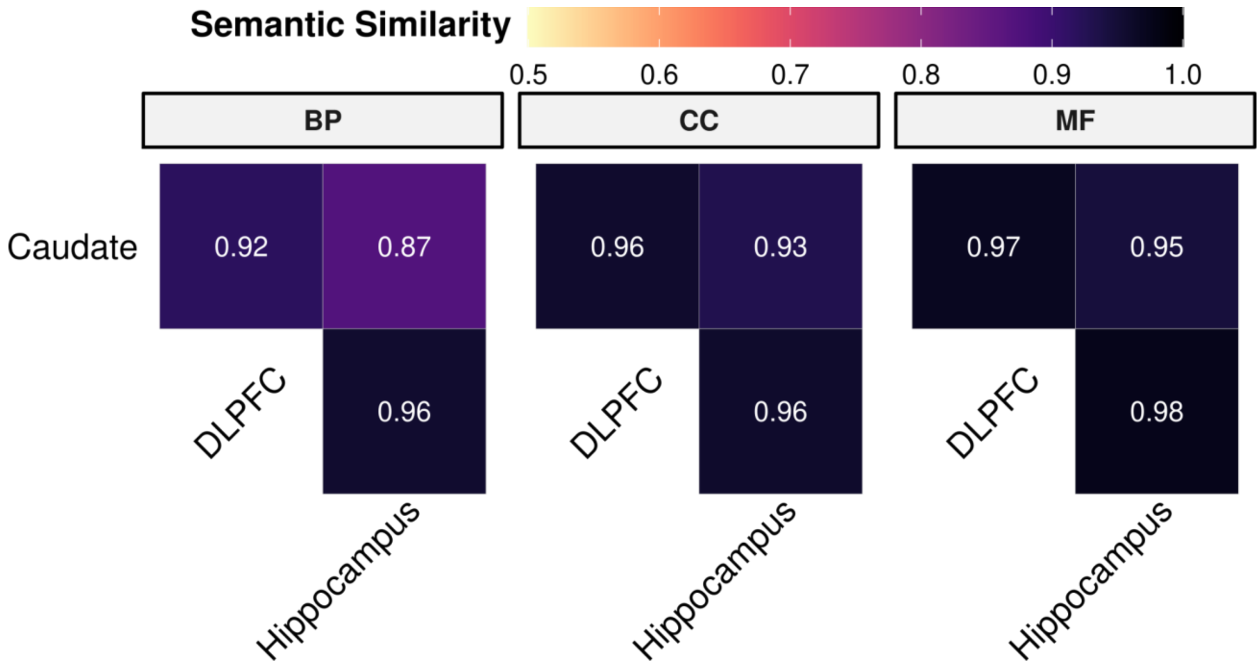

Fig. S33. Sex-interacting eGenes share functional semantic similarity across brain regions. Heatmap of significant term enrichment for the Gene Ontology database (BP: Biological Process, CC: Cellular Component, and MF: Molecular Function) across brain regions.

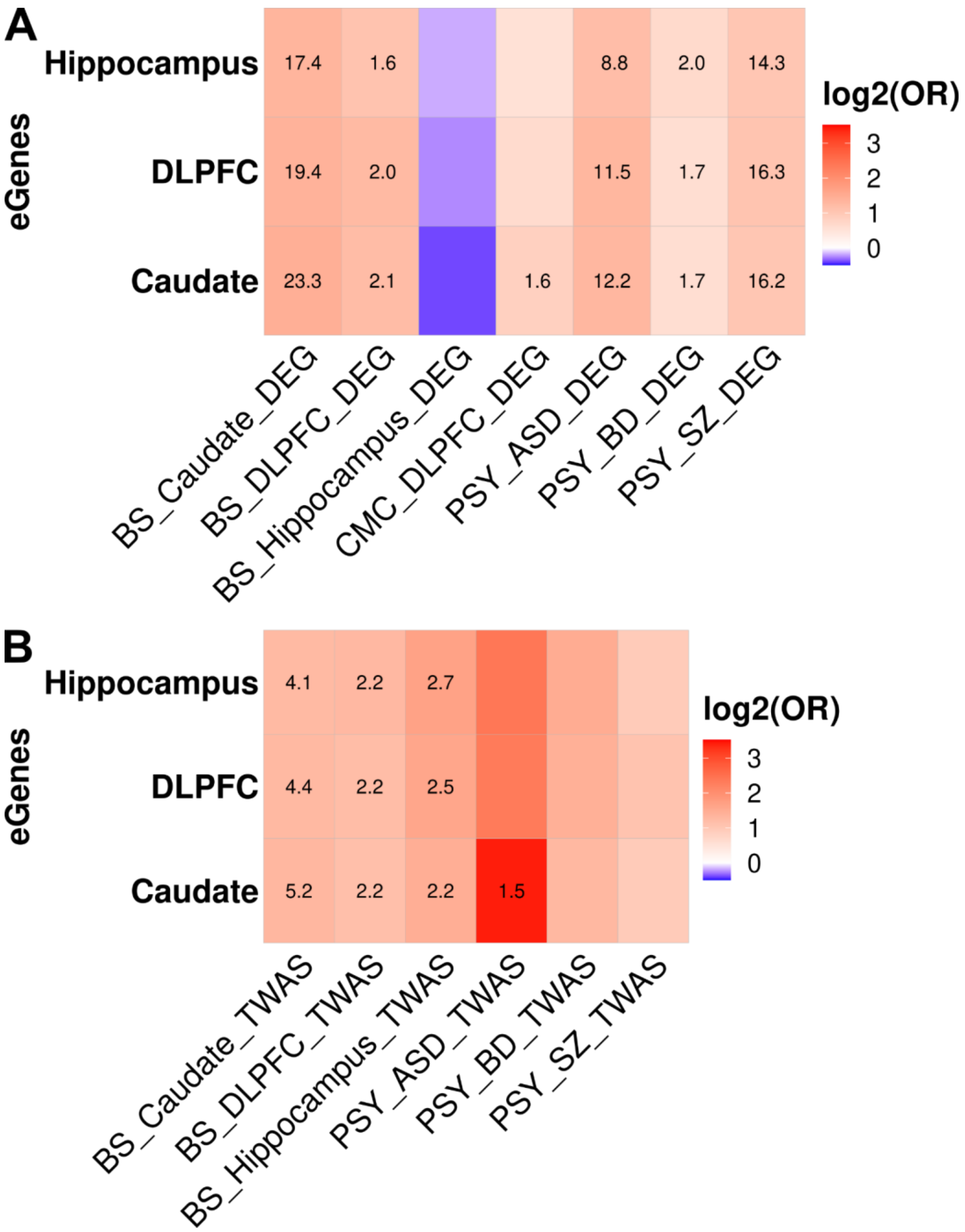

Fig. S34. Sex-interacting eGenes are enriched for neuropsychiatric disorders. Heatmap of enrichment (red) / depletion (blue) for sex-interacting eGenes with **A.** differential expression genes (DEG) or **B.** transcriptome-wide association studies (TWAS) for neuropsychiatric disorders for the caudate nucleus, DLPFC, and hippocampus. Significant enrichments (Fisher's exact test, FDR corrected p-values, -log10 transformed) annotated within tiles. BS: BrainSeq Consortium, CMC: CommonMind Consortium, PSY: psychENCODE. For BrainSeq Consortium and CMC, DEG and TWAS are for schizophrenia (SZ). ASD: Autism spectrum disorder, BD: Bipolar disorder. eGenes: si-eQTL associated with unique genes. psychENCODE results are for DLPFC.

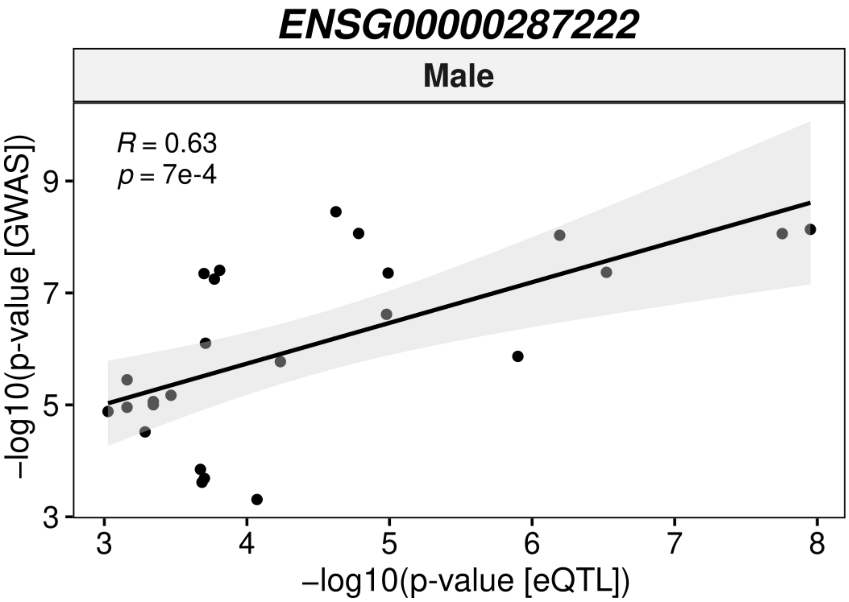

Fig. S35. Colocalization of si-eQTL and schizophrenia risk in the caudate nucleus. P-P plot of the significant schizophrenia GWAS associations (RCP > 0.5). Gene name annotated on top of scatterplot. A fitted trend line is presented in black as the mean values +/- standard deviation. The standard deviation is shaded in light gray.

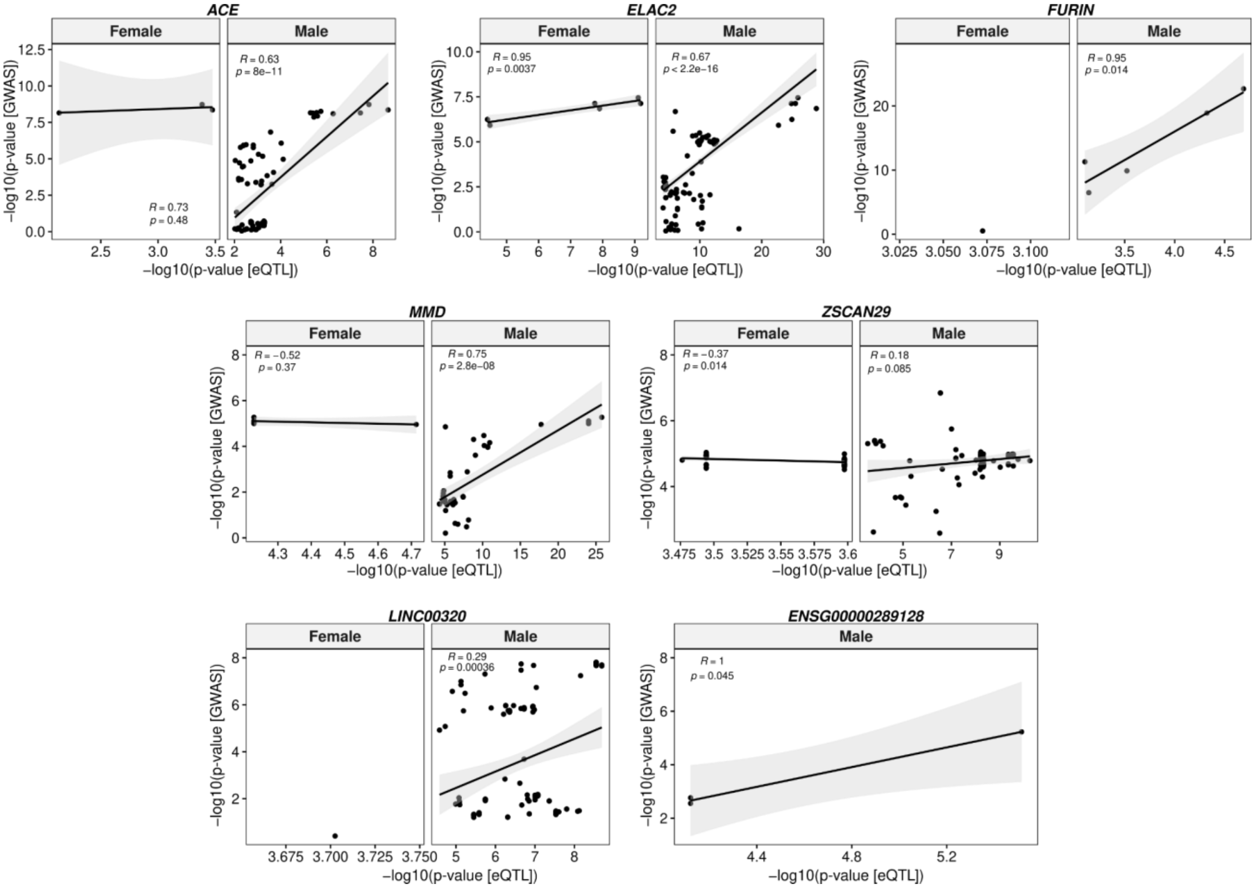

Fig. S36. Colocalization of si-eQTL and schizophrenia risk in the DLPFC. P-P plots of the significant schizophrenia GWAS associations (RCP > 0.5). Gene names annotated on top of each scatterplot. A fitted trend line is presented in black as the mean values +/- standard deviation. The standard deviation is shaded in light gray.

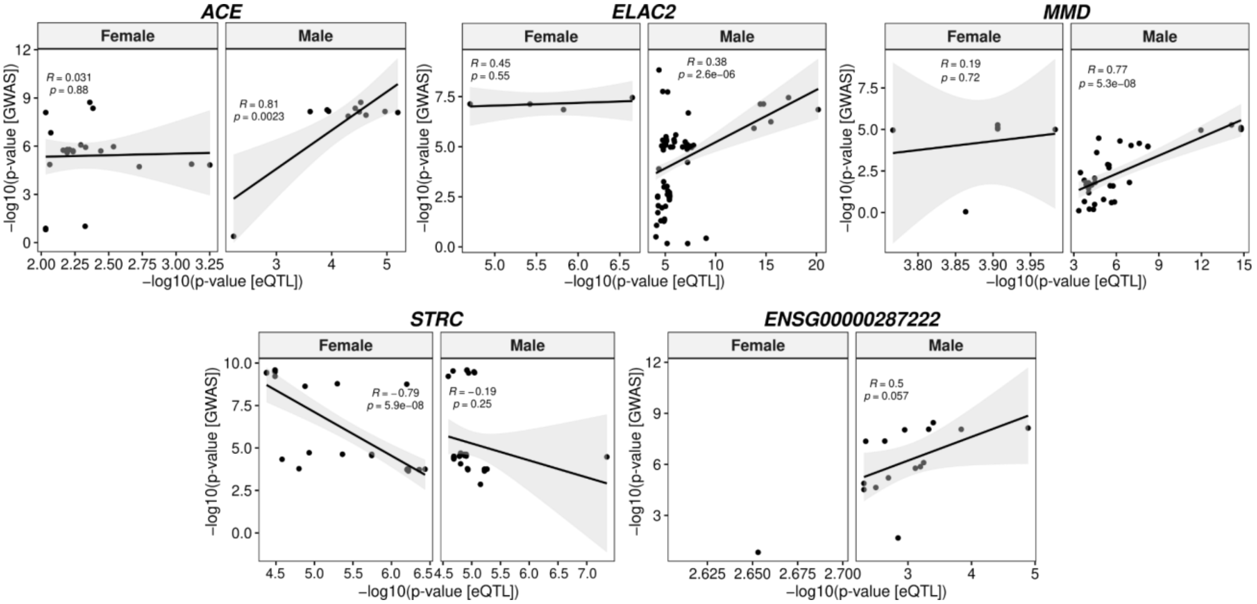

Fig. S37. Colocalization of si-eQTL and schizophrenia risk in the hippocampus. P-P plots of the significant schizophrenia GWAS associations (RCP > 0.5). Gene names annotated on top of each scatterplot. A fitted trend line is presented in black as the mean values +/- standard deviation. The standard deviation is shaded in light gray.

Tables

Table S1. Summary of GWAS trait download information including website and download filename.

GWAS Trait	Website	Filename
Schizophrenia[11]	https://www.med.unc.edu/pgc/download-results/	PGC3_SCZ_wave3.european.autosome.public.v3.vcf.tsv.gz
Depression[99]	https://datashare.ed.ac.uk/handle/10283/3203	Summary statistics of 10,000 variants from a meta-analysis of PGC, UK Biobank and 23andMe (444.2Kb)
Bipolar Disorder[100]	https://www.med.unc.edu/pgc/download-results/	daner_PGC_BIP32b_mds7a_0416a.gz
Autism[101]	https://www.med.unc.edu/pgc/download-results/	iPSYCH-PGC_ASD_Nov2017.gz
ADHD[102]	https://www.med.unc.edu/pgc/download-results/	daner_adhd_meta_filtered_NA_iPSYCH23_PGC11_sigPCs_woSEX_2ell6sd_EUR_Neff_70.meta.gz
Anorexia[103]	https://www.med.unc.edu/pgc/download-results/	pgcAN2.2019-07.vcf.tsv.gz
BMI[104]	https://portals.broadinstitute.org/collaboration/giant/index.php/GIANT_consortium_data_files	Meta-analysis_Locke_et_al+UKBiobank_2018.txt.gz
Height[26]	https://cnsgenomics.com/data/giant_2022/	https://cnsgenomics.com/data/giant_2022/GIANT_HEIGHT_YENGO_2022_GWAS_SUMMARY_STATS_EUR.gz
Basophil count[105]	https://ftp.sanger.ac.uk/project/humgen/summary_statistics/human/2017-12-12/	baso_N171846_narrow_form.tsv.gz
Neutrophil count[105]	https://ftp.sanger.ac.uk/project/humgen/summary_statistics/human/2017-12-12/	neut_N170702_narrow_form.tsv.gz
Eosinophil count[105]	https://ftp.sanger.ac.uk/project/humgen/summary_statistics/human/2017-12-12/	eo_N172275_narrow_form.tsv.gz
Monocyte count[105]	https://ftp.sanger.ac.uk/project/humgen/summary_statistics/human/2017-12-12/	mono_N170721_narrow_form.tsv.gz

Table S2. Summary of differential expression results (adjusted p-value < 0.05) by feature (genes, transcripts, exons, and exon-exon junctions) for sex differences in the caudate nucleus, DLPFC, and hippocampus. Number of differentially expressed features is separated by chromosome location. The number of unique genes associated with transcript, exon, or exon-exon junction in parenthesis. *Novel junctions not annotated to unique gene ID.

Brain Region	Chr Location	Gene	Transcript (Geneid)	Exon (Geneid)	Junction (Geneid)
Caudate nucleus	Allosomal	112	333 (100)	1523 (111)	622 (11)*
	Autosomal	576	252 (232)	1291 (419)	399 (5)*
	Mitochondria	1	2 (2)	0 (0)	3 (1)*
DLPFC	Allosomal	70	298 (97)	725 (69)	609 (10)*
	Autosomal	186	307 (291)	903 (310)	1421 (14)*
	Mitochondria	0	0 (0)	0 (0)	0 (0)*
Hippocampus	Allosomal	79	290 (88)	1361 (101)	516 (9)*
	Autosomal	68	144 (133)	669 (244)	370 (4)*
	Mitochondria	0	0 (0)	0 (0)	1 (1)*

Table S3. **Summary of random forest classification with dynamic recursive feature elimination prediction for sex using autosomes accuracy and smallest number of features.** Train, test, and smallest set metrics of mean, median, and standard deviation (std) across 10 folds for each feature (gene, transcript, exon, and exon-exon junction) and brain region (caudate nucleus, DLPFC, and hippocampus).

Feature	Brain Region	N Features		Train Accuracy (%)		Test Accuracy (%)	
		Median	Mean ± Std	Median	Mean ± Std	Median	Mean ± Std
Gene	Caudate nucleus	77	349 ± 609	100	99.8 ± 0.4	100	100 ± 0
	DLPFC	32.5	50.6 ± 29.8	100	99.6 ± 0.5	100	99.2 ± 1.3
	Hippocampus	221	355 ± 538	100	99.7 ± 0.2	100	99.7 ± 0.8
Transcript	Caudate nucleus	51	51.9 ± 7.9	93.8	94.0 ± 0.8	93.7	92.9 ± 5.6
	DLPFC	84	91 ± 20.7	98.6	98.4 ± 0.4	100	98.6 ± 2.0
	Hippocampus	60.5	55.8 ± 41.4	97.3	97.4 ± 0.3	97.4	97.9 ± 2.1
Exon	Caudate nucleus	219	196.6 ± 135	100	99.9 ± 0.1	100	99.7 ± 0.8
	DLPFC	56.5	75.4 ± 62.9	99.1	98.9 ± 1.1	100	98.6 ± 2.7
	Hippocampus	324.5	352 ± 324	99.7	99.7 ± 0.1	100	99.7 ± 0.8
Junction	Caudate nucleus	66.5	69.3 ± 36.5	98.7	98.6 ± 0.8	97.4	97.7 ± 1.9
	DLPFC	28	31.6 ± 16.1	99.1	99.2 ± 0.3	100	99.2 ± 1.3
	Hippocampus	48.5	42.3 ± 18.3	98.8	99.0 ± 0.4	100	99.7 ± 0.8

Table S4. Summary results of differential expression analysis (adjusted p-value < 0.05) of interaction for sex and brain region for genes, transcripts, exons, and exon-exon junctions. The number of unique genes associated with transcript, exon, or exon-exon junction in parenthesis. *Novel junctions not annotated to unique gene ID.

**Brain Regions**	**Gene**	**Transcript (Geneid)**	**Exon (Geneid)**	**Junction (Geneid)**
Caudate nucleus and DLPFC	528	728 (636)	2780 (789)	694 (3)*
Caudate nucleus and Hippocampus	71	137 (102)	256 (95)	127 (5)*
DLPFC and Hippocampus	5	25 (19)	6 (3)	80 (2)*

Table S5. Summary of π1 statistic for sex-specific schizophrenia nominally significant DEGs (p-value < 0.05) between BrainSeq (caudate nucleus, DLPFC, and hippocampus) and CMC DLPFC by cohort.

CMC DLPFC cohort	BrainSeq Region	π1 statistic
MSSM-Penn-Pitt	Caudate nucleus	0
	DLPFC	0
	Hippocampus	0
NIMH HBCC	Caudate nucleus	0.07
	DLPFC	0.51
	Hippocampus	0

Table S6. Summary of stringent sex-specific differential expression analysis (FDR < 0.05) for schizophrenia by sex and feature (gene, transcript, exon, and exon-exon junction) for the caudate nucleus, DLPFC, and hippocampus. The number of unique genes associated with transcript, exon, or exon-exon junction in parenthesis. *Novel junctions not annotated to unique gene ID.

Brain Region	Sex	Gene	Transcript (Geneid)	Exon (Geneid)	Junction (Geneid)
Caudate nucleus	Female	194	16 (16)	22 (17)	15 (1)*
	Male	1130	190 (178)	3645 (864)	1007 (3)*
DLPFC	Female	0	0 (0)	0 (0)	0 (0)*
	Male	5	1 (1)	23 (3)	17 (1)*
Hippocampus	Female	0	0 (0)	7 (7)	4 (2)*
	Male	149	105 (104)	865 (53690)	72 (3)*

Table S7. Summary of shared genes between two brain regions for stringent male-specific schizophrenia DEGs.

Brain Regions	# of Genes	Gene Names
Caudate nucleus and DLPFC	2	*EDN3* and *PLD4*
Caudate nucleus and Hippocampus	23	*MIDEAS*, *IL1R1*, *SLC11A1*, *DCP1A*, *OSMR*, *ENSG00000272601*, *TMEM52B*, *CHD2*, *ACSL5*, *MACORIS*, *GPD1*, *BCL6*, *JAK3*, *MYC*, *ZBTB16*, *TAC1*, *MMD2*, *IL18BP*, *PLRG1*, *ETV6*, *ZNF395*, and *SPAG7*
DLPFC and Hippocampus	0	Not available
