# Supplementary material for: Sex affects transcriptional associations with schizophrenia across the dorsolateral prefrontal cortex, hippocampus, and caudate nucleus
Source: Nat Commun. 2024 May 10;15:3980. doi: 10.1038/s41467-024-48048-z (PMC11087501; doi:10.1038/s41467-024-48048-z)
Supplement: Supplementary file 3 — Description of Additional Supplementary Information [file 41467_2024_48048_MOESM3_ESM.docx]

### **Description of Additional Supplementary Information**

[**Data S1**](#sdata_de)**. differential_expression_analysis_4features_sex.txt.gz**: Compressed text file of differential expression analysis for sex across the caudate nucleus, DLPFC, and hippocampus for four features (gene, transcript, exon, and exon-exon junction).

[**Data S2**](#sdata_magma)**.** **BrainSeq_MAGMA_enrichment_analysis.xlsx**: Excel file of magma enrichment results of all DEGs (i.e., sex-specific, sex interacting with brain region, and sex-specific schizophrenia) separated by direction of effect across the caudate nucleus, DLPFC, and hippocampus.

[**Data S3**](#sdata_funDE)**. BrainSeq_sex_specific_functional_enrichment_3brain_regions.xlsx**: Excel file of GO-term enrichment and GSEA for sex across the caudate nucleus, DLPFC, and hippocampus.

[**Data S4**](#sdata_wgcnaSex)**.** **BrainSeq_sex_WGCNA_results.tar.gz**: Compressed tar file containing with WGCNA results for autosomal only and all genes sex networks including eigengenes, module membership text files, GO enrichment text and excel file results, and enrichment results with sex DEG analysis for the caudate nucleus, DLPFC, and hippocampus.

[**Data S5**](#sdata_modPreSZ)**. BrainSeq_module_preservation_zsummary.xlsx**: Excel file of Z summary for module preservation across brain regions for all individuals, control only, and schizophrenia only.

[**Data S6**](#sdata_brainXsex)**. differential_expression_region_interaction_sex_4features.txt**: Text file of differential expression analysis for the interaction between a brain region and sex for the four features (gene, transcript, exon, and exon-exon junction).

[**Data S7**](#sdata_regionGSEA)**. BrainSeq_region_interaction_sex_functional_enrichment.xlsx**: Excel file of GSEA for sex and region interaction pairwise enrichment.

[**Data S8**](#sdata_xci)**. BrainSeq_male_biased_genes_XCI_status.tsv**: Text file of male-biased (upregulated in male individuals) DEGs across the caudate nucleus, DLPFC, and hippocampus annotated for XCI status.

[**Data S9.**](#sdata_jxn) **differential_expression_interaction_jxn.txt**: Text file of differentially expressed exon-exon junctions for interaction model of schizophrenia and sex across the caudate nucleus, DLPFC, and hippocampus.

[**Data S10**](#sdata_sexXsz)**. differential_expression_schizophrenia_by_sex_4features.txt.gz**: Compressed text file of differential expression analysis for schizophrenia by sex across the caudate nucleus, DLPFC, and hippocampus for four features (gene, transcript, exon, and exon-exon junction).

[**Data S11**](#sdata_funSex)**. functional_enrichment_analysis_maleSZ_3brain_regions.txt**: Functional enrichment of male-specific schizophrenia across three brain regions using a stringent gene list (all DEGs, upregulated and downregulated in schizophrenia).

[**Data S12**](#sdata_siEQTL)**. BrainSeq_sexGenotypes_4features_3regions.txt.gz**: Compressed text file of sex-interacting eQTL results across the caudate nucleus, DLPFC, and hippocampus for four features (gene, transcript, exon, and exon-exon junction) generated using mash modeling.

[**Data S13**](#sdata_funEQTL)**. BrainSeq_sex_interacting_eGene_functional_enrichment.xlsx**: Excel file of functional enrichment of sex-interacting eQTL associated with unique genes for the caudate nucleus, DLPFC, and hippocampus.

[**Data S14**](#sdata_siEQTLpublic)**.** **BrainSeq_siEQTL_public_comparison.xlsx**: Excel file of si-eQTL shared with the public datasets[^19,42^](https://sciwheel.com/work/citation?ids=8988059,9641906&pre=&pre=&suf=&suf=&sa=0,0).

[**Data S15**](#sdata_finemap)**. dapg_sex_interacting_eQTL_results.tar.gz**: si-eQTL results after fine-mapping across the three brain regions for four features (gene, transcript, exon, and exon-exon junction).

[**Data S16**](#sdata_coloc)**. BrainSeq_colocalization_3regions.xlsx**: Excel file of gene level colocalization between DAP-G fine-mapped sex-interacting eQTL and schizophrenia GWAS[^11^](https://sciwheel.com/work/citation?ids=12782344&pre=&suf=&sa=0) on the signal level and individual SNP level using fastENLOC.
