# Supplementary figures and images for: Sex affects transcriptional associations with schizophrenia across the dorsolateral prefrontal cortex, hippocampus, and caudate nucleus
Source: Nat Commun. 2024 May 10;15:3980. doi: 10.1038/s41467-024-48048-z (PMC11087501; doi:10.1038/s41467-024-48048-z)

## Sample dendrogram and trait heatmap

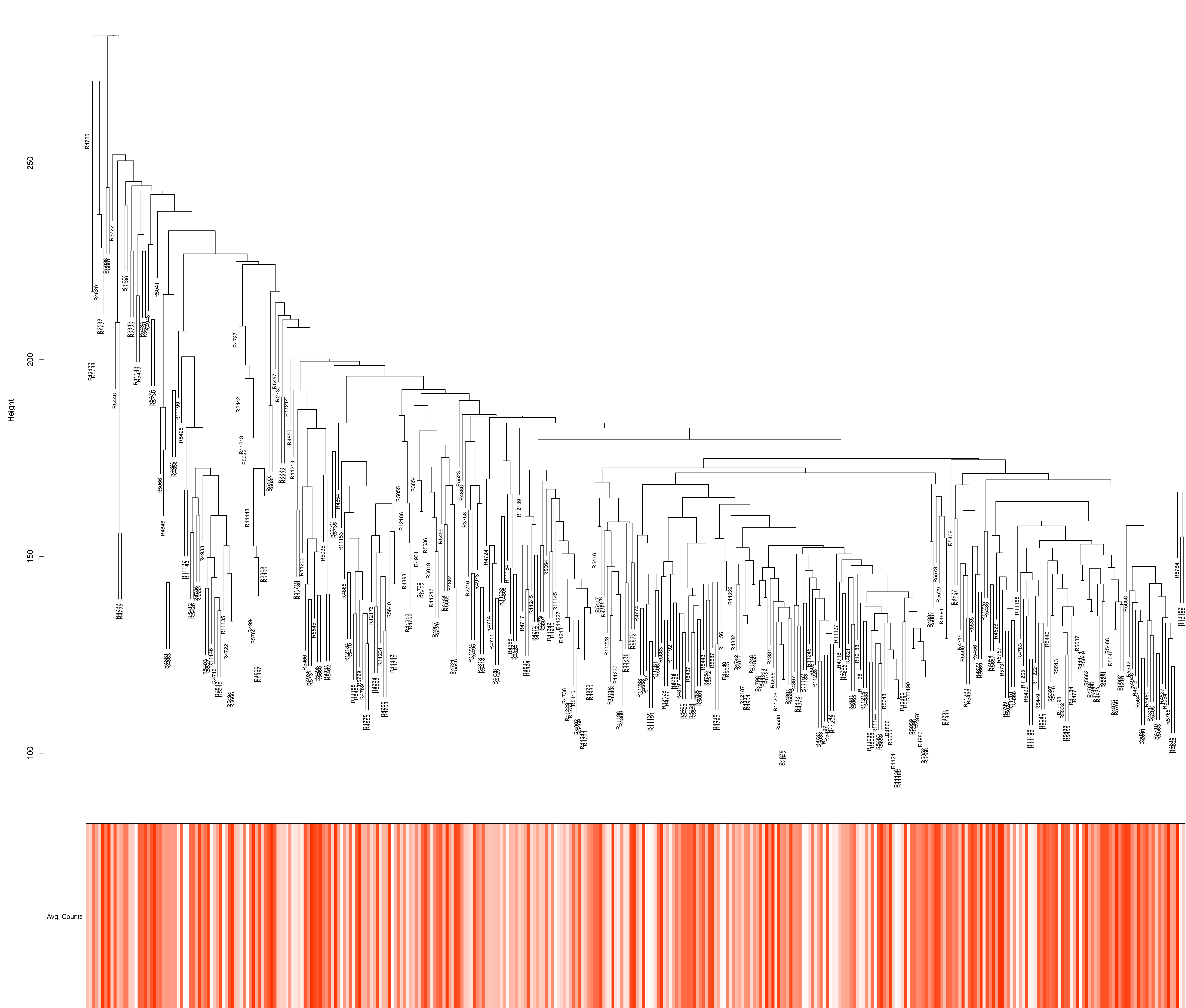

Supplement: Supplementary file 7 — Data S4 [file 41467_2024_48048_MOESM7_ESM.gz › wgcna_network_analysis/sex_network/hippocampus/sample_dendrogram_and_trait_heatmap.pdf]

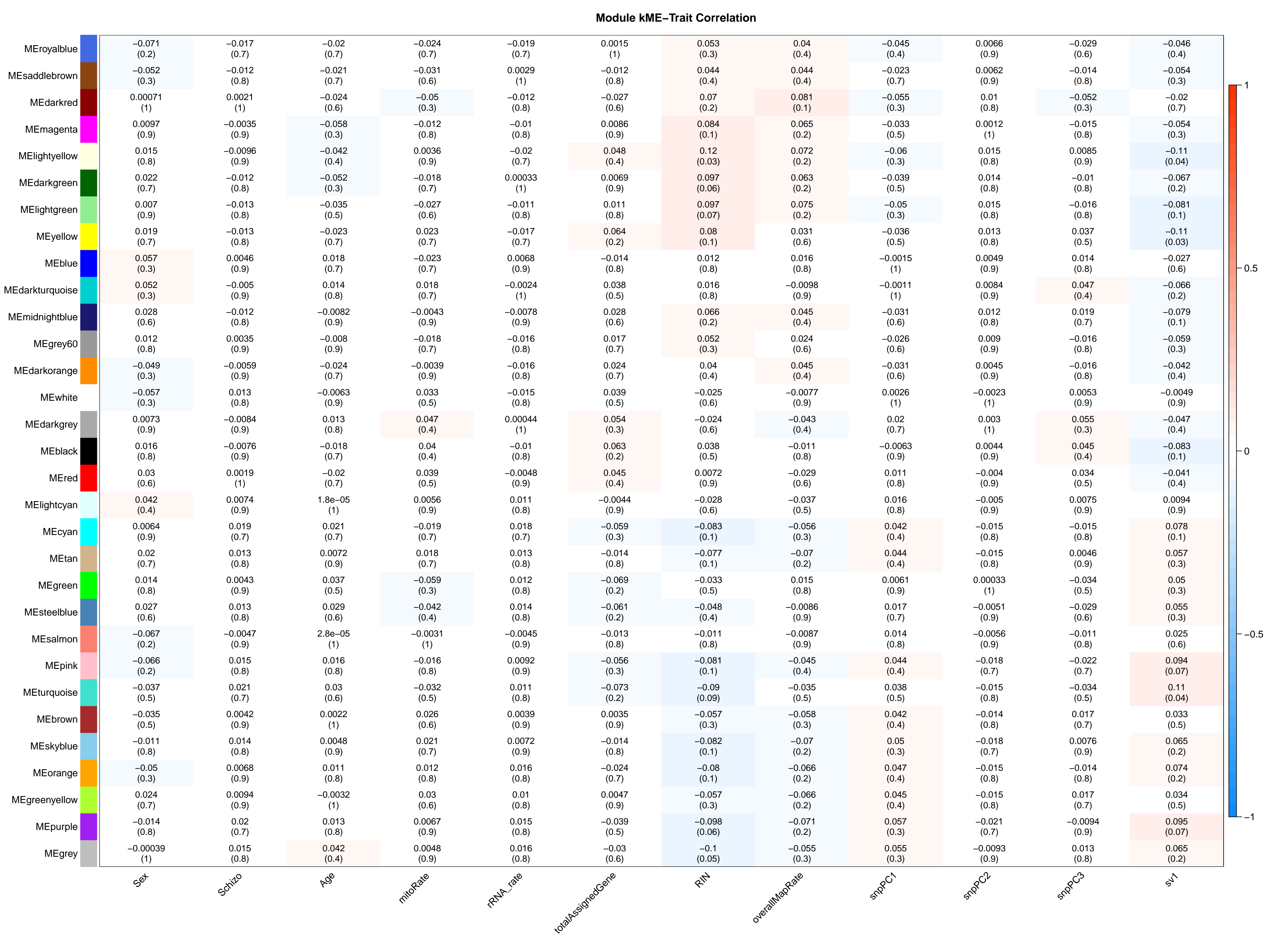

Supplement: Supplementary file 7 — Data S4 [file 41467_2024_48048_MOESM7_ESM.gz › wgcna_network_analysis/sex_network/hippocampus/module_trait_relationships.pdf]

# Enrichment/depletion DE genes in WGCNA modules (FDR values)

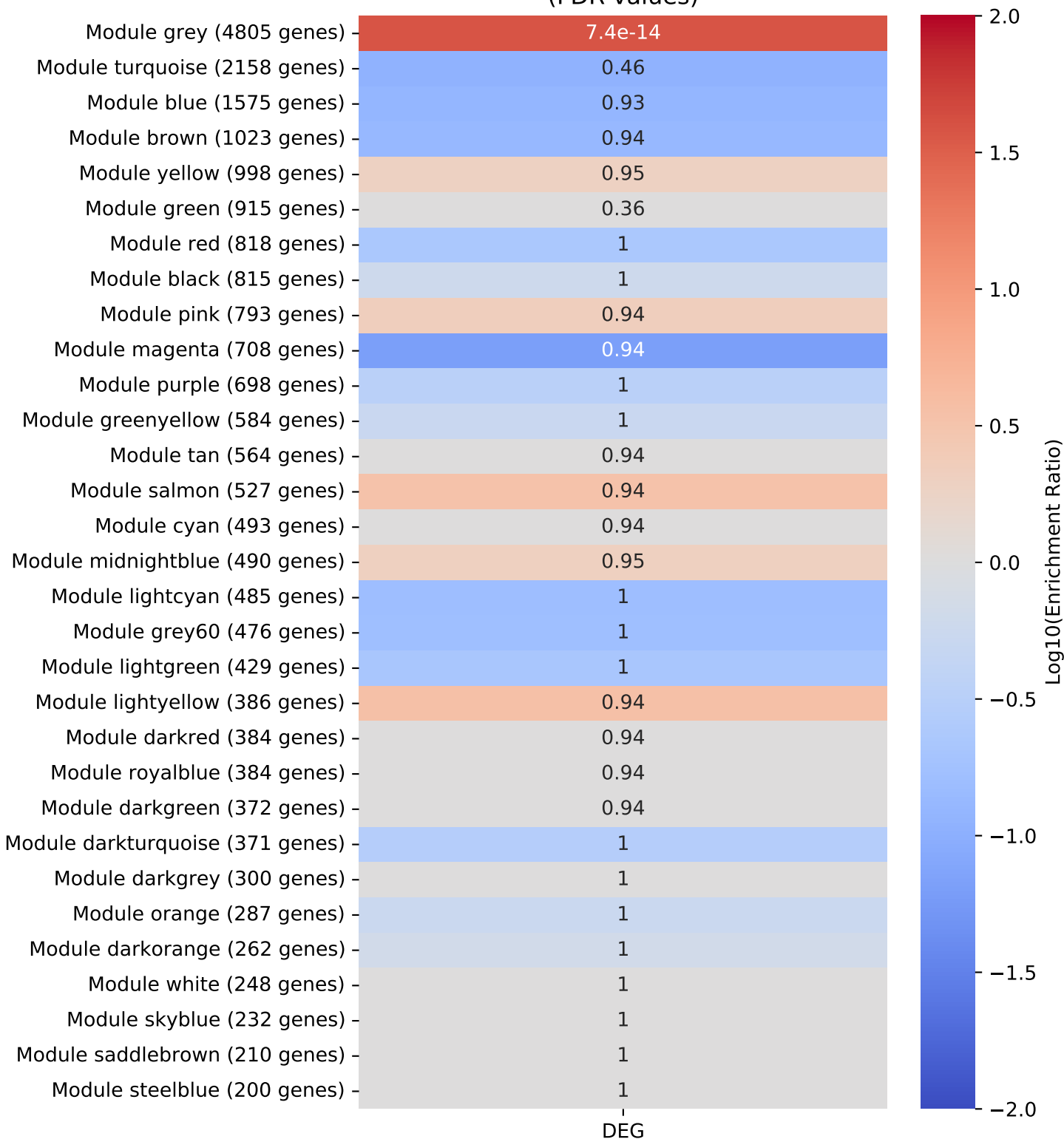

Supplement: Supplementary file 7 — Data S4 [file 41467_2024_48048_MOESM7_ESM.gz › wgcna_network_analysis/sex_network/hippocampus/wgcna_module_enrichment_DEG.pdf]

# Enrichment/depletion DE genes in WGCNA modules (FDR values)

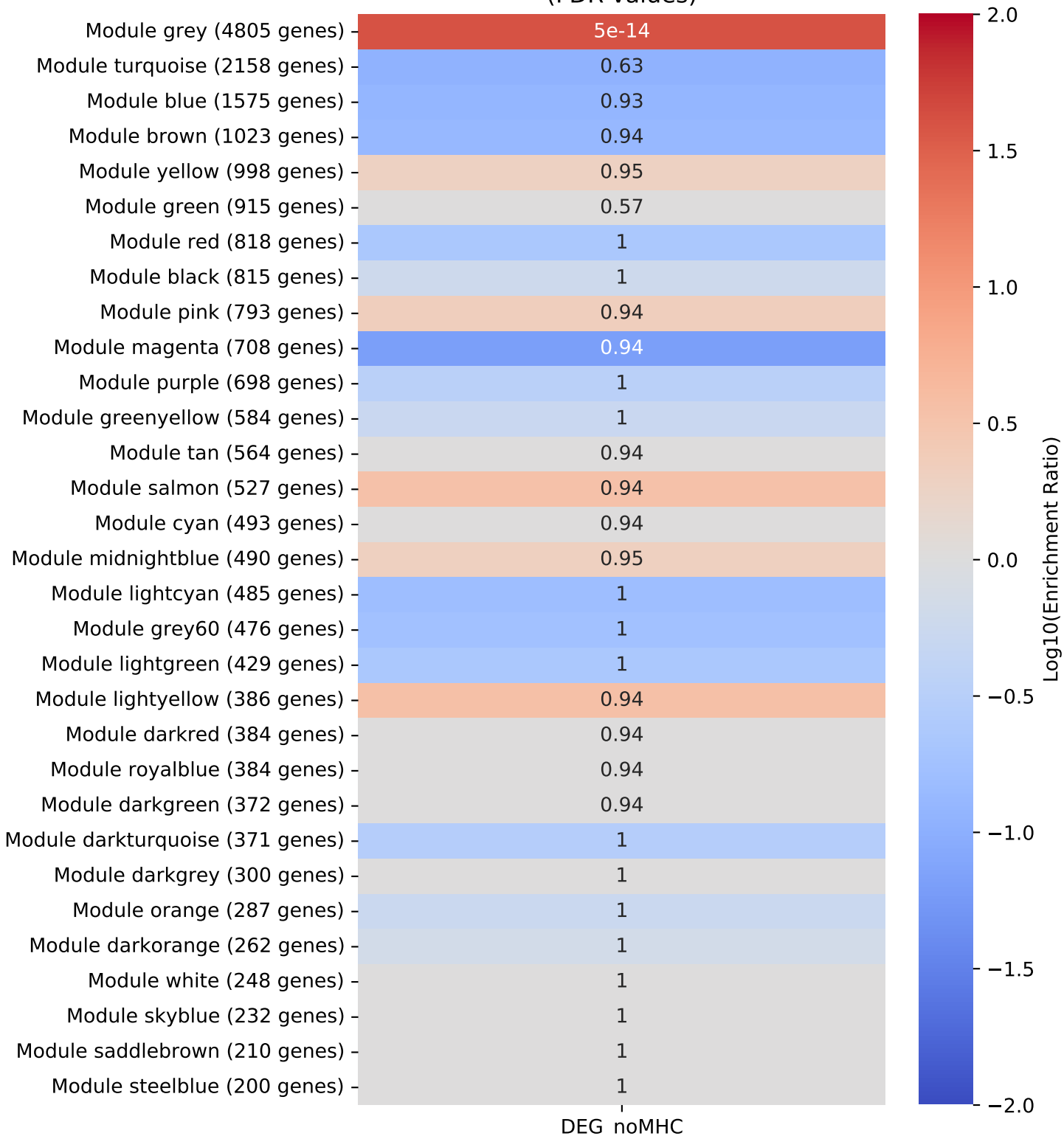

Supplement: Supplementary file 7 — Data S4 [file 41467_2024_48048_MOESM7_ESM.gz › wgcna_network_analysis/sex_network/hippocampus/wgcna_module_enrichment_DEG_noMHC.pdf]

Cluster Dendrogram

Height

1.00

0.95

0.90

0.85

0.80

0.75

Module Colors

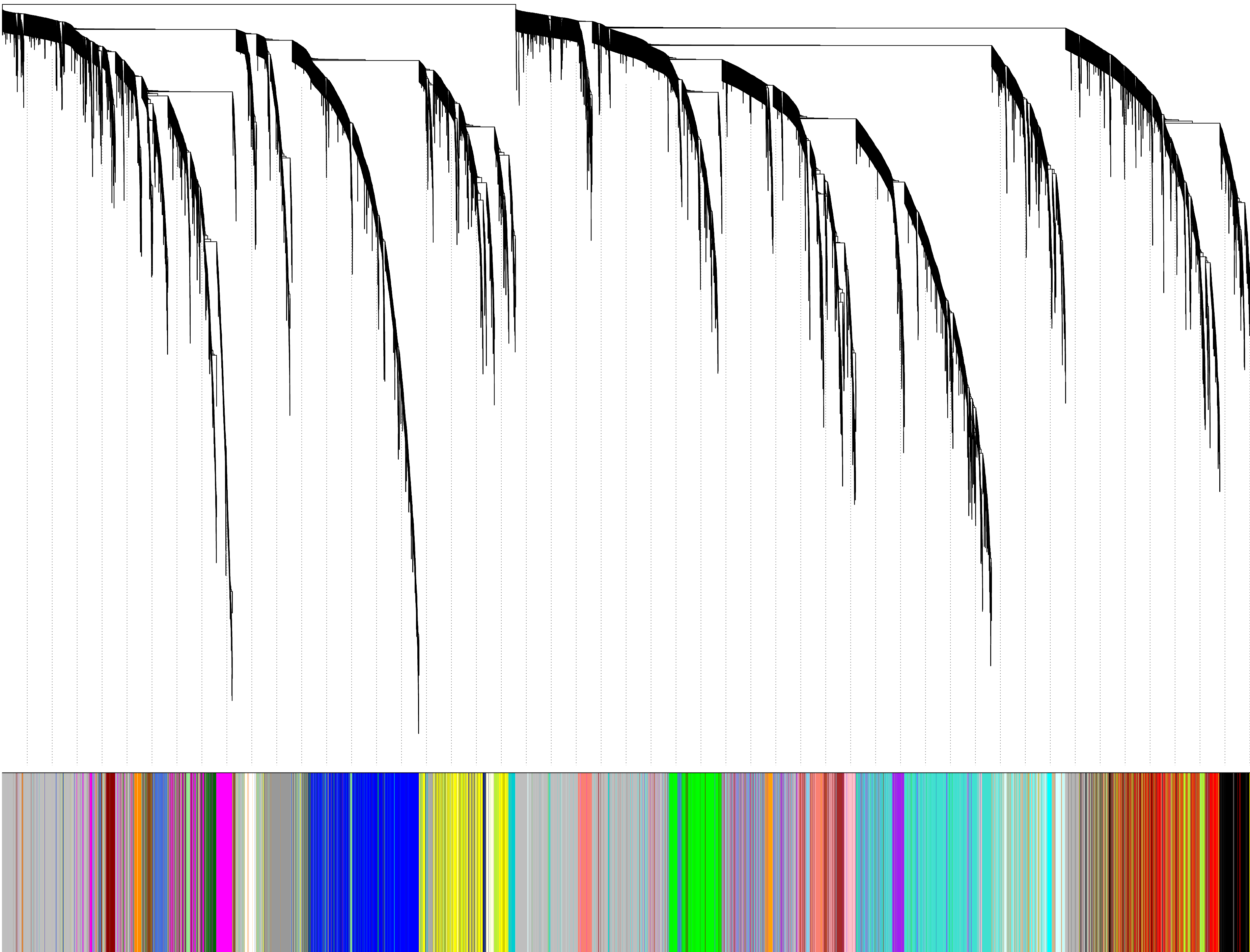

Supplement: Supplementary file 7 — Data S4 [file 41467_2024_48048_MOESM7_ESM.gz › wgcna_network_analysis/sex_network/hippocampus/cluster_dendrogram.pdf]

### Sample dendrogram and trait heatmap

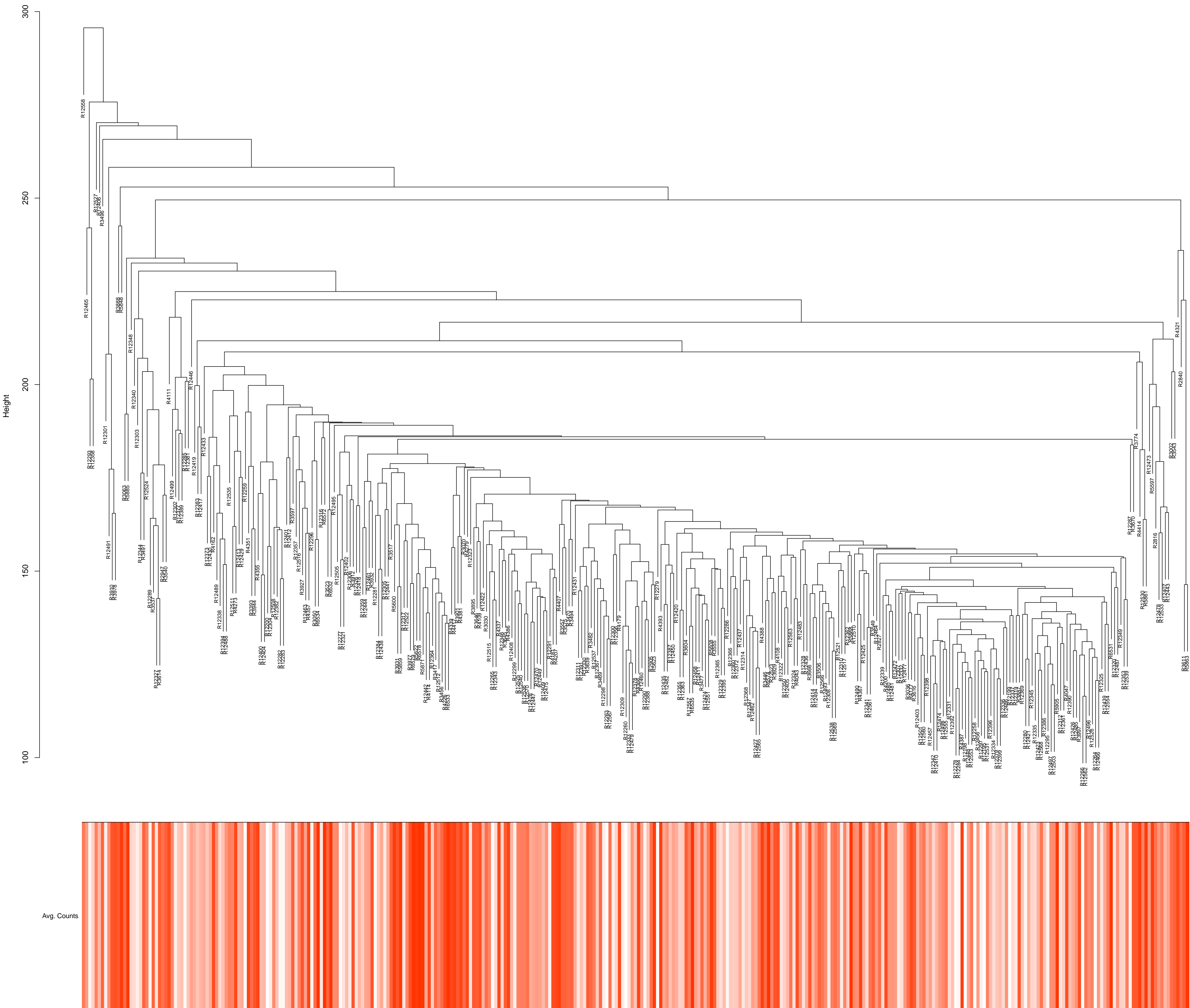

Supplement: Supplementary file 7 — Data S4 [file 41467_2024_48048_MOESM7_ESM.gz › wgcna_network_analysis/sex_network/dlpfc/sample_dendrogram_and_trait_heatmap.pdf]

Module kME–Trait Correlation

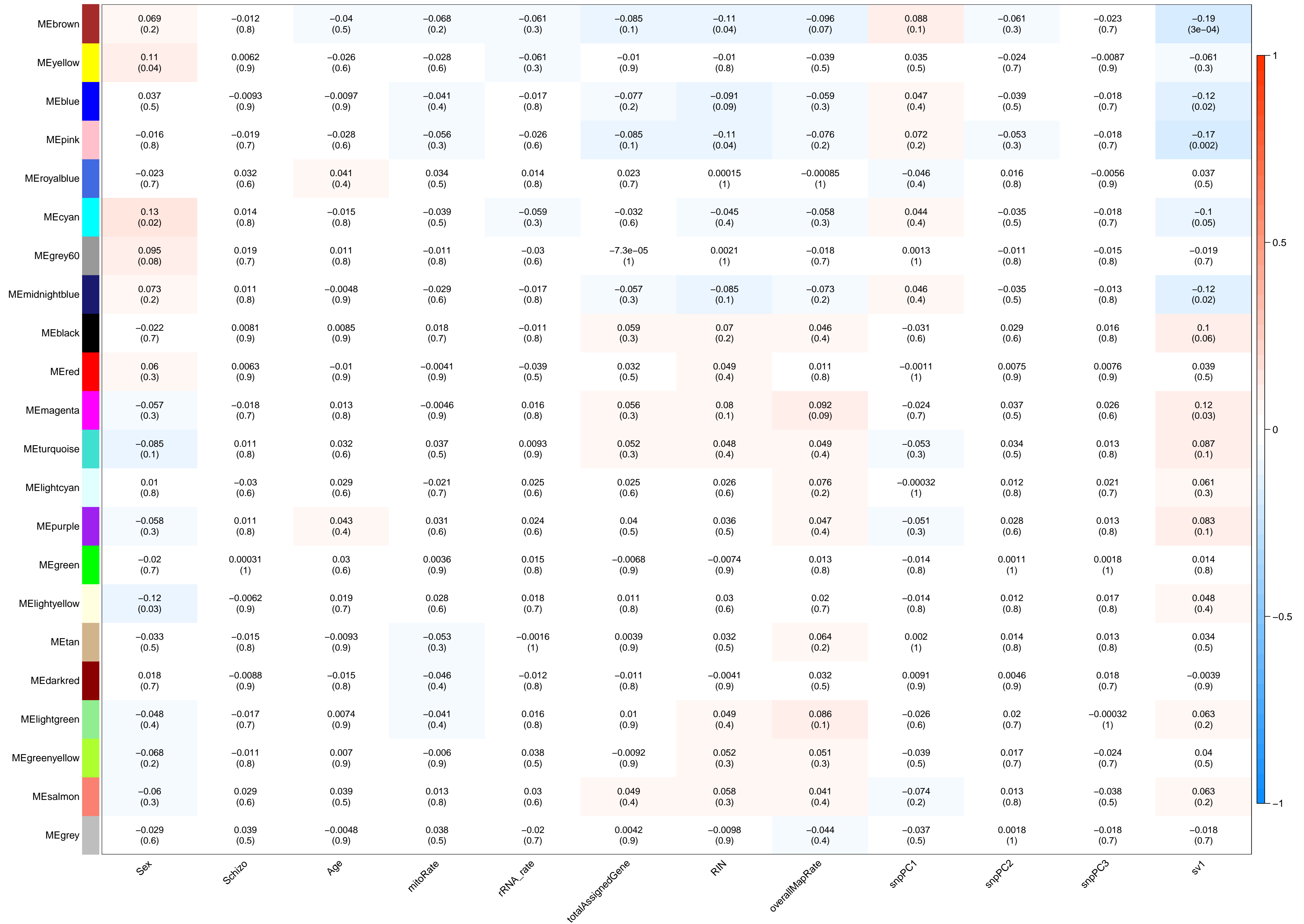

Supplement: Supplementary file 7 — Data S4 [file 41467_2024_48048_MOESM7_ESM.gz › wgcna_network_analysis/sex_network/dlpfc/module_trait_relationships.pdf]

Enrichment/depletion DE genes in WGCNA modules  
(FDR values)

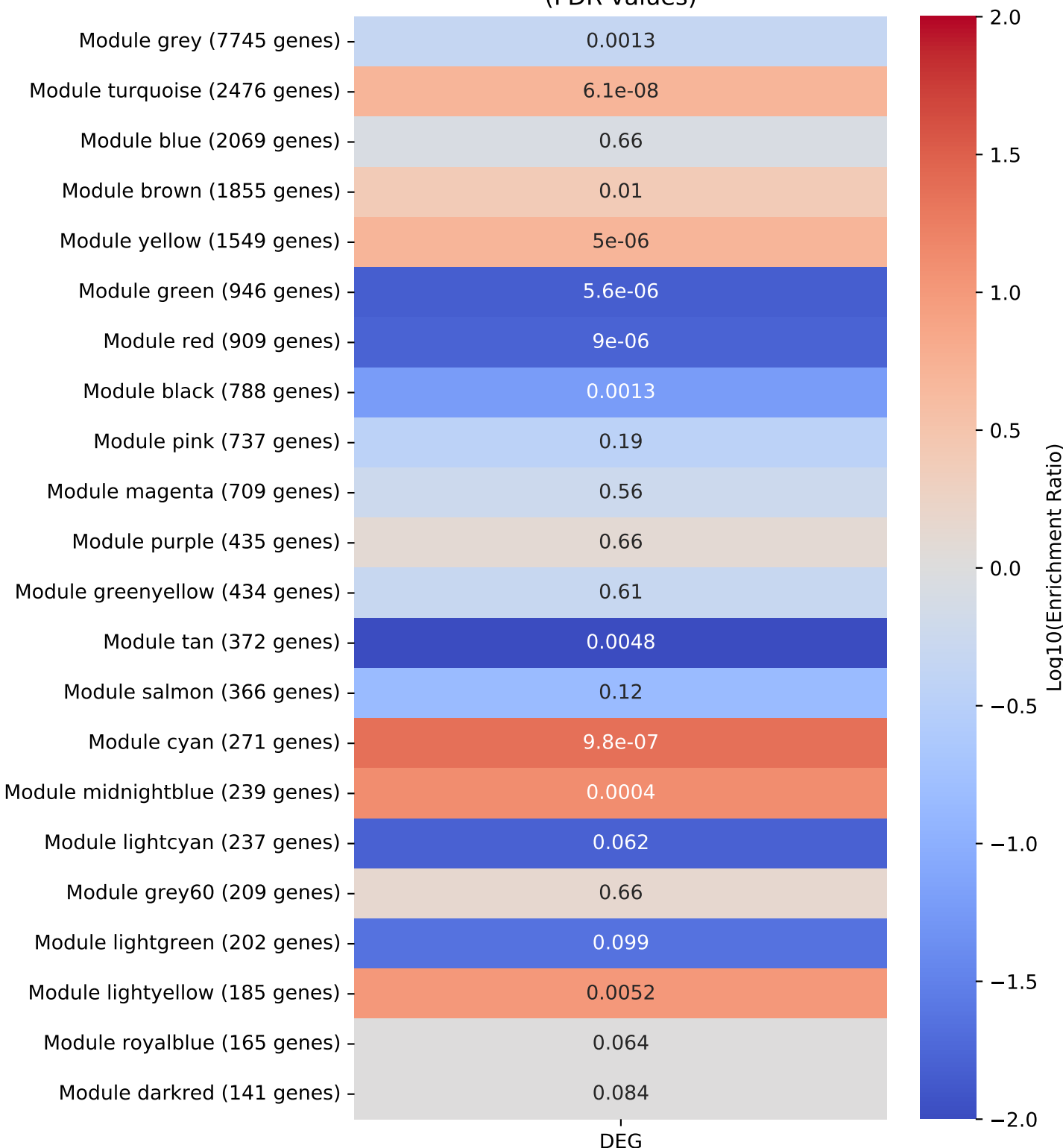

Supplement: Supplementary file 7 — Data S4 [file 41467_2024_48048_MOESM7_ESM.gz › wgcna_network_analysis/sex_network/dlpfc/wgcna_module_enrichment_DEG.pdf]

# Enrichment/depletion DE genes in WGCNA modules (FDR values)

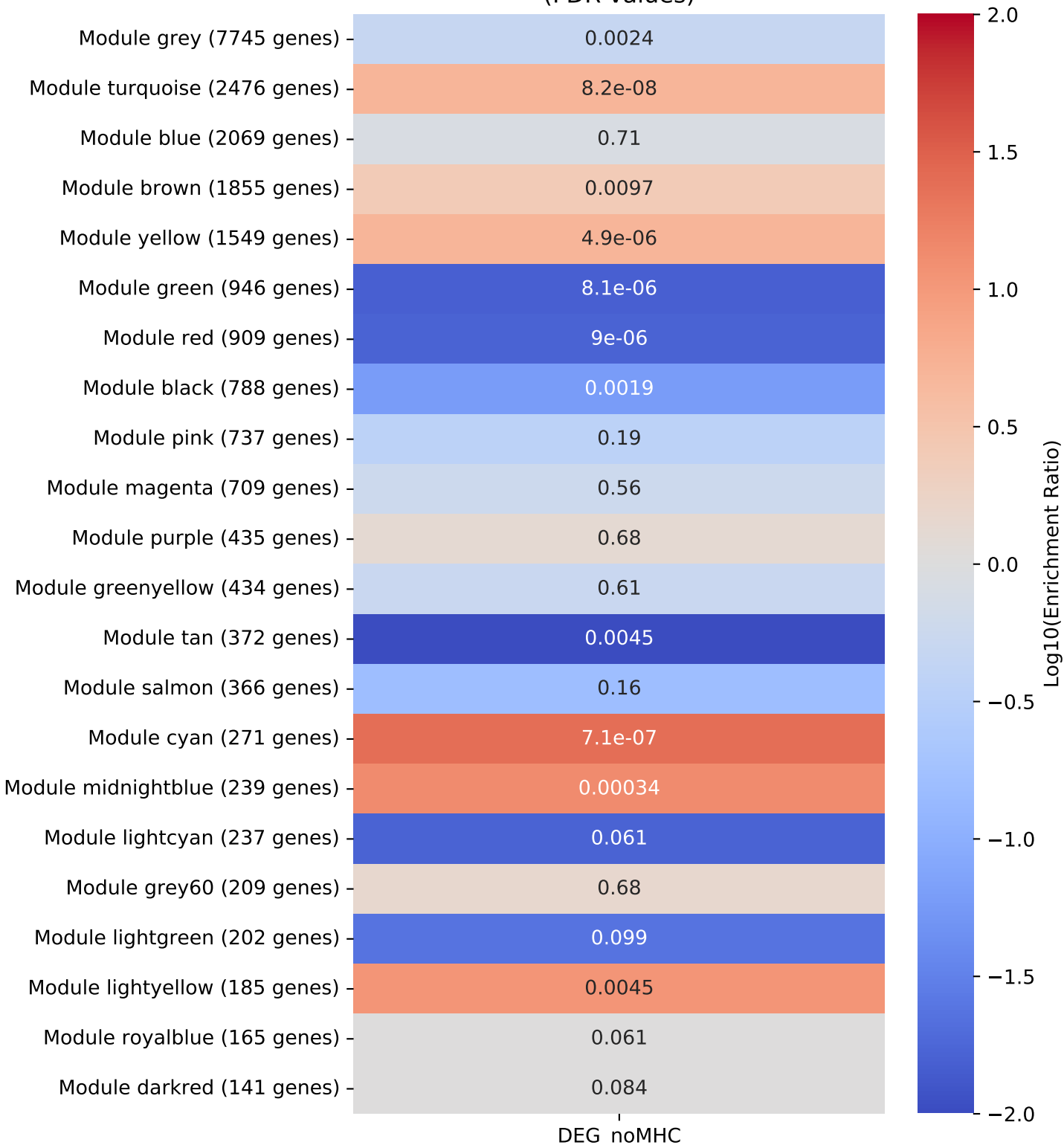

Supplement: Supplementary file 7 — Data S4 [file 41467_2024_48048_MOESM7_ESM.gz › wgcna_network_analysis/sex_network/dlpfc/wgcna_module_enrichment_DEG_noMHC.pdf]

Cluster Dendrogram

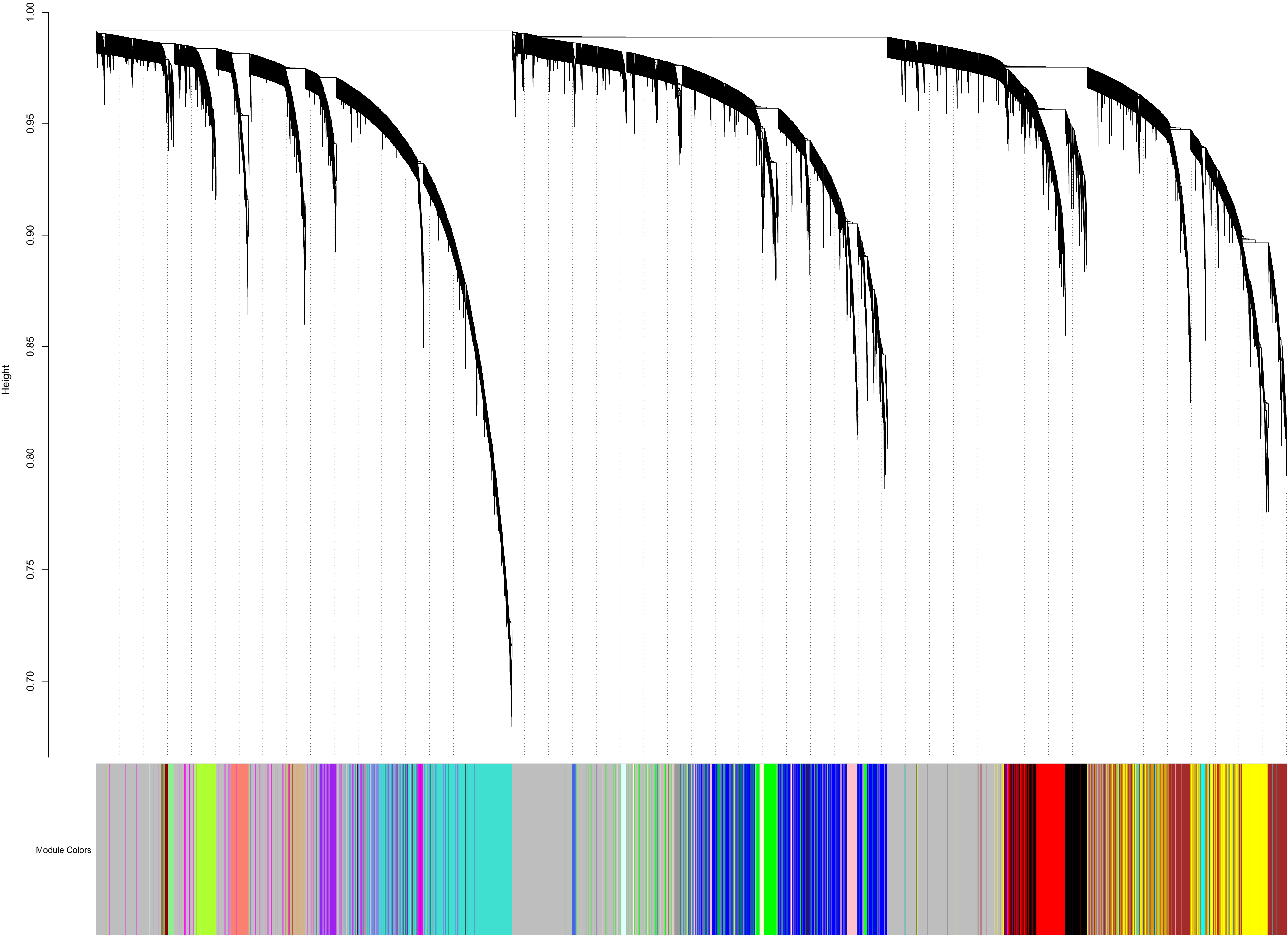

Supplement: Supplementary file 7 — Data S4 [file 41467_2024_48048_MOESM7_ESM.gz › wgcna_network_analysis/sex_network/dlpfc/cluster_dendrogram.pdf]

Sample dendrogram and trait heatmap

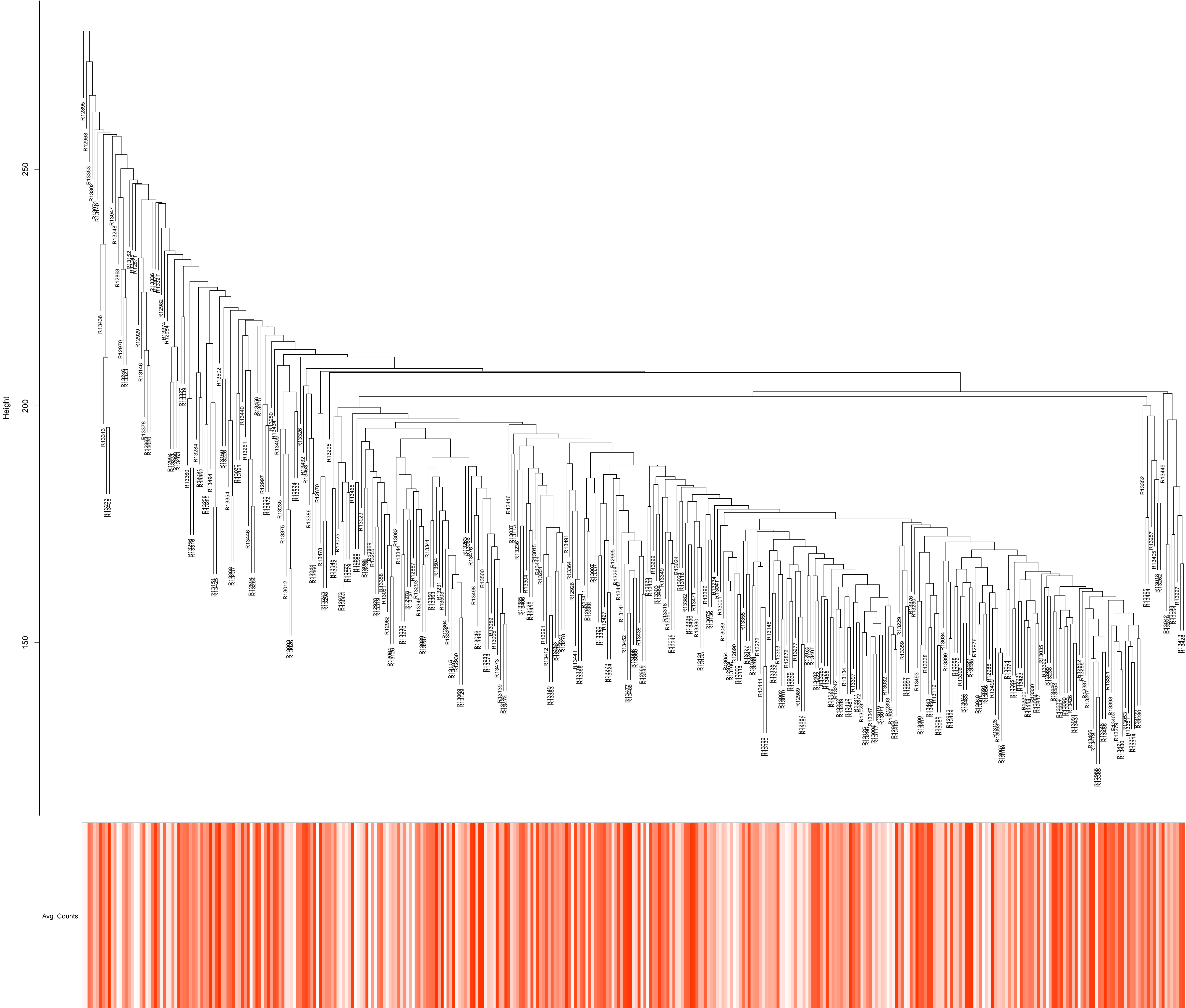

Supplement: Supplementary file 7 — Data S4 [file 41467_2024_48048_MOESM7_ESM.gz › wgcna_network_analysis/sex_network/caudate/sample_dendrogram_and_trait_heatmap.pdf]

Module kME–Trait Correlation

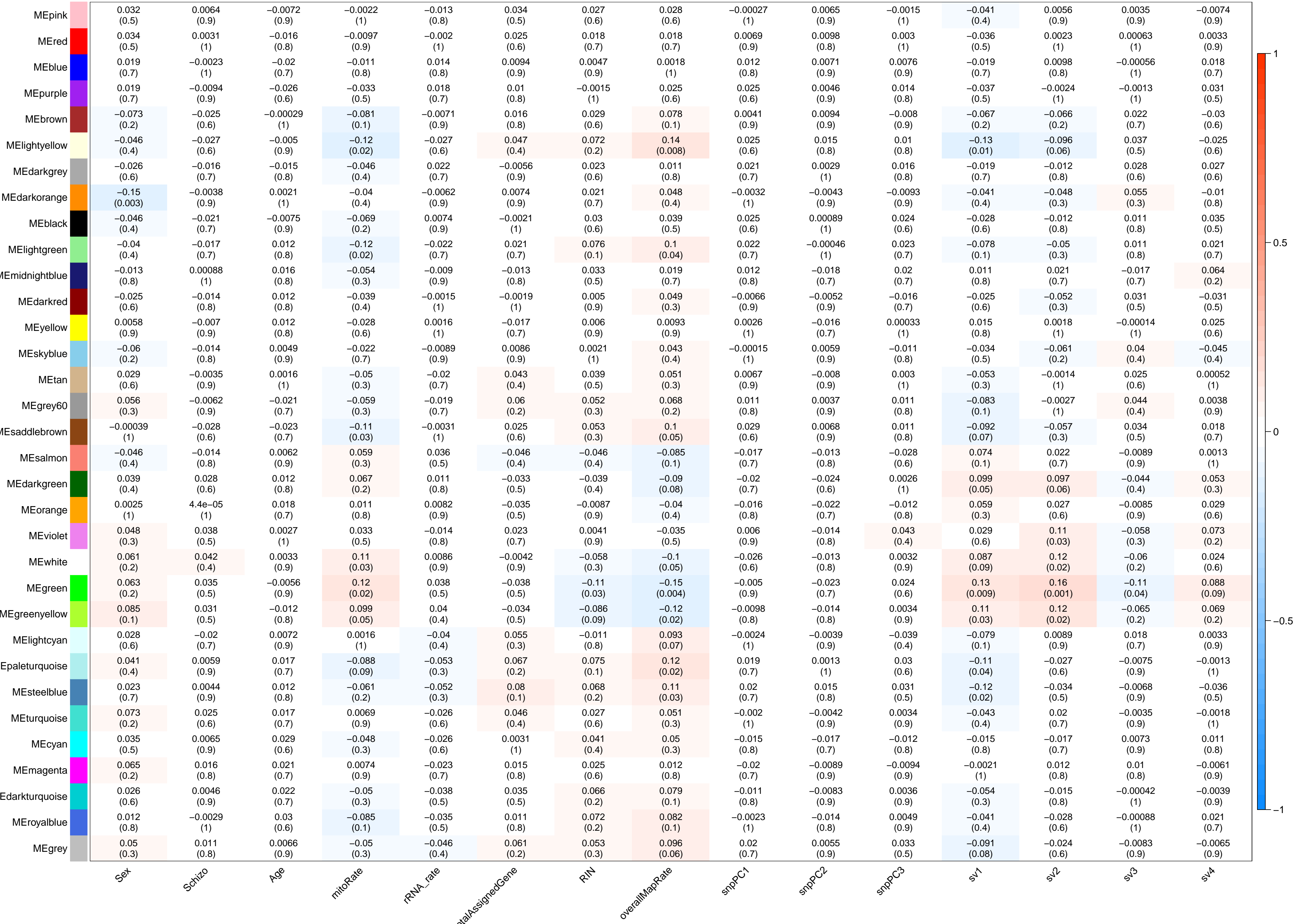

Supplement: Supplementary file 7 — Data S4 [file 41467_2024_48048_MOESM7_ESM.gz › wgcna_network_analysis/sex_network/caudate/module_trait_relationships.pdf]

Enrichment/depletion DE genes in WGCNA modules  
(FDR values)

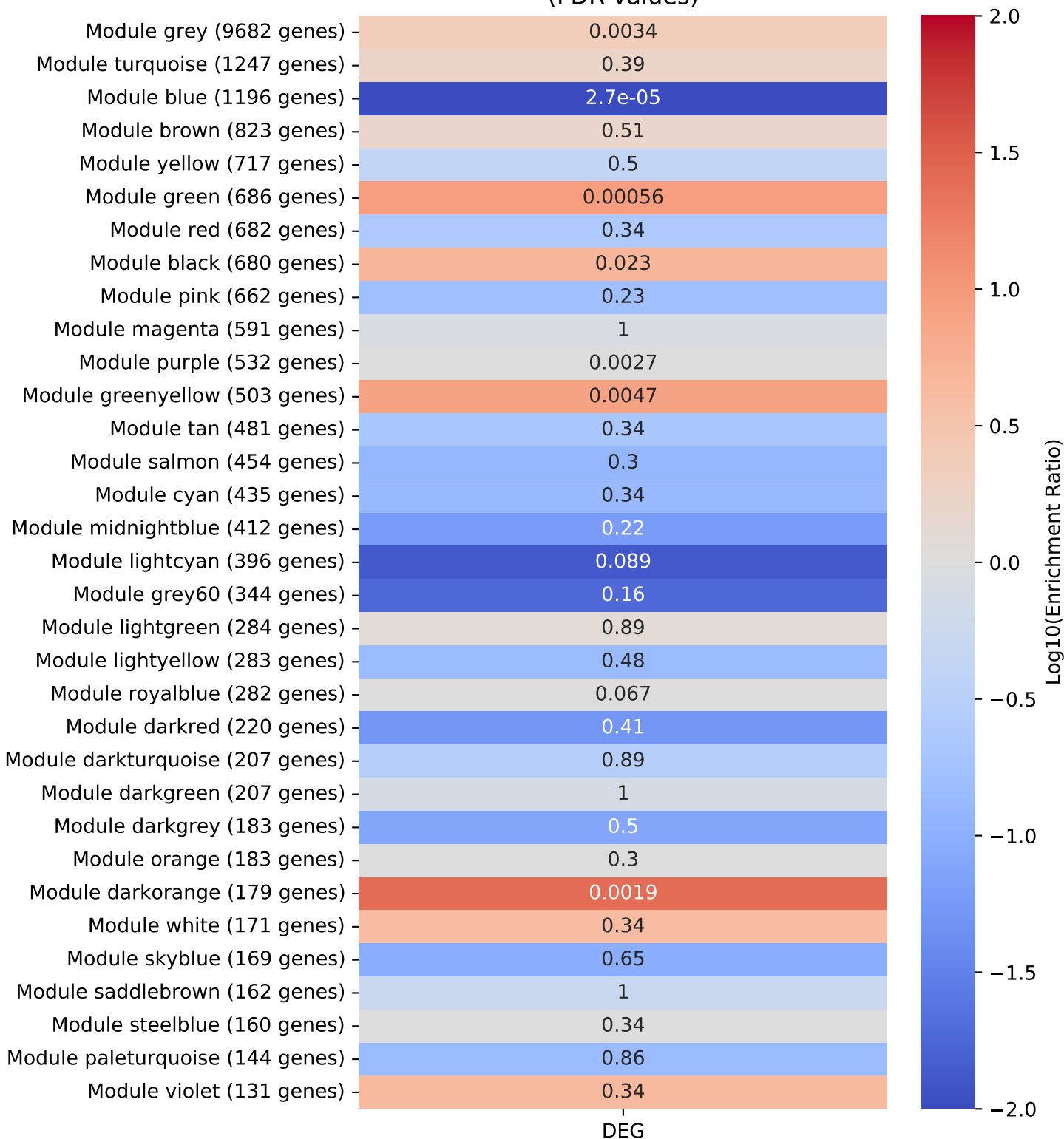

Supplement: Supplementary file 7 — Data S4 [file 41467_2024_48048_MOESM7_ESM.gz › wgcna_network_analysis/sex_network/caudate/wgcna_module_enrichment_DEG.pdf]

# Enrichment/depletion DE genes in WGCNA modules (FDR values)

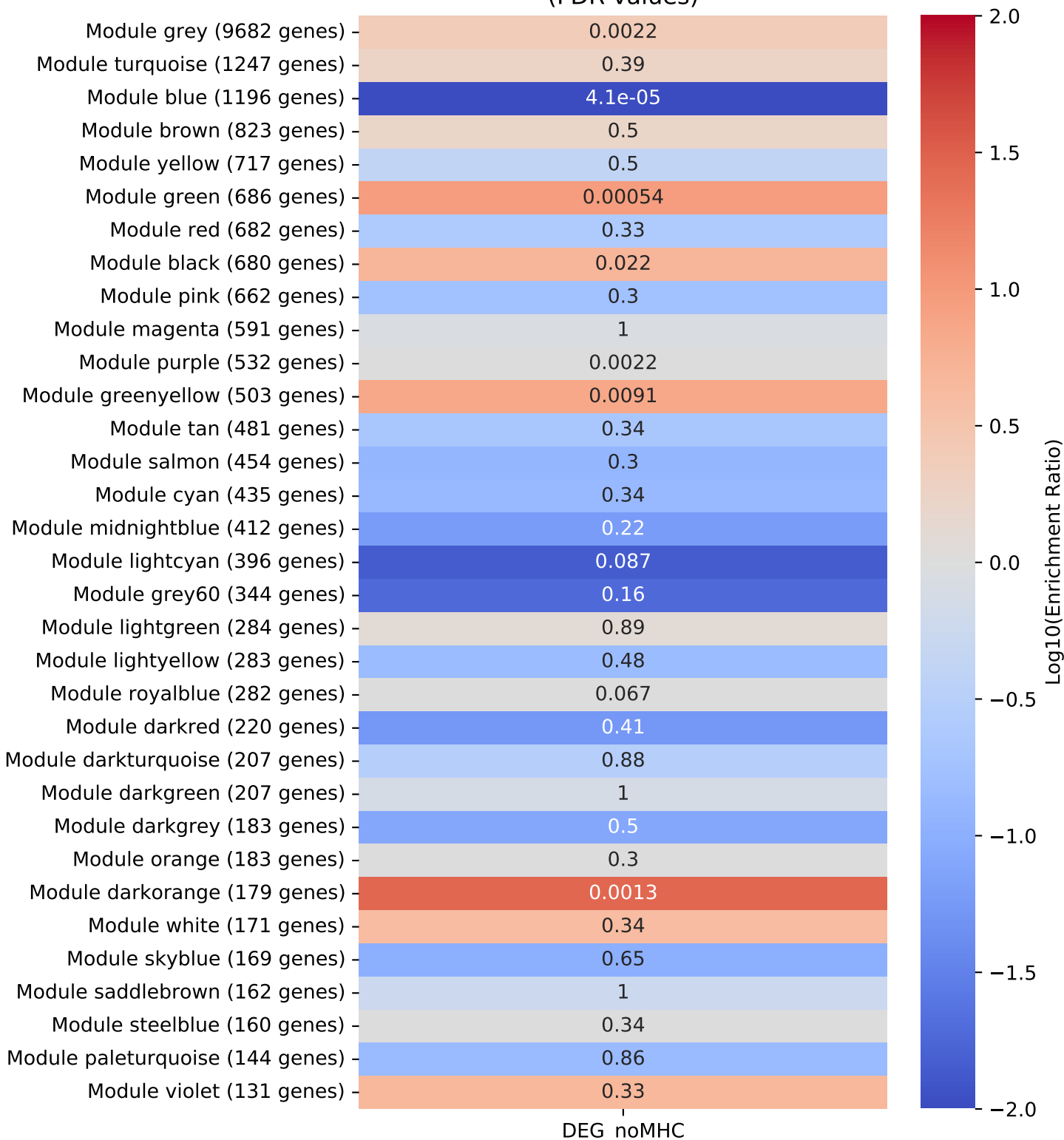

Supplement: Supplementary file 7 — Data S4 [file 41467_2024_48048_MOESM7_ESM.gz › wgcna_network_analysis/sex_network/caudate/wgcna_module_enrichment_DEG_noMHC.pdf]

**Scale independence**

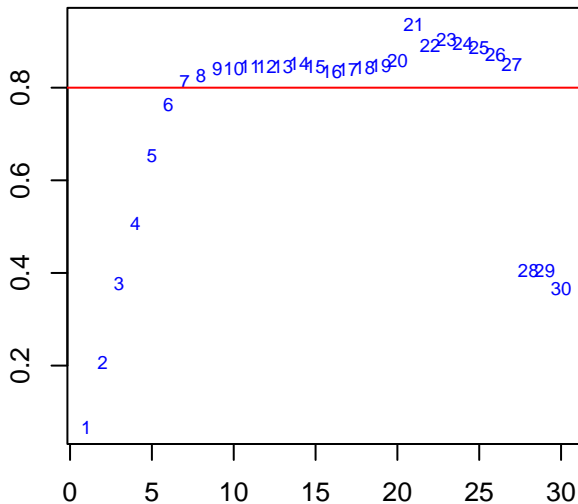

**Median connectivity**

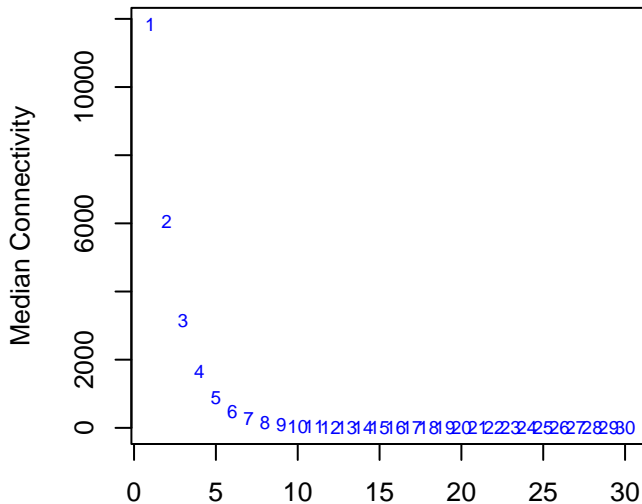

**Mean connectivity**

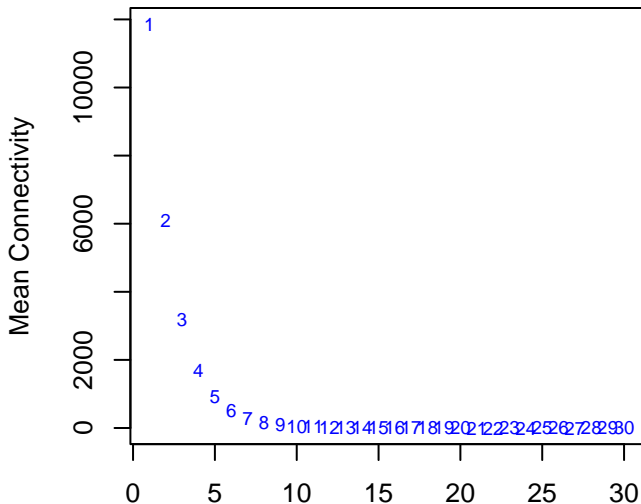

**Max connectivity**

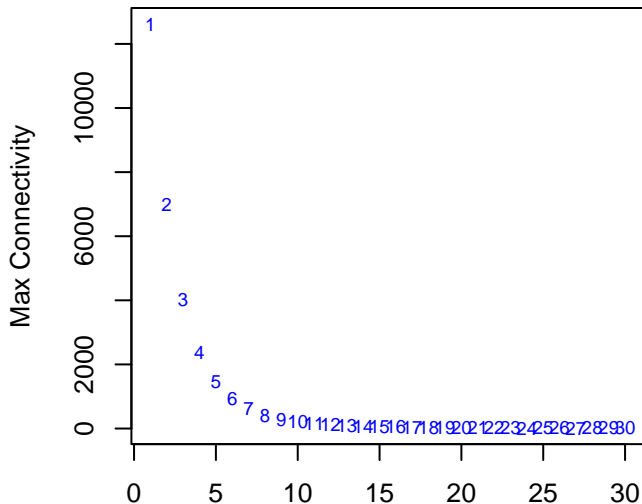

Supplement: Supplementary file 7 — Data S4 [file 41467_2024_48048_MOESM7_ESM.gz › wgcna_network_analysis/sex_network/caudate/power_parameter_selection.pdf]

Cluster Dendrogram

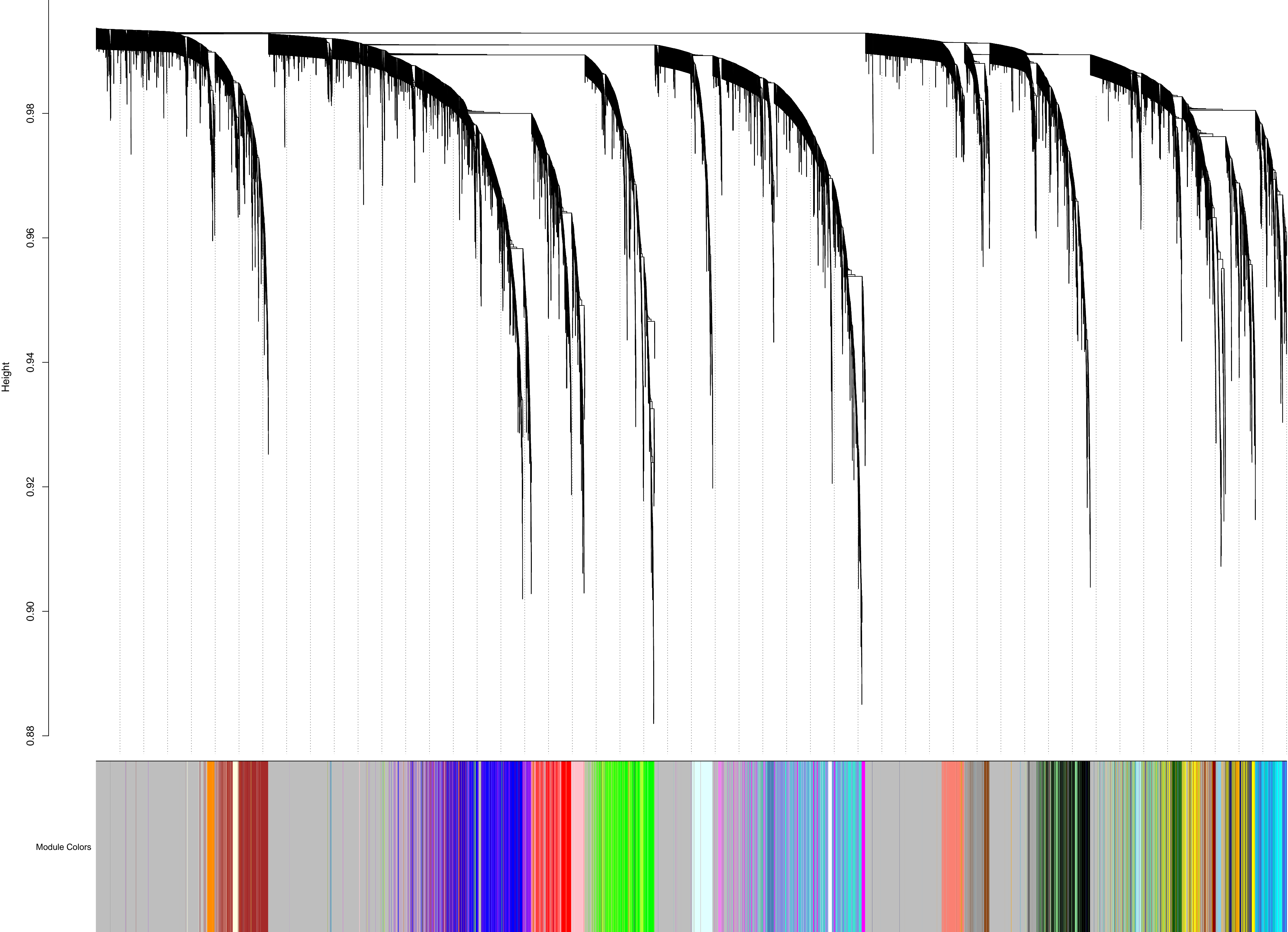

Supplement: Supplementary file 7 — Data S4 [file 41467_2024_48048_MOESM7_ESM.gz › wgcna_network_analysis/sex_network/caudate/cluster_dendrogram.pdf]
