# Supplementary material for: Sex affects transcriptional associations with schizophrenia across the dorsolateral prefrontal cortex, hippocampus, and caudate nucleus
Source: Nat Commun. 2024 May 10;15:3980. doi: 10.1038/s41467-024-48048-z (PMC11087501; doi:10.1038/s41467-024-48048-z)
Supplement: Supplementary file 19 — Reporting Summary [file 41467_2024_48048_MOESM19_ESM.pdf]

nature portfolio

Corresponding author(s): Kynon JM Benjamin, Apuã CM Paquola, Jennifer Erwin

Last updated by author(s): Feb 6, 2024

Reporting Summary

Nature Portfolio wishes to improve the reproducibility of the work that we publish. This form provides structure for consistency and transparency in reporting. For further information on Nature Portfolio policies, see our Editorial Policies and the Editorial Policy Checklist.

Statistics

For all statistical analyses, confirm that the following items are present in the figure legend, table legend, main text, or Methods section.

n/a	Confirmed	
☐	☒	The exact sample size (*n*) for each experimental group/condition, given as a discrete number and unit of measurement
☒	☐	A statement on whether measurements were taken from distinct samples or whether the same sample was measured repeatedly
☐	☒	The statistical test(s) used AND whether they are one- or two-sided<br>*Only common tests should be described solely by name; describe more complex techniques in the Methods section.*
☐	☒	A description of all covariates tested
☐	☒	A description of any assumptions or corrections, such as tests of normality and adjustment for multiple comparisons
☐	☒	A full description of the statistical parameters including central tendency (e.g. means) or other basic estimates (e.g. regression coefficient) AND variation (e.g. standard deviation) or associated estimates of uncertainty (e.g. confidence intervals)
☐	☒	For null hypothesis testing, the test statistic (e.g. *F*, *t*, *r*) with confidence intervals, effect sizes, degrees of freedom and *P* value noted<br>*Give P values as exact values whenever suitable.*
☒	☐	For Bayesian analysis, information on the choice of priors and Markov chain Monte Carlo settings
☒	☐	For hierarchical and complex designs, identification of the appropriate level for tests and full reporting of outcomes
☐	☒	Estimates of effect sizes (e.g. Cohen's *d*, Pearson's *r*), indicating how they were calculated

Our web collection on statistics for biologists contains articles on many of the points above.

Software and code

Policy information about availability of computer code

Data collection	No software was used for data collection.
Data analysis	Code used for the analyses presented in this paper is available at https://github.com/LieberInstitute/sex_differences_sz (DOI: 10.5281/zenodo.8410991).<br><br>Software packages used in data analysis:<br>HISAT2 (v2.2.1) for splice-aware alignment; RNA-SeQC (v2.4.2) for alignment quality control; kallisto (v0.46.2) for transcript quantification; featureCounts (v2.0.3) for counts quantification of genes and exons; RegTools (v0.5.3) for reference-free exon-exon junction quantification; TopMed Imputation server for genotype imputation (Eagle v2.4 and McCarthy Tools [https://www.well.ox.ac.uk/~wrayner/tools/HRC-1000G-check-bim-v4.3.0.zip]); PLINK2 (v2.00a3LM) for the majority of SNP processing; PLINK (v1.9) for MDS population stratification and matching gender phenotype to sex chromosomes<br>Jupyter Notebooks (v6.0.2) were used for visualization and are available in the GitHub repository; limma (R v4.2; v3.54.1) and edgeR (R v4.2; v3.40.2) for differential expression analysis (see jupyter notebook for fill environment list in GitHub); dream from variancePartition (R v4.2; v1.28.7) for brain region-specific differential analysis; dRFEtools (Python v3.7+; v0.1.17) and scikit-learn (Python 3.7+; v1.0.2) for autosomal sex prediction; WGCNA (R v4.2; v1.72) for network analysis; MAGMA (v1.10) for genomic annotation enrichment analysis; functional gene term enrichment with GSEA using clusterProfiler and DOSE (R v4.2; v4.6.2 and v3.24.2, respectively) for differential expression analysis and GOATOOLS (Python 3.7+; v1.2.3) for network analysis gene-term enrichment<br>tensorQTL for eQTL mapping (Python 3.7+; v1.0.7); mashr (R v4.2; v0.2.57) was used for sex-interacting eQTL; DAP-G (v1.0.0) for fine mapping; fastENLOC (v1.0) for colocalization analysis<br>matplotlib_venn (Python 3.8; v0.11.5) for venn diagrams; ComplexHeatmap (R v4.2; v2.6.2) for upset plots; ggpubr (R v4.2; v0.4.0) for box plots and scatterplots; circlize (R v4.2; v0.4.11) and ComplexHeatmap for circos plots; plotnine (Python 3.7+; v0.12.1) for enrichment

heatmaps comparing public datasets with BrainSeq Consortium analysis, RXE scatterplots, and sex-interacting eQTL box plots; RRHO2 (R v4.2; v1.0) and lattice (base R) for rank-rank hypergeometirc overlap analysis

For manuscripts utilizing custom algorithms or software that are central to the research but not yet described in published literature, software must be made available to editors and reviewers. We strongly encourage code deposition in a community repository (e.g. GitHub). See the Nature Portfolio guidelines for submitting code & software for further information.

Data

Policy information about availability of data

All manuscripts must include a data availability statement. This statement should provide the following information, where applicable:

- Accession codes, unique identifiers, or web links for publicly available datasets
- A description of any restrictions on data availability
- For clinical datasets or third party data, please ensure that the statement adheres to our policy

The processed counts (GENCODE v25, hg38), linked de-identified phenotype information, and RNA quality metrics used in this study are publicly available from the BrainSeq Consortium Phase 2 and 3 data releases as RangedSummarizedExperiment R objects. The Phase 2 data (total RNA DLPFC and hippocampus) are available for download at http://eqtl.brainseq.org/phase2/. The Phase 3 data (total RNA caudate nucleus) are available for download at http://erwinpaquolalab.libd.org/caudate_eqtl/. The reprocessed data (GENCODE v41, hg38) is also available as an RangedSummarizedExperiment R object and is available as part of Source Data. Analysis-ready genotype data are available under restricted access to protect research subjects, access can be obtained through dbGaP accession phs000979.v3.p2. FASTQ files are also available under restricted access to protect research subject. For Phase 2 total RNA DLPFC and hippocampus, researchers can access to FASTQ files via the Globus collections jhpce#bsp2-dlpfc and jhpce#bsp2-hippo at https://research.libd.org/globus/. For Phase 3 caudate nucleus, researchers can obtain access to FASTQ files via dbGaP accession phs003495.v1.p1. PGC3 GWAS summary statistics are available at https://figshare.com/articles/dataset/scz2022/19426775. The nominal eQTL, predictive analysis, network analysis, and differential expression analysis generated in this study are provided in the Supplementary Information/Source Data file. They are also available at https://doi.org/10.5281/zenodo.10574013 or http://erwinpaquolalab.libd.org/3region_sex/. Source Data are provided at https://doi.org/10.5281/zenodo.10574013.

Research involving human participants, their data, or biological material

Policy information about studies with human participants or human data. See also policy information about sex, gender (identity/presentation), and sexual orientation and race, ethnicity and racism.

Reporting on sex and gender	To match gender identity with sex chromosomes, we applied the sex imputation function (--check-sex) from PLINK (v1.9). This compares sex assignments in the input dataset with those imputed from X-chromosome inbreeding coefficients. We used a Jupyter Notebook with the R kernel to compare self-reported gender identity with genotype imputed sex (F estimates). Here, we found all gender identities matched sex chromosomes with F estimates for females below 0.22 and males above 0.9. As such, we used the self-reported gender identity (as reported by medical examiner's office) as sex marker for the analysis. No individuals were removed by this analysis process.
Reporting on race, ethnicity, or other socially relevant groupings	As a diverse dataset, our donor population includes individuals of African and European ancestry collected from the United States of America. We do not use race as reported by the medical examiner's office in our models. We accounted for variation in the human population underlying genetic variation with genetic similarity measurements. Genetic similarity was measured using global population structure via multidimensional scaling (MDS) with PLINK (v1.9) on linkage disequilibrium (LD)- independent variants. The first component separated samples by race as reported by the medical examiner's office. The first 3 components from MDS analysis were used within our linear models (eQTL and expression analyses) as a population characteristic.
Population characteristics	A total of 1,170 samples were used in the eQTL portion of this study, 399 were from the caudate nucleus, 377 from the DLPFC, and 394 from the hippocampus. Out of the 1,170 samples, 126 [76 CTL/50 SZ], 121 [73 CTL; 48 SZ], and 126 [79 CTL; 47 SZ] were female and 273 [169 CTL; 104 SZ], 256 [156 CTL; 100 SZ], 268 [182 CTL; 86 SZ] were male from the caudate nucleus, DLPFC, and hippocampus, respectively. The average age for female individuals was 50.2 (±16.9 sd), 48.4 (±15.7 sd), and 47.9 (±16.7 sd) for the caudate nucleus, DLPFC, and hippocampus, respectively. The average age for male individuals was 48.6 (±15.7 sd), 44.6 (±16.1 sd), and 44.4 (±16.2 sd) for the caudate nucleus, DLPFC, and hippocampus, respectively. A detailed sample breakdown is provided in Table 1.<br><br>For the 1,127 samples used in the expression analyses of this study, 393 [239 CTL; 154 SZ], 359 [211 CTL; 148 SZ], and 375 [242 CTL; 133 SZ] samples were located in the caudate nucleus, DLPFC, and hippocampus, respectively. The mean age for individuals was 49.6 (± 15.6 sd), 47.4 (± 15.4 sd), and 47.0 (± 15.3 sd) for the caudate nucleus, DLPFC, and hippocampus, respectively. A detailed sample breakdown is provided in Table 3.
Recruitment	All specimens used in this study were obtained with informed consent from the next kin. See below for more details.
Ethics oversight	The research described herein complies with all relevant ethical regulations. All specimens used in this study were obtained with informed consent from the next of kin under protocols No. 12-24 from the Department of Health and Mental Hygiene for the Office of the Chief Medical Examiner for the State of Maryland and No. 20111080 for the Western Institutional Review Board for the Offices of: 1) the Chief Medical Examiner for Kalamazoo Michigan, 2) University of North Dakota in Grand Forks North Dakota, and 3) Santa Clara County California.

Note that full information on the approval of the study protocol must also be provided in the manuscript.

Field-specific reporting

Please select the one below that is the best fit for your research. If you are not sure, read the appropriate sections before making your selection.

☒ Life sciences ☐ Behavioural & social sciences ☐ Ecological, evolutionary & environmental sciences

For a reference copy of the document with all sections, see nature.com/documents/nr-reporting-summary-flat.pdf

Life sciences study design

All studies must disclose on these points even when the disclosure is negative.

Sample size

We did not predetermine sample size using statistical methods. For the gene expression analysis, we included all adult samples (age > 17 years) that passed quality control, totaling 1,127 (480 unique individuals). For the eQTL analysis, we expanded the sample size by including individuals aged 13 to 17 at the time of death, resulting in 1,170 (504 unique individuals). This decision was based on the understanding that eQTLs are less susceptible to subtle population characteristic compared to gene expression analysis. This difference in susceptibility arises from eQTLs reflecting genetic variations while expression is highly influenced by environmental factors.

Of all 1,170 samples (504 unique individuals) used in the eQTL portion of this study, 399 were from the caudate nucleus, 377 from the DLPFC, and 394 from the hippocampus. Out of the 1,170 samples, 126, 121, and 126 were female and 273, 256, 268 were male from the caudate nucleus, DLPFC, and hippocampus, respectively. For the 1,127 samples (480 unique individuals) used in the expression analyses (DE, XCI, dosage compensation, network analysis, and machine learning [dRFEtools]) of this study, 393, 359, and 375 samples were located in the caudate nucleus, DLPFC, and hippocampus, respectively. More details for sample breakdown are found in Tables 1 and 3.

Data exclusions

We selected samples from the caudate nucleus, DLPFC, and hippocampus based on four inclusion criteria: 1) used RiboZero RNA-sequencing library preparation, 2) features an age greater than 13 years, 3) has a self-reported ethnicity of either African American or White American, and 4) has TOPMed imputed genotypes available. This resulted in a total of 1,170 samples from 504 unique individuals across the three brain regions for eQTL analysis. For expression-based analysis, we excluded individuals with age less than 17 years to include only adult individuals, resulting in a total of 1,127 samples from 480 unique individuals across the three brain regions. This minimizes the effect of neurodevelopmental changes on expression analysis while still allowing for greater power in the eQTL analysis, which is not as affected by the inclusion of these individuals.

Replication

Sex differences in the brain replication:
We downloaded differential expression results for sex differences from the supplemental materials for Trabzuni et al., Mayne et al., and Gershoni and Pietrokovski. For replication analysis, we compared the DEGs with these previous sex differences analysis in the brain (refs 30–32) and found greater than 62% of DEGs were significantly differentially expressed in all brain regions except for the GTEx cerebellum and anterior cingulate cortex (Fig. S9) with a concordant direction of effect between BrainSeq Consortium and GTEx brain regions (Fisher's exact test, p-value < 0.01).

For a more in-depth comparison, we examined the sex differences found using the CMC DLPFC (ref 6; Hoffman et al.). For CommonMind Consortium replication of differential expression analysis, we downloaded differential expression results for sex differences from Hoffman et al., as well as normalized expression from Synapse (syn18103849). We also discovered a large number of DEGs on sex chromosomes (39 of 51 [76.5%] and 41 of 54 [75.9%] for the NIMH HBCC and MSSM-Penn-Pitt cohorts, respectively) similar to our BrainSeq Consortium analysis. Additionally, we observed significant pairwise enrichment of these CMC DEGs with our BrainSeq Consortium DEGs across brain regions (Fisher's exact test, p-value < 0.01; Fig. S10). We found high replication with X- and Y-linked genes; however, autosomal DEGs were less likely to replicate in different datasets.

At significant levels (adjusted p-value < 0.05), all directions agreed between the CMC DLPFC and the BrainSeq Consortium brain regions with a significant positive correlation (Spearman; rho > 0.97 for all pairwise comparisons; p-value < 1.1e-44; Fig. S13). In summary, the direction of change for sexually dimorphic genes is generally shared across multiple brain regions and independent datasets.

XCI and dosage compensation replication:
We downloaded gene TPM from the GTEx v8 portal (https://www.gtexportal.org/home/datasets), as well as sample phenotype information. We replicated differences in chromosome-wide dosage by comparing the relative X chromosome expression (RXE) to autosomes. we observed a significant decrease of RXE in male individuals only in the DLPFC (Mann-Whitney U, p-value = 0.047), demonstrating region-specific dosage compensation. We also observed a similar trend of decreased RXE in the DLPFC from the CMC MSSM-Penn-Pitt cohort (Mann-Whitney U, p-value = 0.07; Fig. S18A) but not the GTEx frontal cortex (Fig. S19). Even so, the large RXE variation across the 13 GTEx brain regions demonstrated region-specific dosage compensation (Fig. S19).

Interaction of schizophrenia and sex in the brain replication:
After investigating sex differences in the brain without consideration of diagnosis in 480 unique individuals (caudate nucleus [n=393], DLPFC [n=359], and hippocampus [n=375]), we next identified statistically significant differentially expressed features (adjusted p-value < 0.05) with respect to sex differences and diagnosis through an interaction model. No genes, transcripts, or exons were significant by this interaction model, similar to a previous study (ref 6; Hoffman et al.). While overall replication with CMC DLPFC was limited, we found significant correlation of nominally significant (p-value < 0.05) transcriptional signatures between DLPFC and CMC DLPFC, NIMH HBCC cohort (π_1 = 0.51; Spearman, ρ = 0.60, p-value < 0.01; Fig. S20 and Table S5).

Sex-specific schizophrenia replication:
For sex-specific schizophrenia replication, we downloaded Qin et al. results. We compared our results with the recent meta-analysis for sex-specific schizophrenia DEGs in the prefrontal cortex (ref 7; Qin et al.). Of the 46 male-specific DEGs identified by Qin et al., we found a total of three overlapping genes: one gene overlapping (PARD3) with the caudate nucleus stringent female-specific DEGs and two overlapping genes (USE1 and ABCG2) with the hippocampus stringent male-specific DEGs, which all shared direction of effect. When we compared the full set of female and male schizophrenia DEGs across brain regions, we found an additional three overlapping genes (CD99, GABARAPL1, and LIN7B)

shared with the caudate nucleus. Of these three only GABARAPL1 had a discordant direction of effect.

Sex-dependent eQTL replication:
For sex-interacting eQTL, we downloaded results from Trabzuni et al., Yao et al., Kukurba et al., and Shen et al.. When we compared our si-eQTL with previous work in whole blood and in lymphoblastoid cell lines, we found no overlap with the 19 si-eQTL identified in whole blood (ref 17,18) and two genes (ATG4C and CA2) of the 21 si-eQTL identified in lymphoblastoid cell lines (ref 19) and also present in the caudate nucleus si-eQTL. We next compared our results with the four si-eQTL (q-value < 0.25) identified in GTEx brain regions (amygdala and nucleus accumbens basal ganglia) (ref 40) and found no overlaps. When we expanded to the 369 si-eQTL (q-value < 0.25) from all 43 GTEx tissues (ref 40), we found two overlapping genes encoding noncoding RNAs (ENSG00000270605 and ENSG00000272977) between the caudate nucleus and suprapubic skin and spleen GTEx tissues, respectively. Relatively low replication rate with GTEx brain regions can, in part, be attributed to low sample sizes in the GTEx dataset (ref 41).

We also replicated across brain regions. Remarkably, all of the shared si-eQTL showed concordant directionality. Furthermore, the DLPFC and hippocampus showed nearly identical si-eQTL effect sizes (Fig. 4B), which was confirmed with the high level of replication across brain regions ($\pi1 > 0.996$; Fig. S28).

Randomization: This is an observational study from postmortem human brain tissues. As such, subjects were not randomized into outcome groups.

Blinding: Investigators were not blinded to group allocation since the study is observational.

Reporting for specific materials, systems and methods

We require information from authors about some types of materials, experimental systems and methods used in many studies. Here, indicate whether each material, system or method listed is relevant to your study. If you are not sure if a list item applies to your research, read the appropriate section before selecting a response.

Materials & experimental systems

n/a	Involved in the study
☒	☐ Antibodies
☒	☐ Eukaryotic cell lines
☒	☐ Palaeontology and archaeology
☒	☐ Animals and other organisms
☒	☐ Clinical data
☒	☐ Dual use research of concern
☒	☐ Plants

Methods

n/a	Involved in the study
☒	☐ ChIP-seq
☒	☐ Flow cytometry
☒	☐ MRI-based neuroimaging

Plants

Seed stocks: *Report on the source of all seed stocks or other plant material used. If applicable, state the seed stock centre and catalogue number. If plant specimens were collected from the field, describe the collection location, date and sampling procedures.*

Novel plant genotypes: *Describe the methods by which all novel plant genotypes were produced. This includes those generated by transgenic approaches, gene editing, chemical/radiation-based mutagenesis and hybridization. For transgenic lines, describe the transformation method, the number of independent lines analyzed and the generation upon which experiments were performed. For gene-edited lines, describe the editor used, the endogenous sequence targeted for editing, the targeting guide RNA sequence (if applicable) and how the editor was applied.*

Authentication: *Describe any authentication procedures for each seed stock used or novel genotype generated. Describe any experiments used to assess the effect of a mutation and, where applicable, how potential secondary effects (e.g. second site T-DNA insertions, mosiacism, off-target gene editing) were examined.*
