# Supplementary material for: Sex affects transcriptional associations with schizophrenia across the dorsolateral prefrontal cortex, hippocampus, and caudate nucleus
Source: Nat Commun. 2024 May 10;15:3980. doi: 10.1038/s41467-024-48048-z (PMC11087501; doi:10.1038/s41467-024-48048-z)

Module kME−Trait Correlation

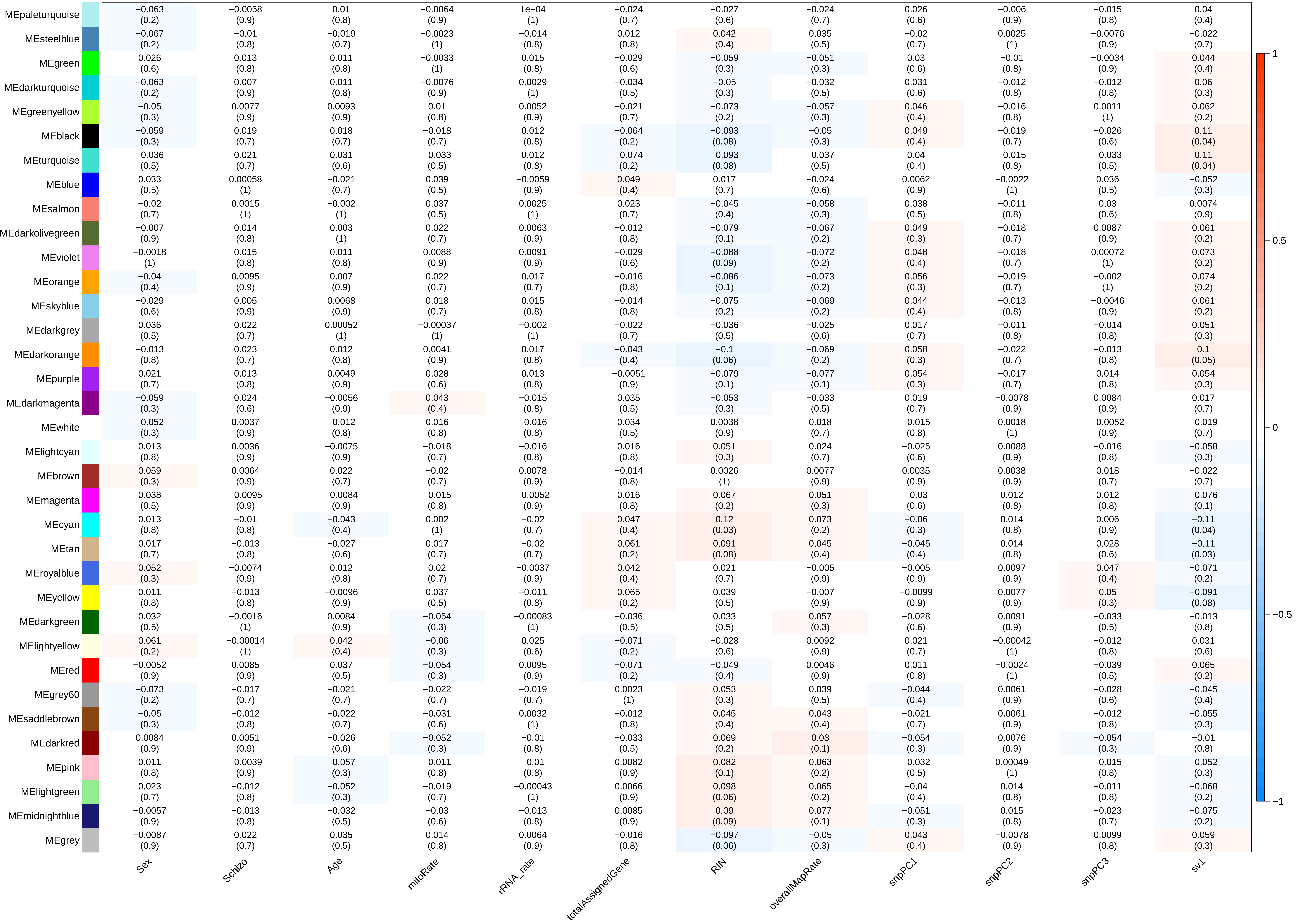

Supplement: Supplementary file 7 — Data S4 [file 41467_2024_48048_MOESM7_ESM.gz › wgcna_network_analysis/autosomal_sex_network/hippocampus/module_trait_relationships.pdf]
