# Supplementary material for: Sex affects transcriptional associations with schizophrenia across the dorsolateral prefrontal cortex, hippocampus, and caudate nucleus
Source: Nat Commun. 2024 May 10;15:3980. doi: 10.1038/s41467-024-48048-z (PMC11087501; doi:10.1038/s41467-024-48048-z)
Supplement: Supplementary file 7 — Data S4 [file 41467_2024_48048_MOESM7_ESM.gz › wgcna_network_analysis/autosomal_sex_network/hippocampus/wgcna_module_enrichment_DEG.pdf]

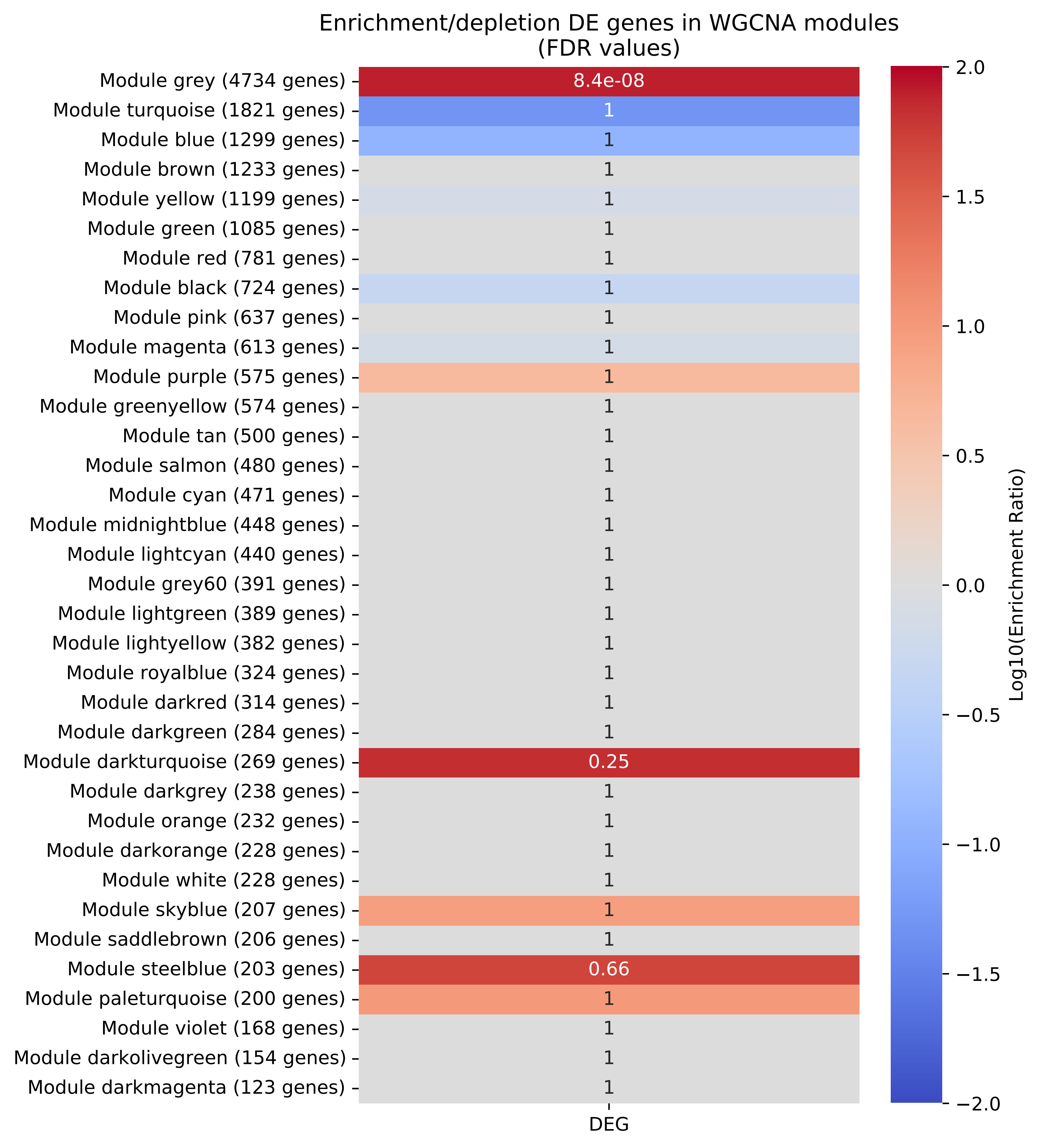

Enrichment/depletion DE genes in WGCNA modules
(FDR values)
Module grey (4734 genes)
8.4e-08
Module turquoise (1821 genes)
1
Module blue (1299 genes)
1
Module brown (1233 genes)
1
Module yellow (1199 genes)
1
Module green (1085 genes)
1
Module red (781 genes)
1
Module black (724 genes)
1
Module pink (637 genes)
1
Module magenta (613 genes)
1
Module purple (575 genes)
1
Module greenyellow (574 genes)
1
Module tan (500 genes)
1
Module salmon (480 genes)
1
Module cyan (471 genes)
1
Module midnightblue (448 genes)
1
Module lightcyan (440 genes)
1
Module grey60 (391 genes)
1
Module lightgreen (389 genes)
1
Module lightyellow (382 genes)
1
Module royalblue (324 genes)
1
Module darkred (314 genes)
1
Module darkgreen (284 genes)
1
Module darkturquoise (269 genes)
0.25
Module darkgrey (238 genes)
1
Module orange (232 genes)
1
Module darkorange (228 genes)
1
Module white (228 genes)
1
Module skyblue (207 genes)
1
Module saddlebrown (206 genes)
1
Module steelblue (203 genes)
0.66
Module paleturquoise (200 genes)
1
Module violet (168 genes)
1
Module darkolivegreen (154 genes)
1
Module darkmagenta (123 genes)
1
DEG
Log10(Enrichment Ratio)
2.0
1.5
1.0
0.5
0.0
−0.5
−1.0
−1.5
−2.0
