# Supplementary material for: Sex affects transcriptional associations with schizophrenia across the dorsolateral prefrontal cortex, hippocampus, and caudate nucleus
Source: Nat Commun. 2024 May 10;15:3980. doi: 10.1038/s41467-024-48048-z (PMC11087501; doi:10.1038/s41467-024-48048-z)
Supplement: Supplementary file 7 — Data S4 [file 41467_2024_48048_MOESM7_ESM.gz › wgcna_network_analysis/autosomal_sex_network/dlpfc/module_trait_relationships.pdf]

Module kME−Trait Correlation

	Sex	Schizo	Age	mitoRate	rRNA_rate	totalAssignedGene	RIN	overallMapRate	snpPC1	snpPC2	snpPC3	sv1
MEmidnightblue	0.014 (0.8)	−0.035 (0.5)	−0.0033 (1)	−0.034 (0.5)	−0.00049 (1)	0.02 (0.7)	0.053 (0.3)	0.057 (0.3)	0.026 (0.6)	0.0082 (0.9)	0.025 (0.6)	0.052 (0.3)
MEblack	−0.057 (0.3)	−0.0057 (0.9)	0.012 (0.8)	0.0055 (0.9)	0.01 (0.9)	0.058 (0.3)	0.075 (0.2)	0.078 (0.1)	−0.034 (0.5)	0.037 (0.5)	0.02 (0.7)	0.11 (0.03)
MEgreen	0.016 (0.8)	0.011 (0.8)	0.0016 (1)	0.014 (0.8)	−0.025 (0.6)	0.05 (0.3)	0.059 (0.3)	0.028 (0.6)	−0.023 (0.7)	0.021 (0.7)	0.011 (0.8)	0.076 (0.2)
MEcyan	−0.036 (0.5)	−0.015 (0.8)	−0.0082 (0.9)	−0.051 (0.3)	−0.0013 (1)	0.0056 (0.9)	0.034 (0.5)	0.065 (0.2)	0.00055 (1)	0.015 (0.8)	0.014 (0.8)	0.038 (0.5)
MElightyellow	0.0043 (0.9)	−0.012 (0.8)	−0.011 (0.8)	−0.051 (0.3)	−0.008 (0.9)	−0.0057 (0.9)	0.008 (0.9)	0.048 (0.4)	0.003 (1)	0.007 (0.9)	0.015 (0.8)	0.01 (0.8)
MEmagenta	−0.067 (0.2)	0.011 (0.8)	0.043 (0.4)	0.031 (0.6)	0.026 (0.6)	0.041 (0.4)	0.041 (0.4)	0.051 (0.3)	−0.054 (0.3)	0.03 (0.6)	0.013 (0.8)	0.088 (0.1)
MEturquoise	−0.084 (0.1)	0.011 (0.8)	0.032 (0.6)	0.037 (0.5)	0.0098 (0.9)	0.052 (0.3)	0.048 (0.4)	0.05 (0.4)	−0.053 (0.3)	0.034 (0.5)	0.014 (0.8)	0.087 (0.1)
MEgreenyellow	−0.069 (0.2)	−0.013 (0.8)	0.0069 (0.9)	−0.009 (0.9)	0.038 (0.5)	−0.0069 (0.9)	0.055 (0.3)	0.057 (0.3)	−0.039 (0.5)	0.018 (0.7)	−0.023 (0.7)	0.044 (0.4)
MElightcyan	−0.059 (0.3)	0.029 (0.6)	0.039 (0.5)	0.013 (0.8)	0.03 (0.6)	0.049 (0.4)	0.058 (0.3)	0.041 (0.4)	−0.073 (0.2)	0.013 (0.8)	−0.037 (0.5)	0.064 (0.2)
MEbrown	0.1 (0.05)	0.0069 (0.9)	−0.023 (0.7)	−0.023 (0.7)	−0.058 (0.3)	0.0015 (1)	0.0069 (0.9)	−0.027 (0.6)	0.026 (0.6)	−0.017 (0.8)	−0.0047 (0.9)	−0.037 (0.5)
MEpink	0.039 (0.5)	−0.016 (0.8)	−0.041 (0.4)	−0.068 (0.2)	−0.053 (0.3)	−0.095 (0.07)	−0.12 (0.02)	−0.1 (0.06)	0.092 (0.09)	−0.063 (0.2)	−0.022 (0.7)	−0.2 (1e−04)
MEyellow	0.092 (0.09)	−0.0058 (0.9)	−0.037 (0.5)	−0.061 (0.3)	−0.064 (0.2)	−0.07 (0.2)	−0.095 (0.08)	−0.085 (0.1)	0.078 (0.1)	−0.054 (0.3)	−0.022 (0.7)	−0.17 (0.002)
MEroyalblue	0.017 (0.7)	0.019 (0.7)	0.036 (0.5)	0.0059 (0.9)	0.0085 (0.9)	0.016 (0.8)	0.0083 (0.9)	0.0085 (0.9)	−0.025 (0.6)	0.008 (0.9)	−0.0087 (0.9)	0.026 (0.6)
MEgrey60	0.095 (0.08)	0.0072 (0.9)	−0.0082 (0.9)	−0.033 (0.5)	−0.023 (0.7)	−0.066 (0.2)	−0.09 (0.09)	−0.077 (0.1)	0.05 (0.3)	−0.039 (0.5)	−0.015 (0.8)	−0.13 (0.01)
MElightgreen	0.12 (0.03)	0.017 (0.7)	−0.01 (0.8)	−0.03 (0.6)	−0.054 (0.3)	−0.017 (0.7)	−0.021 (0.7)	−0.044 (0.4)	0.029 (0.6)	−0.027 (0.6)	−0.018 (0.7)	−0.075 (0.2)
MEblue	0.03 (0.6)	−0.0091 (0.9)	−0.0068 (0.9)	−0.039 (0.5)	−0.013 (0.8)	−0.071 (0.2)	−0.083 (0.1)	−0.051 (0.3)	0.043 (0.4)	−0.035 (0.5)	−0.016 (0.8)	−0.11 (0.04)
MEred	−0.0078 (0.9)	−0.02 (0.7)	−0.029 (0.6)	−0.059 (0.3)	−0.03 (0.6)	−0.089 (0.1)	−0.12 (0.03)	−0.08 (0.1)	0.073 (0.2)	−0.055 (0.3)	−0.021 (0.7)	−0.17 (0.001)
MEtan	−0.08 (0.1)	−0.0059 (0.9)	0.017 (0.7)	0.011 (0.8)	0.014 (0.8)	−0.0034 (0.9)	0.0093 (0.9)	0.012 (0.8)	−0.0058 (0.9)	0.0019 (1)	0.008 (0.9)	0.018 (0.7)
MEpurple	−0.019 (0.7)	−0.0097 (0.9)	0.037 (0.5)	−0.001 (1)	0.024 (0.7)	0.019 (0.7)	0.024 (0.7)	0.055 (0.3)	−0.024 (0.7)	0.014 (0.8)	0.012 (0.8)	0.059 (0.3)
MEsalmon	0.047 (0.4)	0.011 (0.8)	0.034 (0.5)	0.004 (0.9)	0.013 (0.8)	−0.017 (0.8)	−0.034 (0.5)	−0.0059 (0.9)	−0.012 (0.8)	−0.0023 (1)	−0.0029 (1)	−0.003 (1)
MEgrey	0.027 (0.6)	−0.04 (0.5)	0.0055 (0.9)	−0.038 (0.5)	0.02 (0.7)	−0.0039 (0.9)	0.0075 (0.9)	0.046 (0.4)	0.036 (0.5)	−0.002 (1)	0.017 (0.7)	0.017 (0.7)

Colour scale: 1, 0.5, 0, −0.5, −1
