# Supplementary material for: Sex affects transcriptional associations with schizophrenia across the dorsolateral prefrontal cortex, hippocampus, and caudate nucleus
Source: Nat Commun. 2024 May 10;15:3980. doi: 10.1038/s41467-024-48048-z (PMC11087501; doi:10.1038/s41467-024-48048-z)
Supplement: Supplementary file 7 — Data S4 [file 41467_2024_48048_MOESM7_ESM.gz › wgcna_network_analysis/autosomal_sex_network/dlpfc/wgcna_module_enrichment_DEG.pdf]

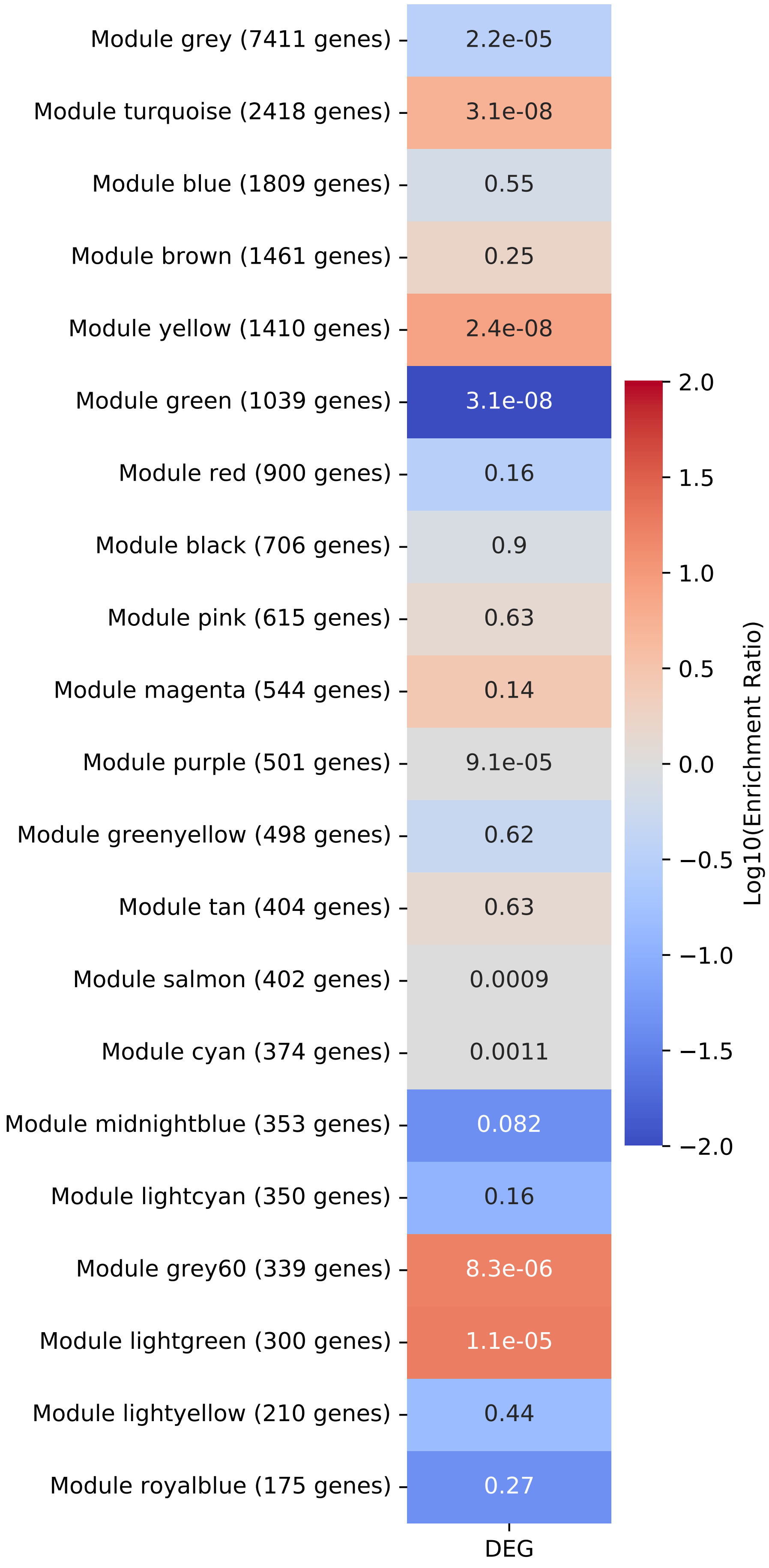
Module grey (7411 genes)
2.2e-05
Module turquoise (2418 genes)
3.1e-08
Module blue (1809 genes)
0.55
Module brown (1461 genes)
0.25
Module yellow (1410 genes)
2.4e-08
Module green (1039 genes)
3.1e-08
Module red (900 genes)
0.16
Module black (706 genes)
0.9
Module pink (615 genes)
0.63
Module magenta (544 genes)
0.14
Module purple (501 genes)
9.1e-05
Module greenyellow (498 genes)
0.62
Module tan (404 genes)
0.63
Module salmon (402 genes)
0.0009
Module cyan (374 genes)
0.0011
Module midnightblue (353 genes)
0.082
Module lightcyan (350 genes)
0.16
Module grey60 (339 genes)
8.3e-06
Module lightgreen (300 genes)
1.1e-05
Module lightyellow (210 genes)
0.44
Module royalblue (175 genes)
0.27
DEG
2.0
1.5
1.0
0.5
0.0
−0.5
−1.0
−1.5
−2.0
Log10(Enrichment Ratio)
