# Supplementary material for: Sex affects transcriptional associations with schizophrenia across the dorsolateral prefrontal cortex, hippocampus, and caudate nucleus
Source: Nat Commun. 2024 May 10;15:3980. doi: 10.1038/s41467-024-48048-z (PMC11087501; doi:10.1038/s41467-024-48048-z)
Supplement: Supplementary file 7 — Data S4 [file 41467_2024_48048_MOESM7_ESM.gz › wgcna_network_analysis/autosomal_sex_network/dlpfc/power_parameter_selection.pdf]

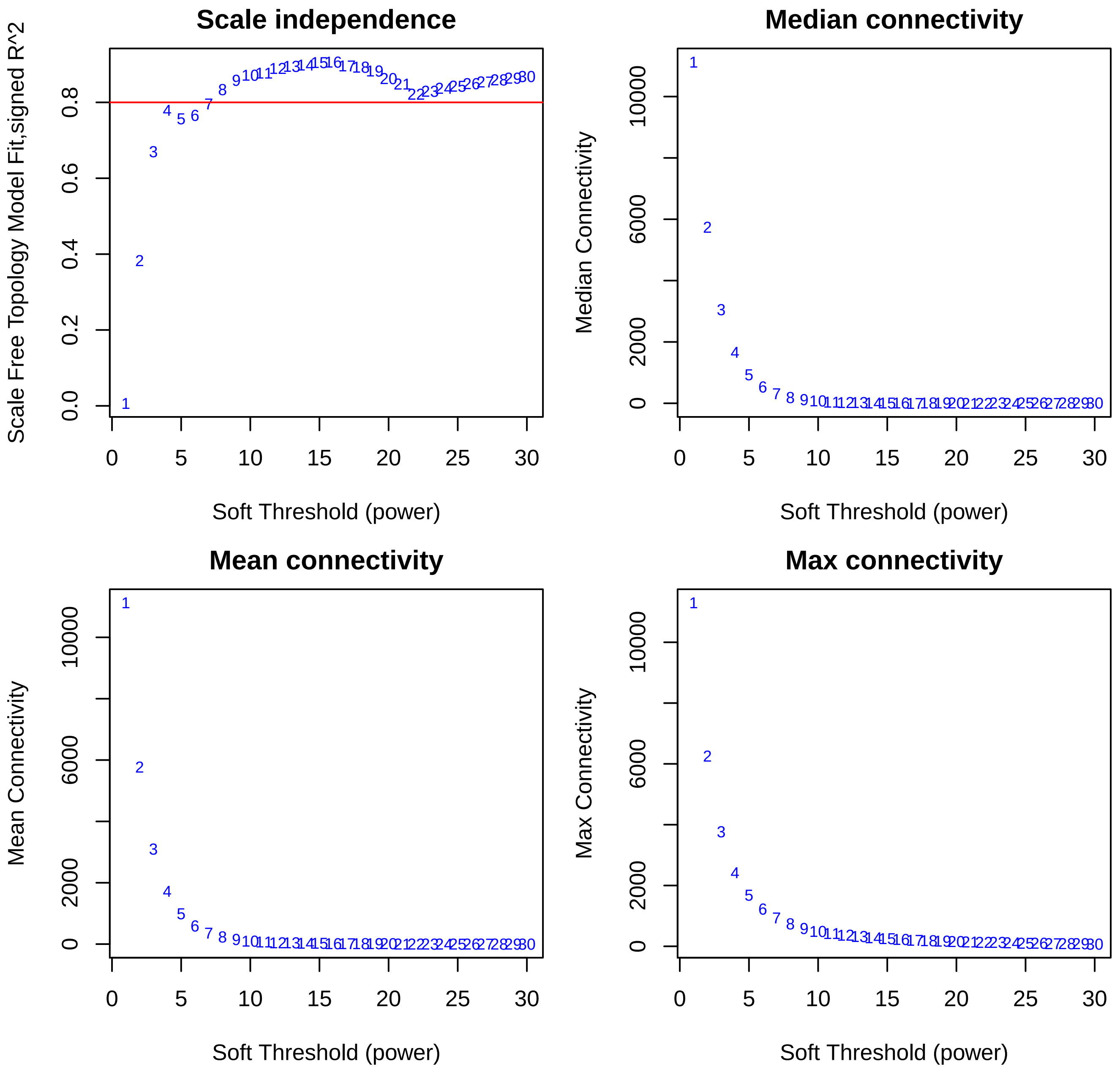
Scale independence
Scale Free Topology Model Fit,signed R^2
Soft Threshold (power)
Median connectivity
Median Connectivity
Soft Threshold (power)
Mean connectivity
Mean Connectivity
Soft Threshold (power)
Max connectivity
Max Connectivity
Soft Threshold (power)
