# Supplementary material for: Sex affects transcriptional associations with schizophrenia across the dorsolateral prefrontal cortex, hippocampus, and caudate nucleus
Source: Nat Commun. 2024 May 10;15:3980. doi: 10.1038/s41467-024-48048-z (PMC11087501; doi:10.1038/s41467-024-48048-z)
Supplement: Supplementary file 7 — Data S4 [file 41467_2024_48048_MOESM7_ESM.gz › wgcna_network_analysis/autosomal_sex_network/caudate/module_trait_relationships.pdf]

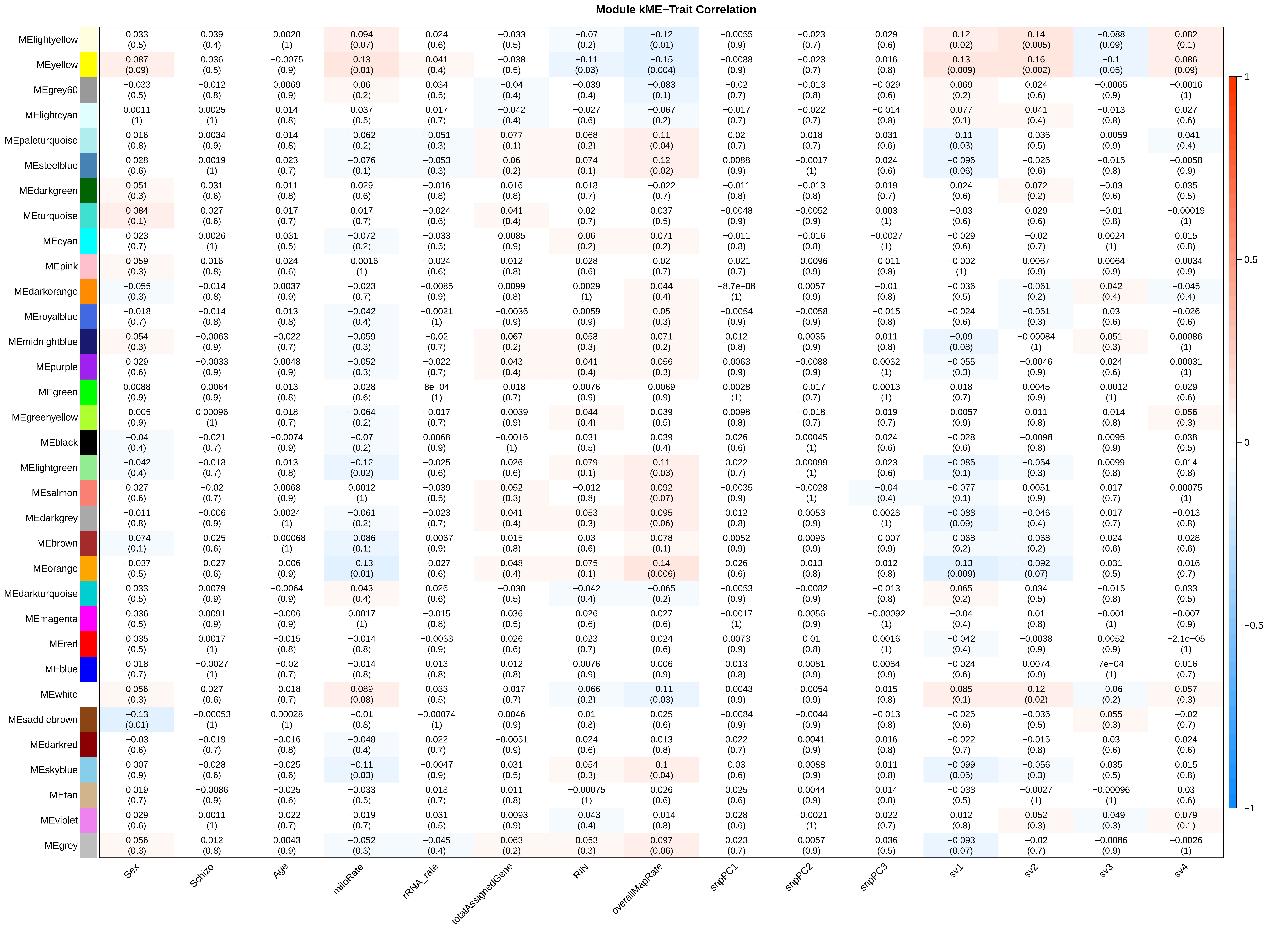
Module kME−Trait Correlation
Sex Schizo Age mitoRate rRNA_rate totalAssignedGene RIN overallMapRate snpPC1 snpPC2 snpPC3 sv1 sv2 sv3 sv4
MElightyellow 0.033 (0.5) 0.039 (0.4) 0.0028 (1) 0.094 (0.07) 0.024 (0.6) −0.033 (0.5) −0.07 (0.2) −0.12 (0.01) −0.0055 (0.9) −0.023 (0.7) 0.029 (0.6) 0.12 (0.02) 0.14 (0.005) −0.088 (0.09) 0.082 (0.1)
MEyellow 0.087 (0.09) 0.036 (0.5) −0.0075 (0.9) 0.13 (0.01) 0.041 (0.4) −0.038 (0.5) −0.11 (0.03) −0.15 (0.004) −0.0088 (0.9) −0.023 (0.7) 0.016 (0.8) 0.13 (0.009) 0.16 (0.002) −0.1 (0.05) 0.086 (0.09)
MEgrey60 −0.033 (0.5) −0.012 (0.8) 0.0069 (0.9) 0.06 (0.2) 0.034 (0.5) −0.04 (0.4) −0.039 (0.4) −0.083 (0.1) −0.02 (0.7) −0.013 (0.8) −0.029 (0.6) 0.069 (0.2) 0.024 (0.6) −0.0065 (0.9) −0.0016 (1)
MElightcyan 0.0011 (1) 0.0025 (1) 0.014 (0.8) 0.037 (0.5) 0.017 (0.7) −0.042 (0.4) −0.027 (0.6) −0.067 (0.2) −0.017 (0.7) −0.022 (0.7) −0.014 (0.8) 0.077 (0.1) 0.041 (0.4) −0.013 (0.8) 0.027 (0.6)
MEpaleturquoise 0.016 (0.8) 0.0034 (0.9) 0.014 (0.8) −0.062 (0.2) −0.051 (0.3) 0.077 (0.1) 0.068 (0.2) 0.11 (0.04) 0.02 (0.7) 0.018 (0.7) 0.031 (0.6) −0.11 (0.03) −0.036 (0.5) −0.0059 (0.9) −0.041 (0.4)
MEsteelblue 0.028 (0.6) 0.0019 (1) 0.023 (0.7) −0.076 (0.1) −0.053 (0.3) 0.06 (0.2) 0.074 (0.1) 0.12 (0.02) 0.0088 (0.9) −0.0017 (1) 0.024 (0.6) −0.096 (0.06) −0.026 (0.6) −0.015 (0.8) −0.0058 (0.9)
MEdarkgreen 0.051 (0.3) 0.031 (0.6) 0.011 (0.8) 0.029 (0.6) −0.016 (0.8) 0.016 (0.8) 0.018 (0.7) −0.022 (0.7) −0.011 (0.8) −0.013 (0.8) 0.019 (0.7) 0.024 (0.6) 0.072 (0.2) −0.03 (0.6) 0.035 (0.5)
MEturquoise 0.084 (0.1) 0.027 (0.6) 0.017 (0.7) 0.017 (0.7) −0.024 (0.6) 0.041 (0.4) 0.02 (0.7) 0.037 (0.5) −0.0048 (0.9) −0.0052 (0.9) 0.003 (1) −0.03 (0.6) 0.029 (0.6) −0.01 (0.8) −0.00019 (1)
MEcyan 0.023 (0.7) 0.0026 (1) 0.031 (0.5) −0.072 (0.2) −0.033 (0.5) 0.0085 (0.9) 0.06 (0.2) 0.071 (0.2) −0.011 (0.8) −0.016 (0.8) −0.0027 (1) −0.029 (0.6) −0.02 (0.7) 0.0024 (1) 0.015 (0.8)
MEpink 0.059 (0.3) 0.016 (0.8) 0.024 (0.6) −0.0016 (1) −0.024 (0.6) 0.012 (0.8) 0.028 (0.6) 0.02 (0.7) −0.021 (0.7) −0.0096 (0.9) −0.011 (0.8) −0.002 (1) 0.0067 (0.9) 0.0064 (0.9) −0.0034 (0.9)
MEdarkorange −0.055 (0.3) −0.014 (0.8) 0.0037 (0.9) −0.023 (0.7) −0.0085 (0.9) 0.0099 (0.8) 0.0029 (1) 0.044 (0.4) −8.7e−08 (1) 0.0057 (0.9) −0.01 (0.8) −0.036 (0.5) −0.061 (0.2) 0.042 (0.4) −0.045 (0.4)
MEroyalblue −0.018 (0.7) −0.014 (0.8) 0.013 (0.8) −0.042 (0.4) −0.0021 (1) −0.0036 (0.9) 0.0059 (0.9) 0.05 (0.3) −0.0054 (0.9) −0.0058 (0.9) −0.015 (0.8) −0.024 (0.6) −0.051 (0.3) 0.03 (0.6) −0.026 (0.6)
MEmidnightblue 0.054 (0.3) −0.0063 (0.9) −0.022 (0.7) −0.059 (0.3) −0.02 (0.7) 0.067 (0.2) 0.058 (0.3) 0.071 (0.2) 0.012 (0.8) 0.0035 (0.9) 0.011 (0.8) −0.09 (0.08) −0.00084 (1) 0.051 (0.3) 0.00086 (1)
MEpurple 0.029 (0.6) −0.0033 (0.9) 0.0048 (0.9) −0.052 (0.3) −0.022 (0.7) 0.043 (0.4) 0.041 (0.4) 0.056 (0.3) 0.0063 (0.9) −0.0088 (0.9) 0.0032 (1) −0.055 (0.3) −0.0046 (0.9) 0.024 (0.6) 0.00031 (1)
MEgreen 0.0088 (0.9) −0.0064 (0.9) 0.013 (0.8) −0.028 (0.6) 8e−04 (1) −0.018 (0.7) 0.0076 (0.9) 0.0069 (0.9) 0.0028 (1) −0.017 (0.7) 0.0013 (1) 0.018 (0.7) 0.0045 (0.9) −0.0012 (1) 0.029 (0.6)
MEgreenyellow −0.005 (0.9) 0.00096 (1) 0.018 (0.7) −0.064 (0.2) −0.017 (0.7) −0.0039 (0.9) 0.044 (0.4) 0.039 (0.5) 0.0098 (0.8) −0.018 (0.7) 0.019 (0.7) −0.0057 (0.9) 0.011 (0.8) −0.014 (0.8) 0.056 (0.3)
MEblack −0.04 (0.4) −0.021 (0.7) −0.0074 (0.9) −0.07 (0.2) 0.0068 (0.9) −0.0016 (1) 0.031 (0.5) 0.039 (0.4) 0.026 (0.6) 0.00045 (1) 0.024 (0.6) −0.028 (0.6) −0.0098 (0.8) 0.0095 (0.9) 0.038 (0.5)
MElightgreen −0.042 (0.4) −0.018 (0.7) 0.013 (0.8) −0.12 (0.02) −0.025 (0.6) 0.026 (0.6) 0.079 (0.1) 0.11 (0.03) 0.022 (0.7) 0.00099 (1) 0.023 (0.6) −0.085 (0.1) −0.054 (0.3) 0.0099 (0.8) 0.014 (0.8)
MEsalmon 0.027 (0.6) −0.02 (0.7) 0.0068 (0.9) 0.0012 (1) −0.039 (0.5) 0.052 (0.3) −0.012 (0.8) 0.092 (0.07) −0.0035 (0.9) −0.0028 (1) −0.04 (0.4) −0.077 (0.1) 0.0051 (0.9) 0.017 (0.7) 0.00075 (1)
MEdarkgrey −0.011 (0.8) −0.006 (0.9) 0.0024 (1) −0.061 (0.2) −0.023 (0.7) 0.041 (0.4) 0.053 (0.3) 0.095 (0.06) 0.012 (0.8) 0.0053 (0.9) 0.0028 (1) −0.088 (0.09) −0.046 (0.4) 0.017 (0.7) −0.013 (0.8)
MEbrown −0.074 (0.1) −0.025 (0.6) −0.00068 (1) −0.086 (0.1) −0.0067 (0.9) 0.015 (0.8) 0.03 (0.6) 0.078 (0.1) 0.0052 (0.9) 0.0096 (0.9) −0.007 (0.9) −0.068 (0.2) −0.068 (0.2) 0.024 (0.6) −0.028 (0.6)
MEorange −0.037 (0.5) −0.027 (0.6) −0.006 (0.9) −0.13 (0.01) −0.027 (0.6) 0.048 (0.4) 0.075 (0.1) 0.14 (0.006) 0.026 (0.6) 0.013 (0.8) 0.012 (0.8) −0.13 (0.009) −0.092 (0.07) 0.031 (0.5) −0.016 (0.7)
MEdarkturquoise 0.033 (0.5) 0.0079 (0.9) −0.0064 (0.9) 0.043 (0.4) 0.026 (0.6) −0.038 (0.5) −0.042 (0.4) −0.065 (0.2) −0.0053 (0.9) −0.0082 (0.9) −0.013 (0.8) 0.065 (0.2) 0.034 (0.5) −0.015 (0.8) 0.033 (0.5)
MEmagenta 0.036 (0.5) 0.0091 (0.9) −0.006 (0.9) 0.0017 (1) −0.015 (0.8) 0.036 (0.5) 0.026 (0.6) 0.027 (0.6) −0.0017 (1) 0.0056 (0.9) −0.00092 (1) −0.04 (0.4) 0.01 (0.8) −0.001 (1) −0.007 (0.9)
MEred 0.035 (0.5) 0.0017 (1) −0.015 (0.8) −0.014 (0.8) −0.0033 (0.9) 0.026 (0.6) 0.023 (0.7) 0.024 (0.6) 0.0073 (0.9) 0.01 (0.8) 0.0016 (1) −0.042 (0.4) −0.0038 (0.9) 0.0052 (0.9) −2.1e−05 (1)
MEblue 0.018 (0.7) −0.0027 (1) −0.02 (0.7) −0.014 (0.8) 0.013 (0.8) 0.012 (0.8) 0.0076 (0.9) 0.006 (0.9) 0.013 (0.8) 0.0081 (0.9) 0.0084 (0.9) −0.024 (0.6) 0.0074 (0.9) 7e−04 (1) 0.016 (0.7)
MEwhite 0.056 (0.3) 0.027 (0.6) −0.018 (0.7) 0.089 (0.08) 0.033 (0.5) −0.017 (0.7) −0.066 (0.2) −0.11 (0.03) −0.0043 (0.9) −0.0054 (0.9) 0.015 (0.8) 0.085 (0.1) 0.12 (0.02) −0.06 (0.2) 0.057 (0.3)
MEsaddlebrown −0.13 (0.01) −0.00053 (1) 0.00028 (1) −0.01 (0.8) −0.00074 (1) 0.0046 (0.9) 0.01 (0.8) 0.025 (0.6) −0.0084 (0.9) −0.0044 (0.9) −0.013 (0.8) −0.025 (0.6) −0.036 (0.5) 0.055 (0.3) −0.02 (0.7)
MEdarkred −0.03 (0.6) −0.019 (0.7) −0.016 (0.8) −0.048 (0.4) 0.022 (0.7) −0.0051 (0.9) 0.024 (0.6) 0.013 (0.8) 0.022 (0.7) 0.0041 (0.9) 0.016 (0.8) −0.022 (0.7) −0.015 (0.8) 0.03 (0.6) 0.024 (0.6)
MEskyblue 0.007 (0.9) −0.028 (0.6) −0.025 (0.6) −0.11 (0.03) −0.0047 (0.9) 0.031 (0.5) 0.054 (0.3) 0.1 (0.04) 0.03 (0.6) 0.0088 (0.9) 0.011 (0.8) −0.099 (0.05) −0.056 (0.3) 0.035 (0.5) 0.015 (0.8)
MEtan 0.019 (0.7) −0.0086 (0.9) −0.025 (0.6) −0.033 (0.5) 0.018 (0.7) 0.011 (0.8) −0.00075 (1) 0.026 (0.6) 0.025 (0.6) 0.0044 (0.9) 0.014 (0.8) −0.038 (0.5) −0.0027 (1) −0.00096 (1) 0.03 (0.6)
MEviolet 0.029 (0.6) 0.0011 (1) −0.022 (0.7) −0.019 (0.7) 0.031 (0.5) −0.0093 (0.9) −0.043 (0.4) −0.014 (0.8) 0.028 (0.6) −0.0021 (1) 0.022 (0.7) 0.012 (0.8) 0.052 (0.3) −0.049 (0.3) 0.079 (0.1)
MEgrey 0.056 (0.3) 0.012 (0.8) 0.0043 (0.9) −0.052 (0.3) −0.045 (0.4) 0.063 (0.2) 0.053 (0.3) 0.097 (0.06) 0.023 (0.7) 0.0057 (0.9) 0.036 (0.5) −0.093 (0.07) −0.02 (0.7) −0.0086 (0.9) −0.0026 (1)
1
0.5
0
−0.5
−1
