# Supplementary material for: Sex affects transcriptional associations with schizophrenia across the dorsolateral prefrontal cortex, hippocampus, and caudate nucleus
Source: Nat Commun. 2024 May 10;15:3980. doi: 10.1038/s41467-024-48048-z (PMC11087501; doi:10.1038/s41467-024-48048-z)
Supplement: Supplementary file 7 — Data S4 [file 41467_2024_48048_MOESM7_ESM.gz › wgcna_network_analysis/autosomal_sex_network/caudate/wgcna_module_enrichment_DEG_noMHC.pdf]

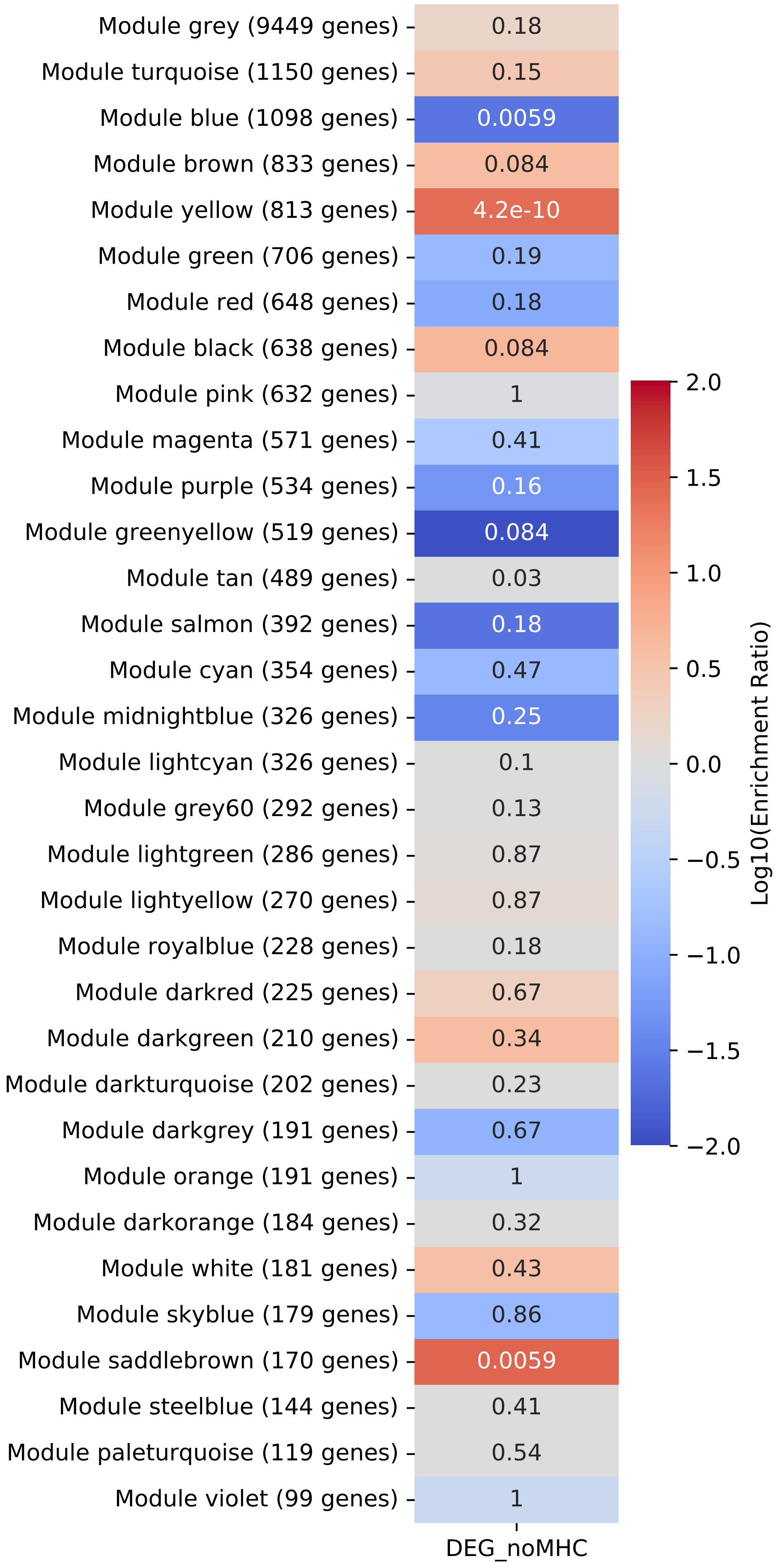

Module grey (9449 genes)
Module turquoise (1150 genes)
Module blue (1098 genes)
Module brown (833 genes)
Module yellow (813 genes)
Module green (706 genes)
Module red (648 genes)
Module black (638 genes)
Module pink (632 genes)
Module magenta (571 genes)
Module purple (534 genes)
Module greenyellow (519 genes)
Module tan (489 genes)
Module salmon (392 genes)
Module cyan (354 genes)
Module midnightblue (326 genes)
Module lightcyan (326 genes)
Module grey60 (292 genes)
Module lightgreen (286 genes)
Module lightyellow (270 genes)
Module royalblue (228 genes)
Module darkred (225 genes)
Module darkgreen (210 genes)
Module darkturquoise (202 genes)
Module darkgrey (191 genes)
Module orange (191 genes)
Module darkorange (184 genes)
Module white (181 genes)
Module skyblue (179 genes)
Module saddlebrown (170 genes)
Module steelblue (144 genes)
Module paleturquoise (119 genes)
Module violet (99 genes)
0.18
0.15
0.0059
0.084
4.2e-10
0.19
0.18
0.084
1
0.41
0.16
0.084
0.03
0.18
0.47
0.25
0.1
0.13
0.87
0.87
0.18
0.67
0.34
0.23
0.67
1
0.32
0.43
0.86
0.0059
0.41
0.54
1
DEG_noMHC
2.0
1.5
1.0
0.5
0.0
−0.5
−1.0
−1.5
−2.0
Log10(Enrichment Ratio)
