# Supplementary material for: Sex affects transcriptional associations with schizophrenia across the dorsolateral prefrontal cortex, hippocampus, and caudate nucleus
Source: Nat Commun. 2024 May 10;15:3980. doi: 10.1038/s41467-024-48048-z (PMC11087501; doi:10.1038/s41467-024-48048-z)
Supplement: Supplementary file 7 — Data S4 [file 41467_2024_48048_MOESM7_ESM.gz › wgcna_network_analysis/autosomal_sex_network/caudate/cluster_dendrogram.pdf]

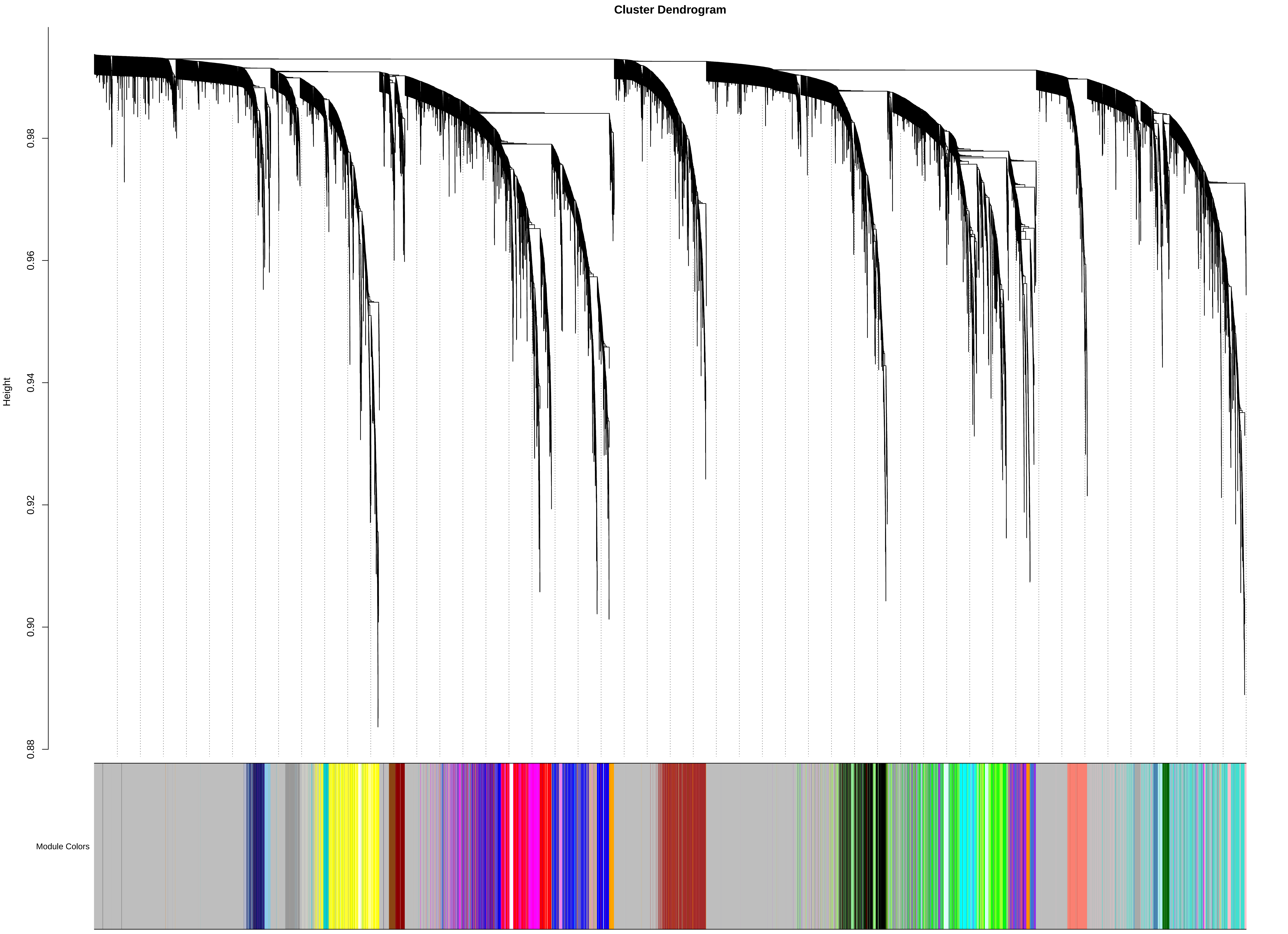
Cluster Dendrogram
Height
0.88
0.90
0.92
0.94
0.96
0.98
Module Colors
